# Ph_2_SCCO: A New Versatile CCO‐Fragment Transfer Reagent

**DOI:** 10.1002/anie.202518689

**Published:** 2025-10-10

**Authors:** Qiu Sun, Julian Hauda, David Tymann, Patrick W. Antoni, Richard Goddard, Max M. Hansmann

**Affiliations:** ^1^ Fakultät für Chemie und Chemische Biologie Technische Universität Dortmund Otto‐Hahn‐Str. 6 44227 Dortmund Germany; ^2^ Key Laboratory of Organic Synthesis of Jiangsu Province College of Chemistry Chemical Engineering and Materials Science Soochow University Suzhou 215123 P.R. China; ^3^ Max‐Planck‐Institut für Kohlenforschung Kaiser‐Wilhelm‐Platz 1 45470 Mülheim an der Ruhr Germany

**Keywords:** Bicyclo[3.1.0]hexanes, CCO fragment transfer, Cyclopropanation, Heterocumulenes, Sulfur ylides

## Abstract

ketenylidene (C_2_O) is an electronically intriguing small molecule with significant potential as a C_2_ synthon in organic synthesis. However, its high reactivity has thus far precluded practical applications. Herein, we report the synthesis of a new reagent, the heterocumulene Ph_2_S═C═C═O (**1**), a well‐defined C_2_O equivalent that accurately replicates the chemical reactivity profile of C_2_O in a controlled manner. **1** uniquely integrates ketene‐like and classical carbene reactivity. In the presence of Brønsted acids, **1** undergoes initial 1,2‐addition to form a sulfur ylide intermediate, which can be exploited for subsequent reactions including cyclopropanation, epoxidation, or X–H addition. The CCO fragment transfer strategy, starting from simple precursors, enables efficient access to α‐cyclopropyl‐ as well as α‐epoxy‐carbonyl derivatives and structurally complex bicyclic cyclopropanes.

## Graphical abstract

The synthesis, isolation, and full characterization of the unprecedented heterocumulene Ph_2_SCCO is presented. Starting from simple precursors, Ph_2_SCCO provides direct access to the valuable class of (bicyclic) α‐cyclopropylcarbonyl compounds. It has emerged from our reactivity studies as an excellent reagent for the CCO fragment transfer in organic synthesis.

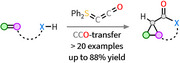

Substituted cyclopropanes have high value as versatile synthetic intermediates, used for constructing a wide range of useful structural motifs,^[^ ^1^, ^2^ ^]^ and they are frequently found in natural products.^[^ ^3^, ^4^ ^]^ They constitute key structural elements in pharmaceutical molecules.^[^ ^5^ ^]^ In fact, cyclopropanes rank as the 10th most commonly used ring system in small‐molecule drugs.^[^ ^6^ ^]^ The structural diversity of substituted cyclopropanes is remarkably broad, ranging from simple monocyclic compounds to complex fused ring systems (Scheme 1a).^[^ ^7^, ^8^, ^9^, ^10^ ^]^ A particularly noteworthy subclass is that of (hetero)bicyclo[3.1.0]hexanes (**I** and **II**), which, due to their highly rigid, sp^3^‐rich core structures, have emerged as valuable building blocks in medicinal chemistry.^[^ ^11^, ^12^, ^13^, ^14^ ^]^

**Scheme 1:**
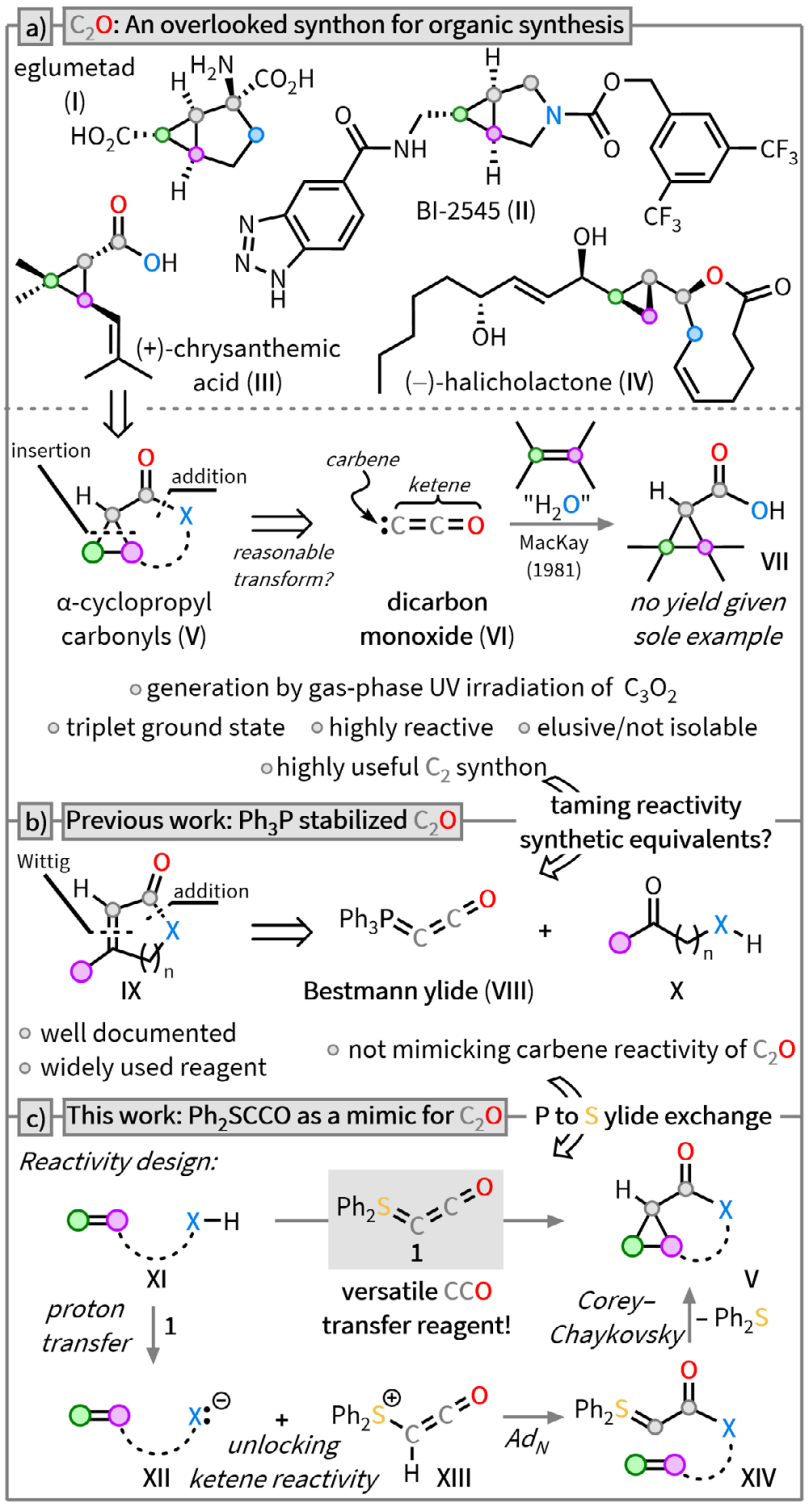
Evolution of a versatile CCO‐transfer reagent. a) Retrosynthetic analysis of selected cyclopropane‐containing molecules and introduction of C_2_O. b) Introduction of the Bestmann ylide **VIII** and discussion of its reactivity. c) Reactivity‐guided design and presentation of Ph_2_SCCO (**1**).

Given the importance of these structural motifs, a wide variety of synthetic methods have been developed.^[^ ^15^ ^]^ Prominent examples are the Simmons–Smith^[^ ^16^ ^]^ and Corey–Chaykovsky cyclopropanations,^[^ ^17^, ^18^ ^]^ as well as transition metal‐catalyzed carbene insertions into olefins using diazo compounds.^[^ ^19^, ^20^ ^]^ For the construction of annulated cyclopropanes, intramolecular cyclopropanation reactions such as enyne cyclizations are particularly well suited, as they enable the formation of two rings in a single step. This elegant strategy is well established for diazo precursors.^[^ ^21^ ^]^ However, intramolecular variants of the classical Corey–Chaykovsky cyclopropanation have only rarely been applied^[^ ^22^, ^23^ ^]^ in this context, and the few reported examples rely on transition metal catalysis.^[^ ^24^, ^25^, ^26^, ^27^, ^28^, ^29^, ^30^ ^]^

From a retrosynthetic perspective, a range of bioactive cyclopropanes can be traced back to α‐cyclopropyl carbonyl compounds (**V**; Scheme 1a). An ideal approach to this structural motif could involve the use of dicarbon monoxide/ketenylidene (C_2_O) (**VI**) as a C_2_ synthon, as **VI** uniquely combines the reactivity of both carbenes and ketenes. Besides its presence in star‐forming regions of the interstellar medium,^[^ ^31^ ^]^ very few reactivity studies on C_2_O have been reported. Bayes et al.,^[^ ^32^ ^]^ and later MacKay et al.,^[^ ^33^ ^]^ demonstrated that carbon suboxide (C_3_O_2_) can generate C_2_O in the gas phase upon UV irradiation. This highly reactive species can, for instance, be trapped with tetramethylethylene to form either the dimer of the intermediate cyclopropylidene ketene or, in the presence of trace amounts of water, the α‐cyclopropyl carboxylic acid **VII**. Despite these promising entries, C_2_O has not found further application as a C_2_ synthon in organic synthesis, which is likely due to the high reactivity as a triplet ground state compound,^[^ ^34^, ^35^, ^36^, ^37^ ^]^ prohibiting its isolation in the condensed phase.^[^ ^38^ ^]^

An alternative strategy involves the use of synthetic equivalents, wherein the reactive fragment is stabilized. In this context, Bestmann ylide (Ph_3_PCCO; **VIII**)^[^ ^39^ ^]^ has found broad application in organic synthesis, primarily exploiting both Wittig‐ and ketene‐type reactivity. **VIII** is commonly employed in the preparation of cyclic (**IX**) or acyclic α,β‐unsaturated carboxylic acid derivatives (Scheme 1b).^[^ ^40^ ^]^ However, since stabilization of the C_2_O moiety in **VIII** is achieved via a P‐ylide, cyclopropanation reactivity is lacking. In contrast to P‐ylides, S‐ylides display carbene‐like reactivity and have been extensively used in cyclopropanation reactions.^[^ ^41^ ^]^ Here, we pursue the synthesis of the unprecedented sulfur‐based analogue Ph_2_S═C═C═O (**1**) (Scheme 1c). **1** should allow for a significantly modulated and controllable expression of C_2_O‐type reactivity establishing both ketene and carbene reactivity. In the presence of acidic substrates, **1** would initially undergo a stepwise 1,2‐addition to form a S‐ylide (**XIV**), which could subsequently react with olefins in a Corey–Chaykovsky‐type reaction to furnish the desired α‐cyclopropyl carbonyl compounds **V**. In the following, we describe the synthesis of **1** and investigate its reactivity with respect to its potential as a CCO transfer reagent.

Classical ketene synthesis strategies, including base‐induced 1,2‐elimination from esters,^[^ ^42^, ^43^ ^]^ as applied in the preparation of Bestmann's ylide (**VIII**), were in our hands unsuccessful for accessing **1**. However, based on previous studies from our group^[^ ^44^, ^45^, ^46^ ^]^ as well as the Severin group,^[^ ^47^ ^]^ it was known that unsaturated diazo compounds of the structure X═C═N_2_ can undergo a formal N_2_/CO exchange upon reaction with carbon monoxide (CO).^[^ ^48^, ^49^, ^50^ ^]^ Therefore, we decided to test the exchange strategy using the recently described reagent Ph_2_S═C═N_2_ (**A**)^[^ ^51^ ^]^ as the starting point for our investigations.

To our delight, the reaction of **A** with CO (1 bar) from −78 °C to 0 °C proceeded with high selectivity, affording the desired heterocumulene **1** as a crystalline colorless solid in 85% yield (Scheme 2). DFT calculations at the PBE0‐D3(BJ)/def2‐TZVP/SMD(THF) level of theory suggest that this transformation proceeds via an asynchronous concerted mechanism, which features a low activation barrier (Δ*G* ^‡^ = +13.5 kcal mol^−1^) and is highly exergonic (Δ*G* ^0^ = −56.0 kcal/mol^−1^) (see Supporting Information), consistent with previous theoretical studies of N_2_/CO exchange in diazoolefins.^[^ ^44^ ^]^

**Scheme 2:**
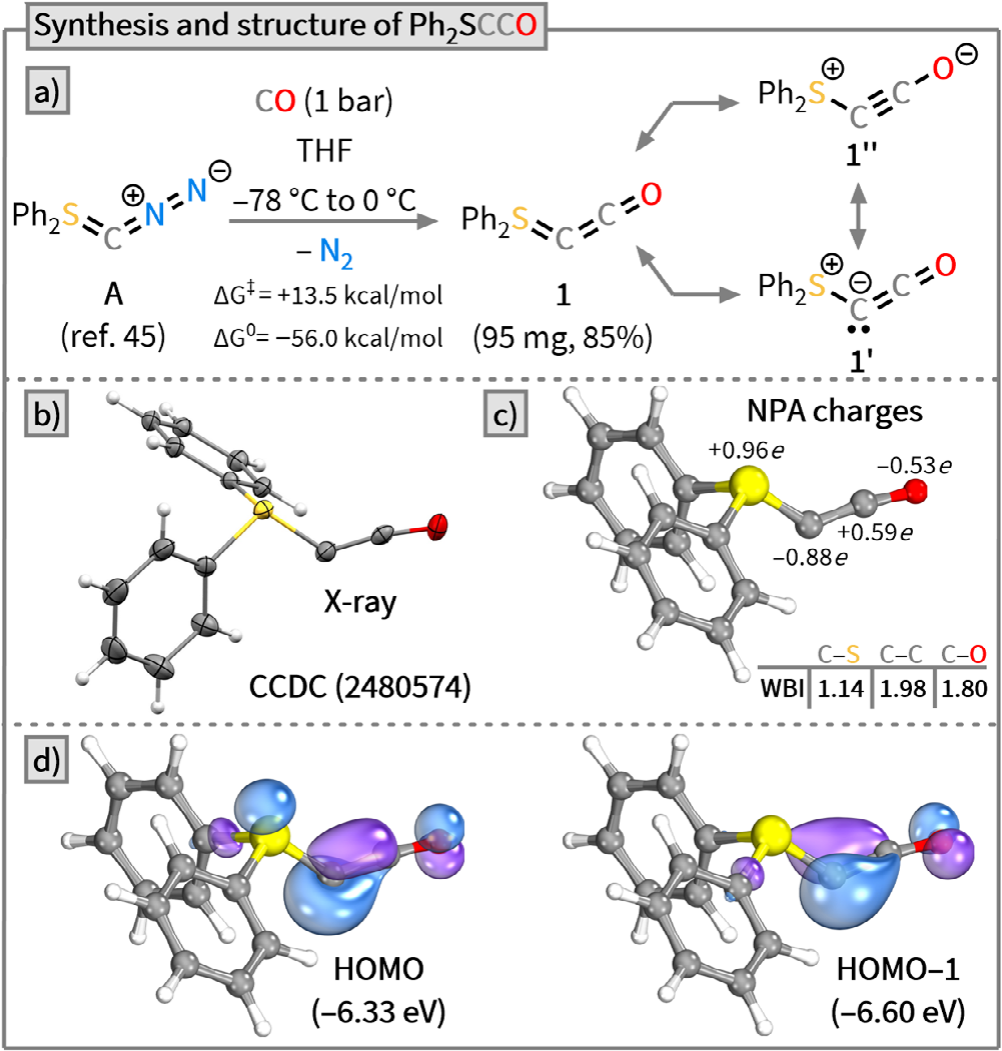
a) Synthesis of heterocumulene **1** and computed Δ*G*⁰ and ΔG^‡^ values for the formal N_2_/CO exchange at the PBE0‐D3(BJ)/def2‐TZVP/SMD(THF) level of theory. b) X‐ray solid‐state structure of **1**. Thermal ellipsoids are shown with 50% probability. c) NPA charges and Wiberg bond indices (WBIs) of **1**. d) Frontier molecular orbitals of **1** (isovalue = 0.6).

Based on differential scanning calorimetry (DSC), cumulene **1** decomposes around 82 °C, similar to the diazo precursor **A**; however, the decomposition enthalpy is much smaller [Δ*H* ∼ 400 J g^−1^ (**1**) versus Δ*H* ∼ 1100 J g^−1^ (**A**)^[^ ^51^ ^]^]. X‐ray diffraction analysis^[^ ^52^ ^]^ of **1** utilizing NoSpherA2^[^ ^53^, ^54^ ^]^ refinement shows a shorter S1─C1 bond [1.6717(13) Å] than in its precursor Ph_2_SCN_2_ [1.727(2) Å]. The C1─C2 bond [1.2767(16) Å] is slightly longer than in Ph_3_PCCO (**VIII**)^[^ ^55^, ^56^ ^]^ [1.247(2) Å]. The S1─C1─C2 angle [125.95(10)°] is wider than in Ph_2_SCN_2_ [112.6(2)°] but significantly narrower than in Ph_3_PCCO [143.1(1)°].^[^ ^55^ ^]^ Considering the structural parameters, ylide‐type structure **1′** likely dominates the overall electronic structure of **1** compared to ylene **1** and ynolate **1′′**.

This interpretation is supported by the unusually high‐field chemical shift of the C1 atom in the ^13^C NMR spectrum at −9.0 ppm (C_6_D_6_). This value is significantly lower than those observed in alkyl‐ or aryl‐substituted ketenes (24–47 ppm)^[^ ^57^ ^]^ or vinylidene ketenes (9–15 ppm),^[^ ^44^, ^47^ ^]^ and is comparable to the C1 atom in **VIII** (−10.5 ppm).^[^ ^58^ ^]^ These values suggest a high electron density at the ylide C‐atom. A natural population analysis of the optimized structure shows that the central C1 atom in **1** carries almost a full negative charge (−0.88e), while the adjacent S‐atom is fully positively charged (+0.96e). The C2 atom and the O‐atom exhibit partial positive (+0.59e) and partial negative (−0.53e) charges, respectively, consistent with a polarized C═O bond.^[^ ^59^ ^]^ Wiberg bond indices (WBIs) indicate that the C─S bond has strong single‐bond character (WBI = 1.14), while the C─C (WBI = 1.98) and C─O bonds (WBI = 1.80) are best described as double bonds, supporting the strong contribution of Lewis structure **1′**. The two highest occupied molecular orbitals (HOMO and HOMO‒1) reveal a picture consistent with other carbone‐type systems.^[^ ^60^, ^61^ ^]^ Both HOMO‒1 (π‐type) and HOMO (σ‐type) show significant lone pair character on C1, with some delocalization onto the adjacent carbonyl fragment. Due to the pronounced localization of electron density at C1, **1** should exhibit nucleophilicity and Brønsted basicity.

With **1** in hand, reactivity studies were carried out to evaluate its suitability as a C_2_ synthon. Following the reactivity design outlined above, **1** was reacted with benzyl alcohol (BnOH, **2a**). In situ NMR monitoring (see Supporting Information) revealed that, after 14 h at room temperature, **1** was fully consumed to give the ester‐based S‐ylide **3a** with high selectivity. Given that S‐ylides exhibit Corey–Chaykovsky‐type reactivity in the presence of Michael acceptors,^[^ ^62^ ^]^ the in situ generated ylide **3a** was subsequently reacted with the alkylidene malonate **4a**. After just 10 min, complete consumption was observed, and chromatographic purification afforded the desired α‐cyclopropyl ester **6aa** in 81% yield (Scheme 3a). This experiment clearly demonstrates that **1** is in principle capable of accurately mimicking the reactivity of C_2_O, while offering a significantly tamed and highly selective reaction profile.

**Scheme 3:**
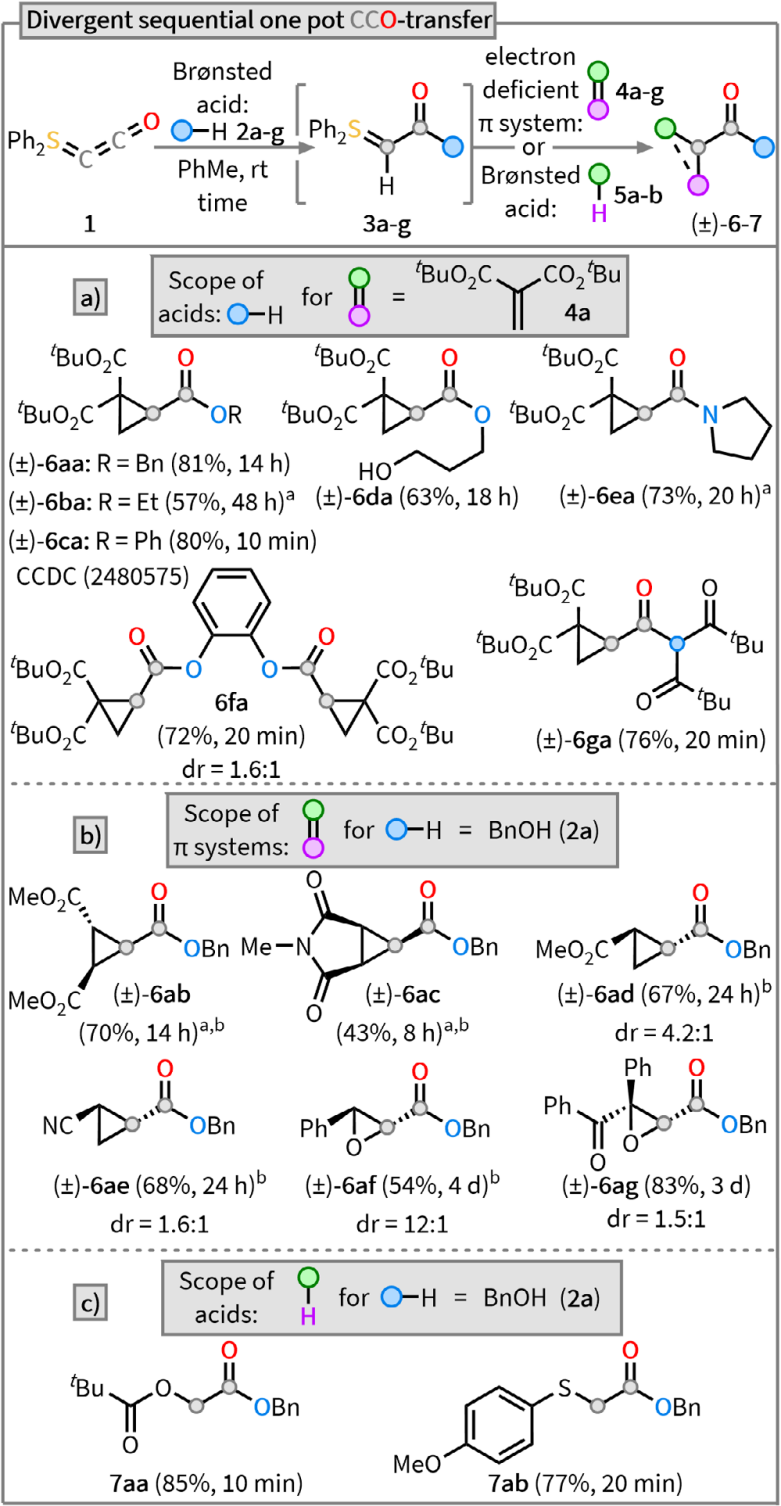
Reactivity studies of **1**. a) Sequential one‐pot CCO transfer using different Brønsted acids. Depicted reaction times refer to the first step. Cyclopropanation (second step) is complete in all cases after 10 min at rt. b) Scope of different electron‐deficient π‐systems. Depicted reaction times refer to the second step at rt. c) Scope of different Brønsted acids. ^a)^Reaction performed in C_6_D_6_. ^b)^Second step was conducted at 60‒100 °C instead of rt, for details, see Supporting Information.

To evaluate the generality of the CCO‐transfer, additional alcohols **2b‐g** were tested using the same Michael acceptor **4a** as terminating reagent for the one‐pot procedure. Counterintuitively, the complete conversion of EtOH (**2b**) to the corresponding ethyl ester‐substituted S‐ylide **3b** required 48 h. The low stability of ylide **3b** at room temperature was already reported in 1966 by Nozaki et al.^[^ ^63^ ^]^ and may explain the moderate yield (57%) of **6ba** observed after prolonged reaction times. In line with the increased acidity (p*K*_a_ ≈ 10) of phenol (PhOH, **2c**) compared to aliphatic alcohols (p*K*_a_ ≈ 16), the formation of the corresponding S‐ylide **3c** proceeded significantly faster, with complete conversion observed after just 10 min. The product **6ca** was isolated in very good yield (80%) and the structure was verified by X‐ray diffraction (see Supporting Information). The use of diols (**2d** and **2f**) also led, depending on the equivalents of **1** used, to either the hydroxy ester **6da** (63%) or the diester **6fa** (72%).

Next, we became interested in whether other acidic compounds could also be exploited for CCO transfer. Surprisingly, the method proved compatible even with nonacceptor‐substituted amines (p*K*_a_ ≈ 35). Sequential reaction of **1** with pyrrolidine **2e** and subsequently with **4a** afforded the α‐cyclopropylamide **6ea** in a good yield of 73%. To our delight, the use of C─H acidic compounds was also possible, as exemplified using dipivaloylmethane **2g**. Following the established one‐pot procedure, three new C─C bonds were formed, and the cyclopropyl ketone **6ga** was obtained in 76% yield.

Once the scope of Brønsted‐acidic substrates had been explored, we turned our attention to investigating whether the in situ generated S‐ylide intermediate **3** could also be reacted with other electron‐deficient olefins (Scheme 3b). For these studies, the benzyl ester‐based S‐ylide **3a** was generated in situ and treated with dimethyl fumarate **4b** which led to formation of the literature‐known 1,2,3‐substituted cyclopropane **6ab** ^[^ ^64^ ^]^ as a single *trans*‐diastereomer in 70% yield. Reaction with the cyclic 1,2‐disubstituted olefin *N*‐methylmaleimide (**4c**) afforded the azabicyclo[3.1.0]hexane derivative **6ac** in moderate yield (43%). The relatively low yield here is attributed to the additional formation of a mixture of the *trans*‐product and undesired cyclopropane ring opening (see Supporting Information). The reaction of S‐ylide **3a** with mono‐acceptor‐substituted olefins such as methyl acrylate (**4d**) and acrylonitrile (**4e**) gave a separable diastereomeric mixture of the disubstituted cyclopropanes **6ad** and **6ae** in good yields of 67% and 68%, respectively, with moderate diastereoselectivity favoring the *trans*‐diastereomer.

S‐ylides are well‐known for their ability to convert aldehydes and ketones into the corresponding epoxides.^[^ ^17^, ^65^ ^]^ To probe this reactivity, we treated **3a** with benzaldehyde (**4f**) or benzil (**4g**) and could isolate the desired epoxides **6af** and **6ag** in moderate (54%) to good (83%) yields, respectively. Finally, we investigated whether **3a** could also undergo a transition metal‐free formal insertion into polarized X─H bonds (Scheme 3c). This type of transformation typically proceeds via a two‐step mechanism involving protonation of the ylide followed by an S_N_‐type substitution.^[^ ^66^ ^]^ Given that highly acidic substrates are ideal for this transformation, we first tested pivalic acid (**5a**) (p*K*_a_ ≈ 5) as a reaction partner. After only 10 min, no ylide could be detected, and subsequent isolation afforded the desired pivalate ester **7aa** in excellent yield (85%). Likewise, treatment with 4‐methoxythiophenol (**5b**) (p*K*_a_ ≈ 7) gave the corresponding thioether **7ab** in 77% yield.

Having successfully demonstrated that **1** can be used both sequentially and divergently to link two different molecules, we explored whether the CCO fragment could also be transferred intramolecularly. Hence, we synthesized substrates **8** containing a Brønsted‐acidic functional group connected via either a C_1_ or C_2_ linker to an acceptor‐substituted olefin. A successful CCO fragment transfer in such systems would thus enable the direct formation of complex annulated ring systems from straightforward, acyclic precursors in a single step.

Tosyl‐substituted allylamine **8a** reacted in just 15 min with **1** at room temperature to afford the bicyclic lactam **10a** in a very good yield of 77% (Scheme 4a). A 1,2‐disubstituted double bond (**8b**) gave product **10b** as a single diastereomer in excellent yield (88%). Higher substituted olefins (**8c** and **8d**) were also well tolerated, affording the corresponding substituted cyclopropanes with high diastereoselectivity and excellent yields (**10c**: 87%; **10d**: 76%). The introduction of alkyl substituents on the Michael acceptor typically results in reduced reactivity, which likely explains why CCO transfer in these cases required elevated temperatures (60 °C) and extended reaction times (12 h). Notably, the reaction is highly diastereoselective but not diastereospecific, yielding a single diastereomer from both **8c** and **8d**, irrespective of the configuration of the starting alkene *E*/*Z* double bond configuration. This observation supports a stepwise cyclopropanation mechanism, in which the same betaine‐like intermediate is formed.^[^ ^67^ ^]^ Notably, the CCO transfer approach also enables the diastereoselective construction of synthetically challenging quaternary stereocenters, as demonstrated in the case of **10c**.

**Scheme 4:**
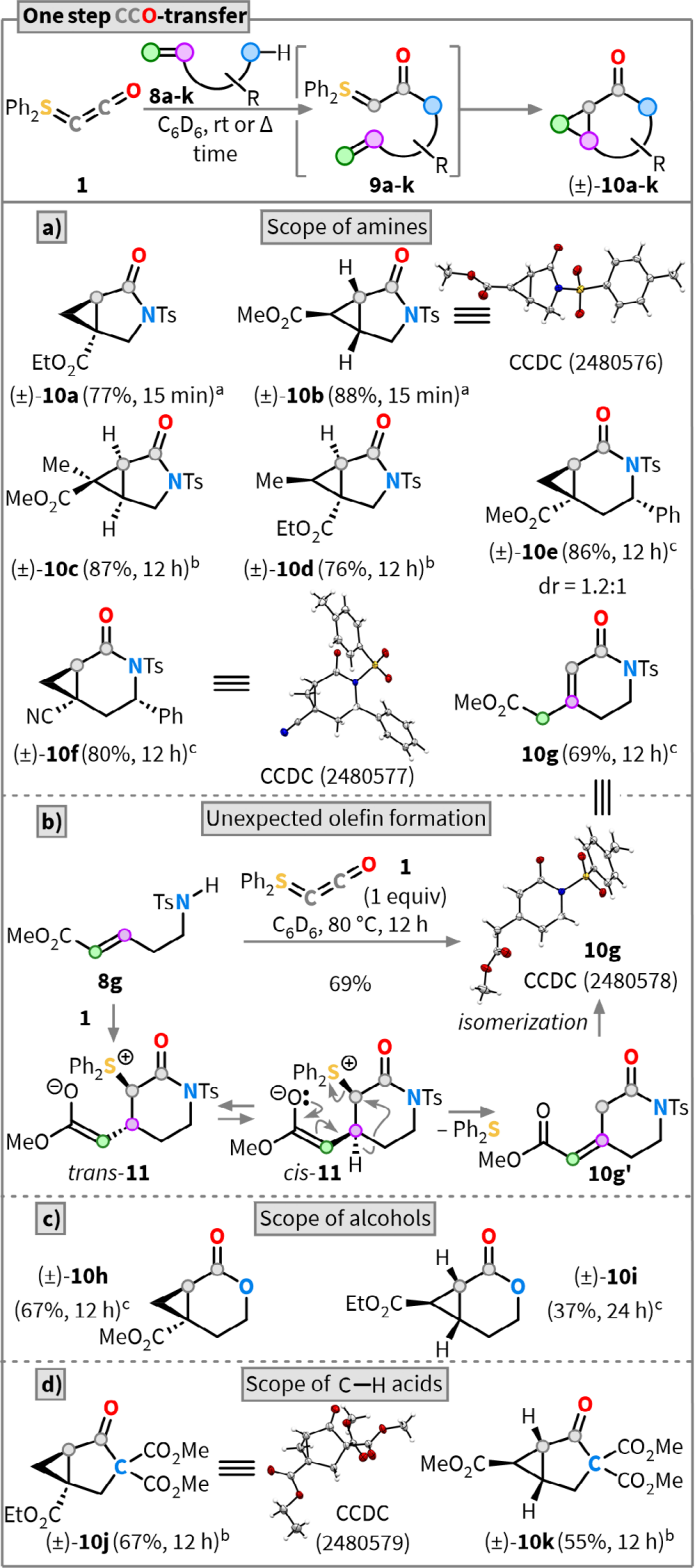
Reactivity studies of **1** in a one‐step CCO‐transfer. a) Scope of different amines. b) Mechanistic proposal for the formation of the unexpected olefin **10g**. c) Scope of different alcohols. d) Scope of different C‒H acids. ^a)^Reaction was performed at rt. ^b)^Reaction was first stirred at rt and then heated to 60 °C or 80 °C, for details, see Supporting Information. ^c)^Reaction conducted at 80 °C.

Extension of the linker length with Tosyl‐substituted homoallylamines (**8e** and **8f**) resulted in the corresponding azabicyclo[4.1.0]heptanones **10e** and **10f** in excellent yields of 86% and 80%, respectively. Surprisingly, the use of 1,2‐disubstituted olefin **8g** did not yield the expected bicyclic product but gave the unsaturated lactam **10g** in 69% yield. We postulate that the formation of this undesired constitutional isomer proceeds via initial formation of the *trans*‐configured betaine (*trans*‐**11**), which exists in equilibrium with *cis*‐**11** (Scheme 4b).^[^ ^68^ ^]^ Since the stereoelectronic features of *trans*‐**11** are not favorable for a fast intramolecular S_N_2 reaction, a competing semipinacol‐type rearrangement starting from *cis*‐**11** and releasing Ph_2_S to form **10g’** is postulated. **10g’** isomerizes into the observed unsaturated lactam **10g** as the final product, verified by X‐ray diffraction.

We next investigated the influence of the Brønsted‐acidic functionality. Analogous to the nitrogen‐based substrate **8e**, the corresponding oxygen analogue **8h** reacted to afford the bicyclic lactone **10h** in a satisfactory yield of 67% (Scheme 4c). Prompted by the unusual reactivity of amine **8g**, we turned our attention to the corresponding oxygen analogue **8i**. Similar challenges in achieving the desired CCO transfer were encountered, with the product **10i** being isolated in only 37% yield together with other unidentified byproducts. Finally, we turned our attention to exploring the construction of purely carbocyclic frameworks using **8j**. Remarkably, triester **8j** reacted with **1** after 12 h at 60 °C, to give the desired bicyclo[3.1.0]hexane **10j** as a crystalline solid in 67% yield. Similarly, the use of the 1,2‐disubstituted olefin **8k** led to the formation of the bicyclic product **10k** in a satisfactory yield of 55%.

In summary, we have reported the synthesis, isolation, and characterization of the unprecedented heterocumulene Ph_2_S═C═C═O (**1**). We have shown that **1** mimics the reactivity of the nonisolable dicarbon monoxide (C_2_O), enabling for the first time a highly selective CCO transfer across a broad substrate scope. This reactivity allows for the transition metal‐free one‐step synthesis of highly complex 3D building blocks from simple starting materials. Considering the broad use of Bestmann ylide in organic synthesis, we are confident that the S‐ylide based reagent **1** will find wide application as a versatile C_2_ synthon in synthesis.

## Supporting Information

The authors have cited additional references within the Supporting Information.

## Conflict of Interests

A patent application related to the CCO transfer reagent was filed.

## Supporting information



Supporting Information

Supporting Information

## Data Availability

The data that support the findings of this study are available in the Supporting Information of this article.
