# Supplementary material for: Ph2SCCO: A New Versatile CCO‐Fragment Transfer Reagent
Source: Angew Chem Int Ed Engl. 2025 Oct 10;64(47):e202518689. doi: 10.1002/anie.202518689 (PMC12624311; doi:10.1002/anie.202518689)
Supplement: Supplementary file 1 — Supporting Information [file ANIE-64-e202518689-s001.pdf]

Ph_2SCCO: A New Versatile CCO-Fragment Transfer Reagent

Table of Contents

1. General Information.. S2

2. Experimental and analytical details.. S3

3. X-ray crystallography.. S74

4. Computational details.. S80

5. References.. S87

General Information

The reaction and hanlding of air-sensitive compounds were carried out under N_2 atmosphere using a high-vacuum line, standard Schlenk techniques, or a glovebox, as well as dry and oxygen-free solvents. ^{1}H, ^{13}C and ^{31}P nuclear magnetic resonance (NMR) spectra were recorded on a Bruker AV 500 Avance NEO, Bruker AV 400 Avance III HD NanoBay, AV 600 Avance III HD and AV 700 Avance III HD spectrometer at 298 K unless otherwise noted. NMR chemical shifts (δ) in C_6D_6, $CDCl_3$, CD_2Cl_2, THF-*d*$_8$, C_6D_5Br or CD_3CN are referenced to their solvent signals [C_6D_6, 7.16 (^{1}H NMR), 128.06 (^{13}C NMR); CD_3CN, 1.94 (^{1}H NMR), 118.26 (^{13}C NMR), $CDCl_3$ 7.26 (^{1}H NMR), 77.16 (^{13}C NMR), CD_2Cl_2, 5.32 (^{1}H NMR), 53.84 (^{13}C NMR), THF-*d*$_8$, 3.58 (^{1}H NMR), 67.21 (^{13}C NMR), C_6D_5Br, 6.94 (^{1}H NMR), 122.35 (^{13}C NMR)]. ^{31}P NMR chemical shifts are relative to 85% aqueous H_3PO_4 in ppm. Data are reported as follows: chemical shift, multiplicity (s = singlet, d = doublet, t = triplet, sep = septet, brs = broad singlet, brd = broad doublet, m = multiplet), coupling constant in Hertz (Hz), and an integration value. High resolution MS (EI): Finnigan MAT 8200 (70 eV), ESIMS: Finnigan MAT 95, accurate mass determinations: Bruker APEX III FT-MS (7 T magnet) and LTQ-Orbitrap-XL (Thermo Scientific) equipped with a heated electrospray ionization source (HESI). Flash chromatography was performed with Merck 60 silica gel (40-63 µm). Thin-layer chromatography (TLC) analysis was performed using Merck silica gel 60 F254 TLC plates and visualized by UV irradiation and/or ceric ammonium molybdate, $KMnO_4$ or *p*-anisaldehyde. The reactions were performed under dry argon or nitrogen atmosphere unless otherwise noted. IR-ATR measurements (diamond) were performed in reflection mode on a Bruker Alpha II inside a glovebox, wavenumbers in cm^{-1}. Melting points were measured with a Büchi M-560 apparatus. N_2O was directly used from the gas bottle (quality 5.0 obtained from Messer).

Materials: compound **A**[1], **8a**[4], **8b**[5], **8c**[6], **8d**[7], **8e**[8], **8f**[8], **8g**[5], **8h**[9], **8i**[10], **8j**[11], **8k**[12] were prepared according to the literature procedures. All other reagents were commercially available and used as recieved.

Experimental and Analytical Details

Synthesis of compound 1

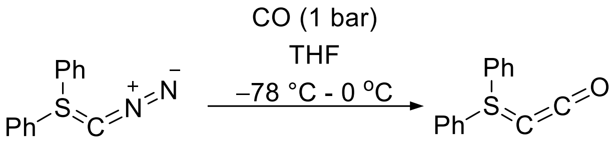

A **1**

A solution of compound **A** (111.5 mg, 0.5 mmol) was dissolved in THF (10 mL) and degassed by freeze-pump-thaw cycles twice at -78 °C. Then the mixture was exposed to a CO atmosphere (1.0 bar) at -78 ºC. The reaction mixture was gradually raised to 0 ºC and stirring was continued for 3 hours at this temperature. (Caution: the actual pressure in the flask at room temperature is higher; use pressure glassware and an explosion shield). The volatiles were removed to a solution volume of ca. 1 mL. *n*-Pentane (10 mL) was added into the solution with continuous stirring, during this time a colorless solid formed, which was collected by filtration and washed with cold *n*-pentane (3×1 mL) to give the desired diphenylsulfur ylide ketene **1** (94.8 mg, 0.42 mmol, 85%). Crystals of diphenylsulfur ylide ketene **1** suitable for X-ray diffraction were obtained from a solution of the colorless solid in THF and *n*-pentane (ratio: 1:2) at -40 ºC.

Characterization data of compound **1**:
m.p. 82 ºC (decomposition).
^{1}H NMR (500 MHz, 298 K, C_6D_6): δ = 7.43 (m, *o*-Ph, 4H), 6.80 (m, *m*, *p*-Ph, 6H).
^{13}C{^{1}H} NMR (125 MHz, 298 K, C_6D_6): δ = 151.7 (C=O), 139.8 (*i*-Ph), 131.0 (*p*-Ph), 129.6 (*m*-Ph), 126.6 (*o*-Ph), -9.0 (S=C).
HR-MS-ESI(+) calc. $C_{14}H_{11}OS^+$ $[M+H]^+$ 227.0525, found 227.0527.
IR (ATR) [cm^{-1}]: ṽ = 3052, 2048, 1628, 1578, 1473, 1443, 1304, 1225, 1174, 1065, 1021, 998, 740, 685, 548, 513, 472.

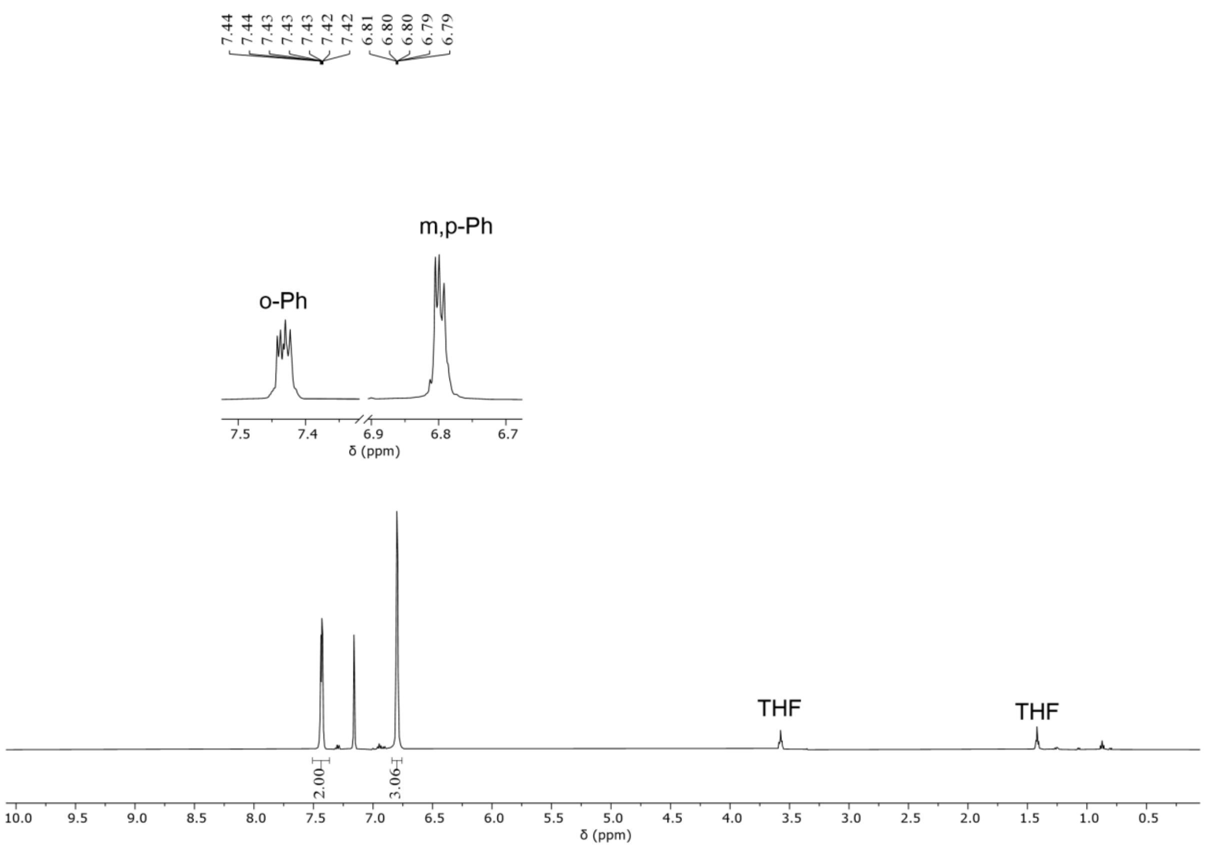

Figure S1. ^{1}H NMR (500 MHz, 298 K, C_6D_6) spectrum of compound **1** [admixed with THF].

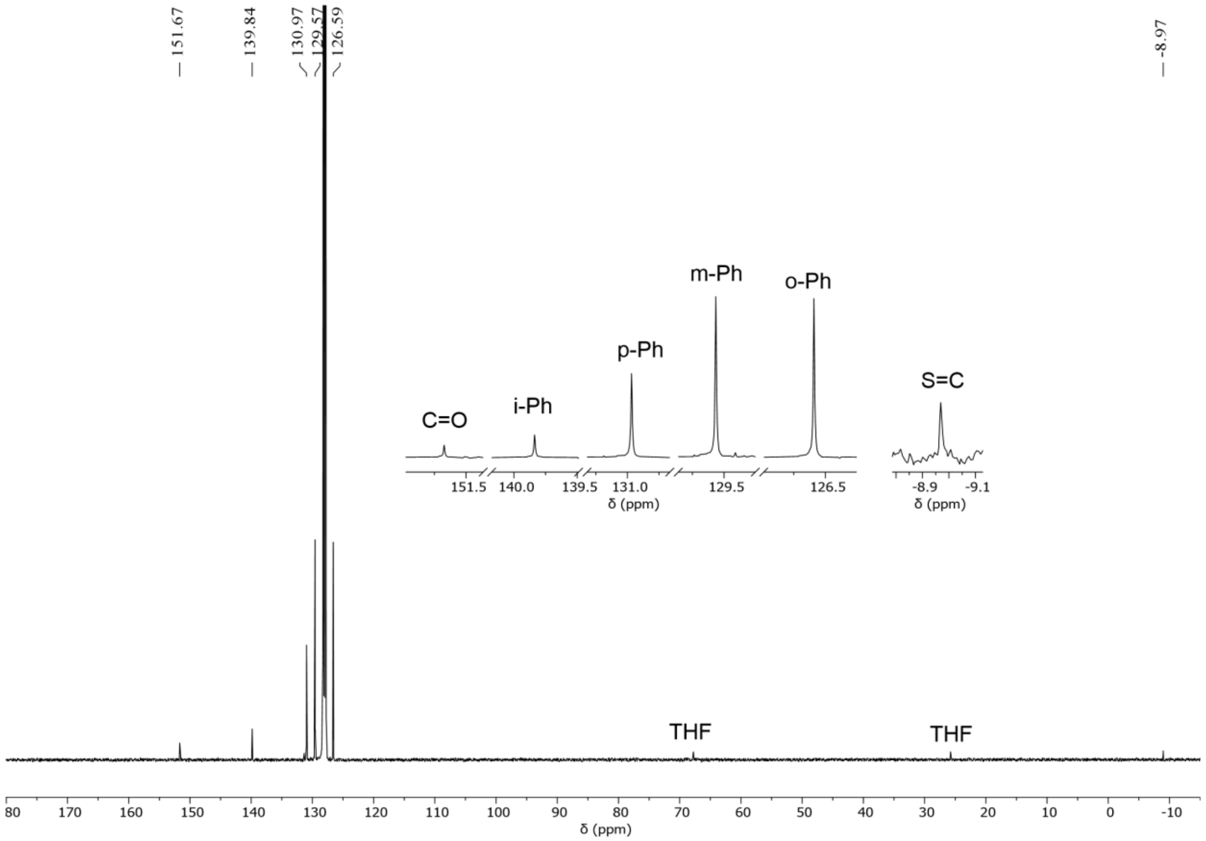

Figure S2. $^{13}C\{^{1}H\}$ NMR (125 MHz, 298 K, C_6D_6) spectrum of compound **1** [admixed with THF].

TGA and DSC measurements for compound 1

Thermogravimetric analysis measurements were performed on a Discovery SDT 650 instrument from TA Instruments under a constant nitrogen flow of 100 mL/min. For the measurement a small amount (~ 3 mg) of the powdered sample was placed in a 90 µL alumina crucible and heated from 40 °C to 200 °C with a constant heating rate of 10 K/min.

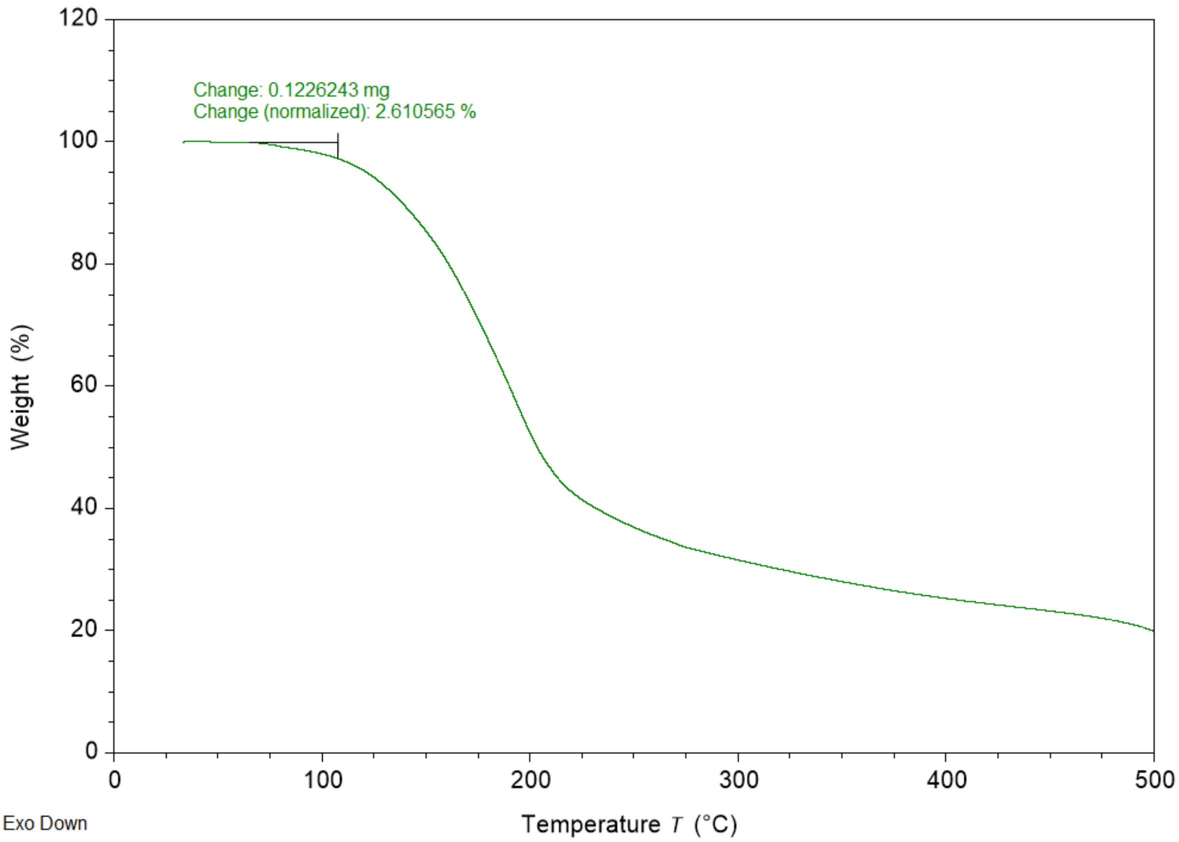

Figure S3. Thermogravimetric analysis of compound **1**.

The stability of compound **1** was determined by differential scanning calorimetry (DSC) on a TA Discovery DSC25 machine. Samples (2.70-3.00 mg) were sealed in pressure resistant crucibles (TA DSC high pressure gold plated capsule seals and high-pressure stainless-steel capsule). The crucibles were calibrated against an indium reference and were heated in an oven from 30°C to 210°C at a rate of 5°C/min. The heat flow was monitored vs. an empty, nitrogen filled reference crucible. A single highly exergonic (ΔH_D > 362 J/g) decomposition with a very sharp DSC peak (see Fig. S4 and S5). The DSC behavior was reproducible with two times measurements.

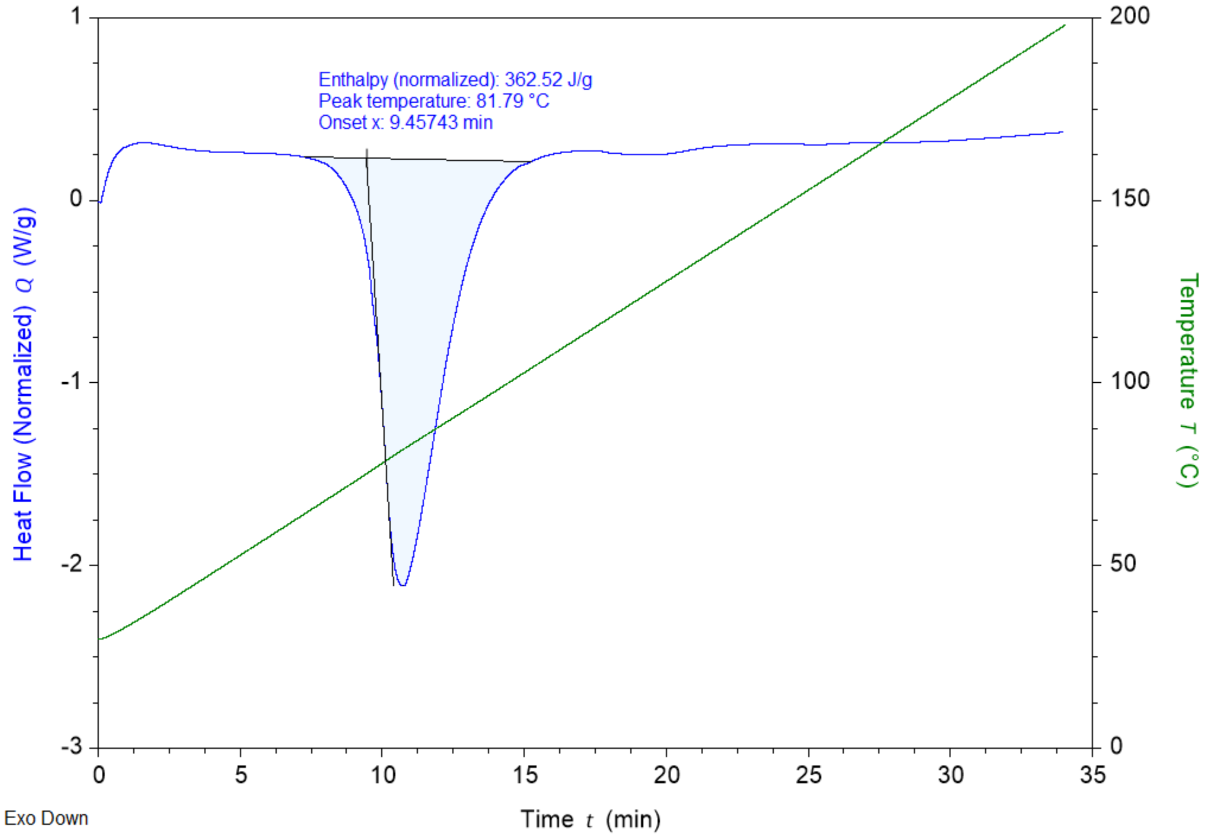

Figure S4. DSC measurement of compound **1** (2.70 mg).

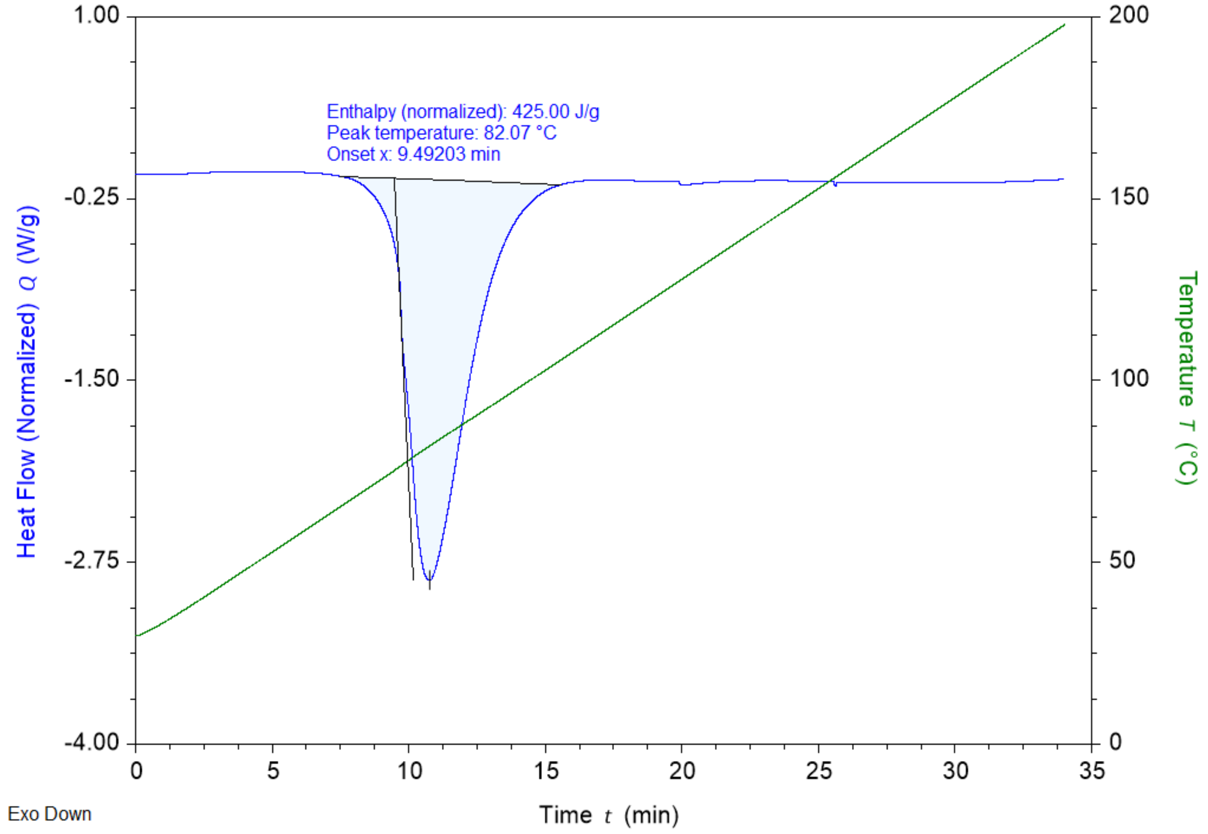

Figure S5. DSC measurement of compound **1** (3.00 mg).

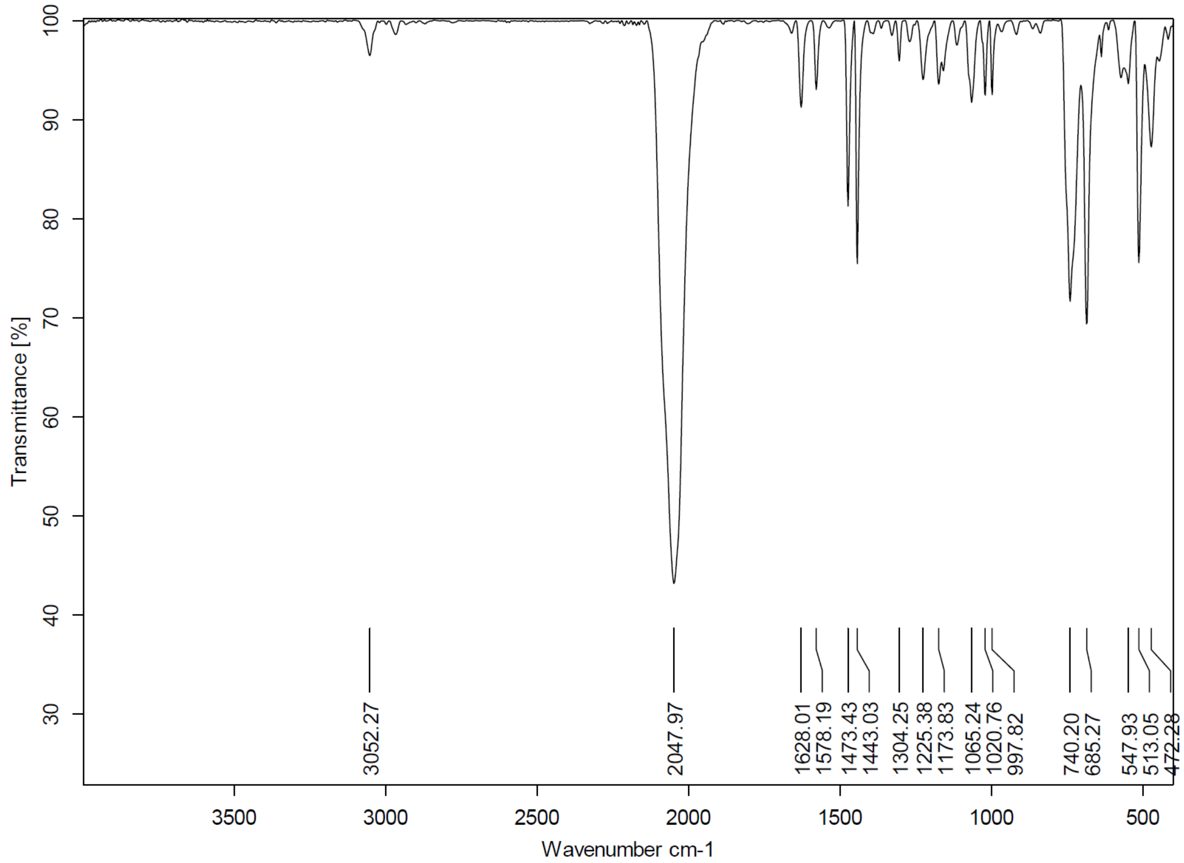

Figure S6. ATR-IR spectrum of compound **1**.

Synthesis of compound (±)-6aa

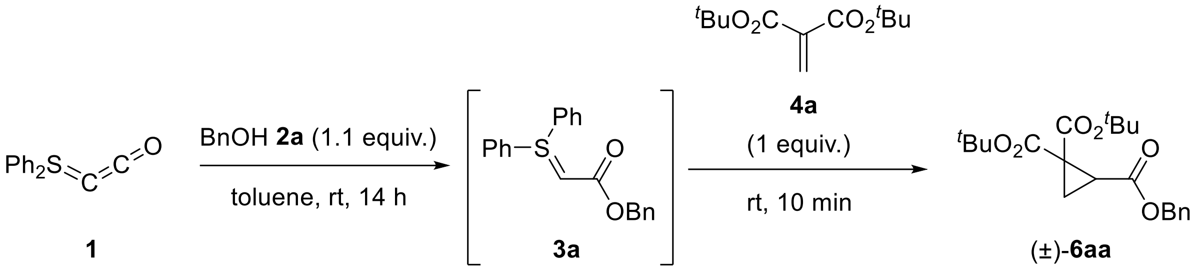

Reagent **1** (67.89 mg, 0.3 mmol, 1 equiv.) was dissolved in toluene (3 mL). Benzyl alcohol **2a** (35.69 mg, 0.33 mmol, 1.1 equiv.) was added and the reaction was stirred at room temperature. After 14 hours compound **4a** (68.49 mg, 0.3 mmol, 1 equiv.) was added and the reaction was stirred for further 10 minutes at room temperature. Purification of the reaction mixture by silica gel column chromatography (ethyl acetate in pentane: 0% to 2%) afforded the desired product (±)-**6aa** (91 mg, 0.24 mmol, 81%) as a yellow solid.

Characterization data of compound (±)-**6aa**:

m.p.: 74 °C.
^{1}H NMR (500 MHz, 298 K, C_6D_6): δ = 7.14 (m, 2H, Ph) 7.06 (m, 3H, Ph) 5.01 (d, $^2J_{HH}$ = 1H, O-CH_2-Ph) 4.94 (d, $^2J_{HH}$ = 1H, O-CH_2-Ph) 2.70 (dd, $^3J_{HH}$ = 6.7 Hz, $^3J_{HH}$ = 8.6 Hz, 1H, CH) 2.00 (dd, $^2J_{HH}$ = 4.4 Hz, $^3J_{HH}$ = 6.7 Hz, 1H, CH_2) 1.45 (s, 9H, ^{t}Bu) 1.38 (dd, $^2J_{HH}$ = 4.4 Hz, $^3J_{HH}$ = 8.5 Hz, 1H, CH_2) 1.25 (s, 9H, ^{t}Bu).
^{13}C{^{1}H} NMR (125 MHz, 298 K, C_6D_6): δ = 169.8 ($CO_2{}^t$Bu) 168.3 (CO_2Bn) 164.8 ($CO_2{}^t$Bu) 136.3 (Ph-C) 128.7 (2x Ph-CH) 128.7 (2x Ph-CH) 128.3 (Ph-CH) 82.3 (^{t}Bu) 81.2 (^{t}Bu) 67.0 (O-CH_2-Ph) 39.4 ($C(CO_2{}^t Bu)_2$) 28.0 (^{t}Bu) 27.8 (^{t}Bu) 27.6 (CH) 19.7 (CH_2).
IR (ATR) ṽ [cm^{-1}] 1976, 1753, 1728, 1455, 1398, 1368, 1324, 1284, 1257, 1224, 1187, 1152, 1123, 1066, 1022, 957, 929, 646, 838, 801, 760, 742, 698, 682, 577, 536, 497, 467.
HR-MS-ESI (+) calc. $C_{21}H_{28}O_6Na^+$ $[M+Na]^+$ 399.1778; found 399.1778.

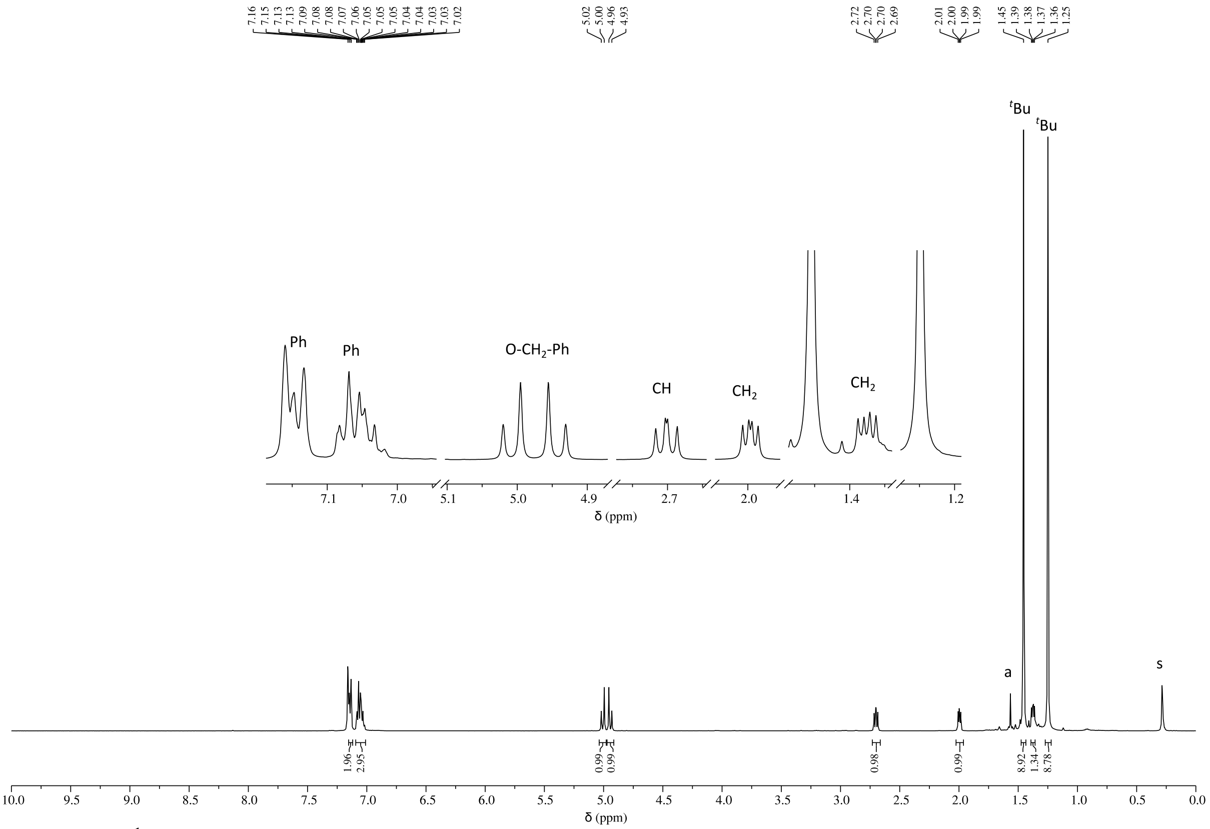

Figure S7. ^{1}H NMR (500 MHz, 298 K, C_6D_6) spectrum of compound (±)-**6aa**. [a: acetone; s: silica grease]

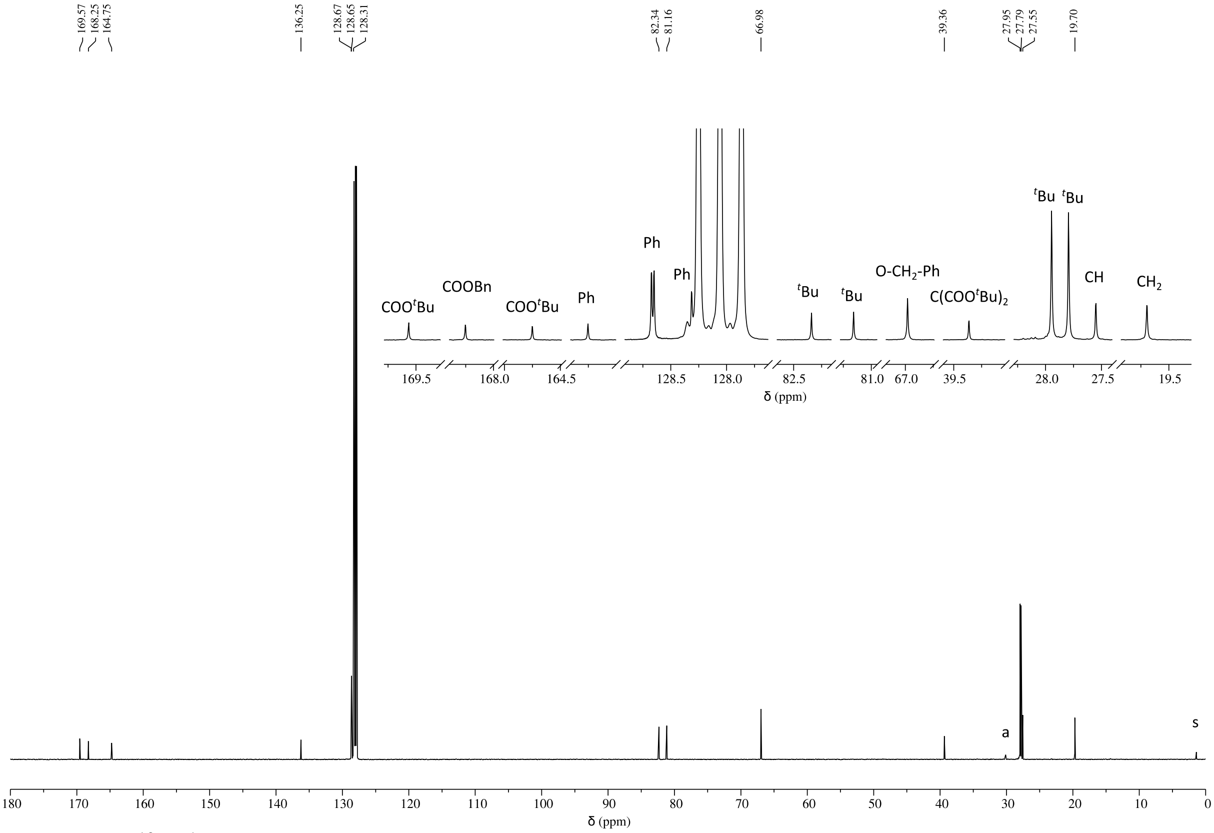

Figure S8. ^{13}C{^{1}H} NMR (125 MHz, 298 K, C_6D_6) spectrum of compound (±)-**6aa**. [a: acetone; s: silica grease]

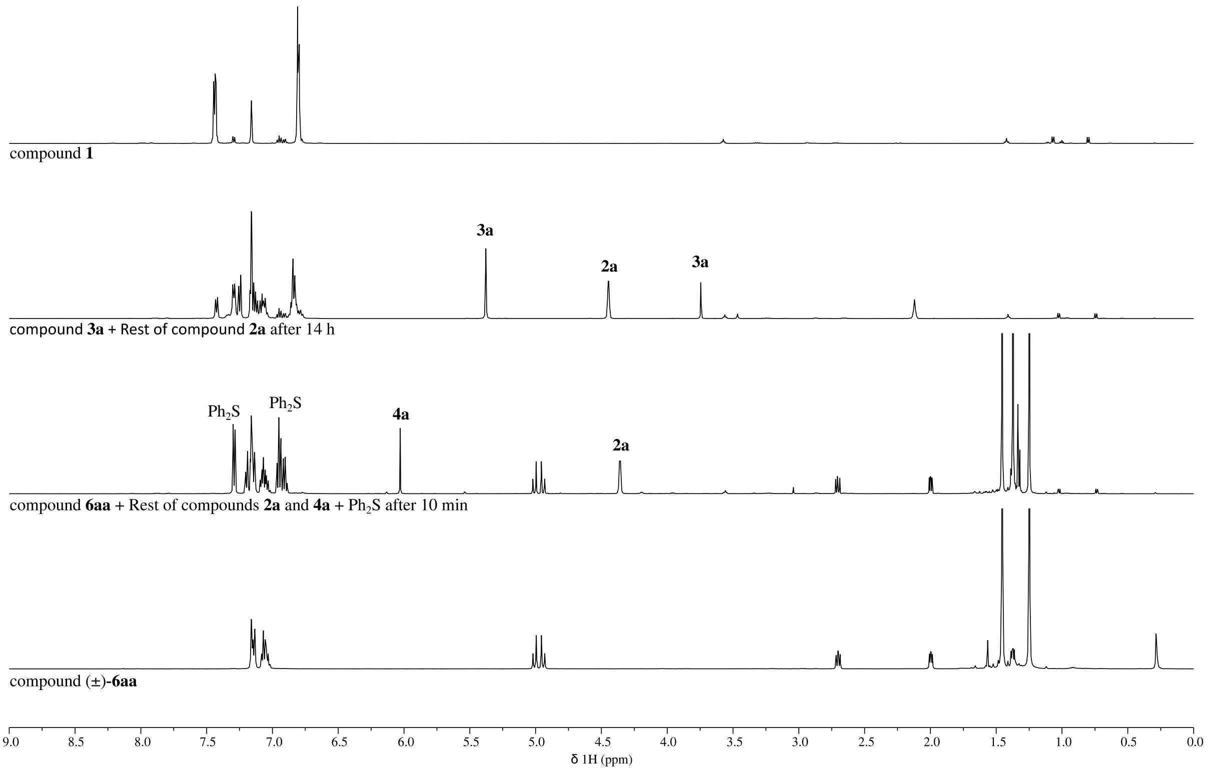

Figure S9. ^{1}H NMR spectra (500 MHz, 298 K, C_6D_6) of the in-situ reaction for the formation of (±)-**6aa**. ^{1}H-NMR spectra (from top to bottom) of compound **1**; 14 h after addition of compound **2a** to form compound **3a**; 10 min after addition of compound **4a** to form product (±)-**6aa**; compound (±)-**6aa** after purification.

Synthesis of compound (±)-6ba

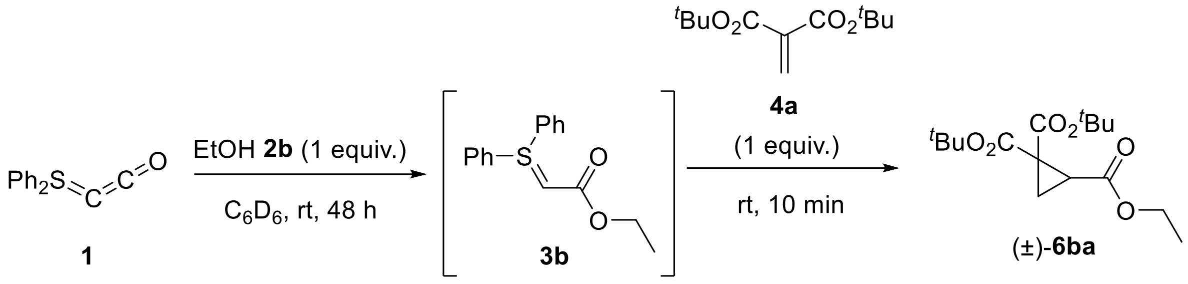

Reagent **1** (23.08 mg, 101.99 µmol, 1 equiv.) was dissolved in C_6D_6 (0.5 ml) and ethanol **2b** (4.70 mg, 0.768 g/ml, 5.96 µl, 101.99 µmol, 1 equiv.) was added. After 48 hours all of compound **1** reacted to the sulfur ylide **3b**. Then, compound **4a** (23.28 mg, 101.99 µmol, 1 equiv.) was added. The reaction showed full conversion after 10 minutes and the reaction mixture was purified by silica gel column chromatography (ethyl acetate in cyclohexane: 0% to 2% to 4% to 6%). The product (±)-**6ba** (18.2 mg, 57.89 µmol, 57%) was obtained as a colourless oil.

Characterization data of compound (±)-**6ba**:

^{1}H NMR (600 MHz, 298 K, C_6D_6): δ = 3.98 (m, 1H, CH_2CH$_3$) 3.87 (m, 1H, CH_2CH$_3$) 2.68 (dd, $^3J_{HH}$ = 6.7 Hz, $^3J_{HH}$ = 8.6 Hz, 1H, CH) 2.02 (dd, $^2J_{HH}$ = 4.3 Hz, $^3J_{HH}$ = 6.7 Hz, 1H, CH$_2$) 1.49 (s, 9H, ^{t}Bu) 1.40 (dd, $^2J_{HH}$ = 4.3 Hz, $^3J_{HH}$ = 8.6 Hz, 1H, CH$_2$) 1.26 (s, 9H, ^{t}Bu) 0.92 (t, $^3J_{HH}$ = 7.1 Hz, 3H, CH$_2$$CH_3$).
^{13}C{^{1}H} NMR (150 MHz, 298 K, C_6D_6): δ = 169.6 (CO$_2$Et) 168.4 (CO$_2$^{t}Bu) 164.8 (CO$_2$^{t}Bu) 82.3 (^{t}Bu) 81.1 (^{t}Bu) 61.1 (CH_2CH$_3$) 39.2 (C) 28.0 (^{t}Bu) 27.8 (^{t}Bu) 27.7 (CH-CO$_2$Et) 19.5 (CH$_2$) 14.2 (CH$_2$$CH_3$).
IR (ATR) ṽ [cm^{-1}] 2979, 2935, 1722, 1478, 1458, 1393, 1368, 1332, 1285, 1257.49, 1227, 1189, 1156, 1126, 1097, 1062, 1036, 991, 839, 816, 757, 740, 467
HR-MS-ESI (+) calc. $C_{16}H_{26}O_6Na^+$ [M+Na]$^+$ 337.1622; found 327.1620.

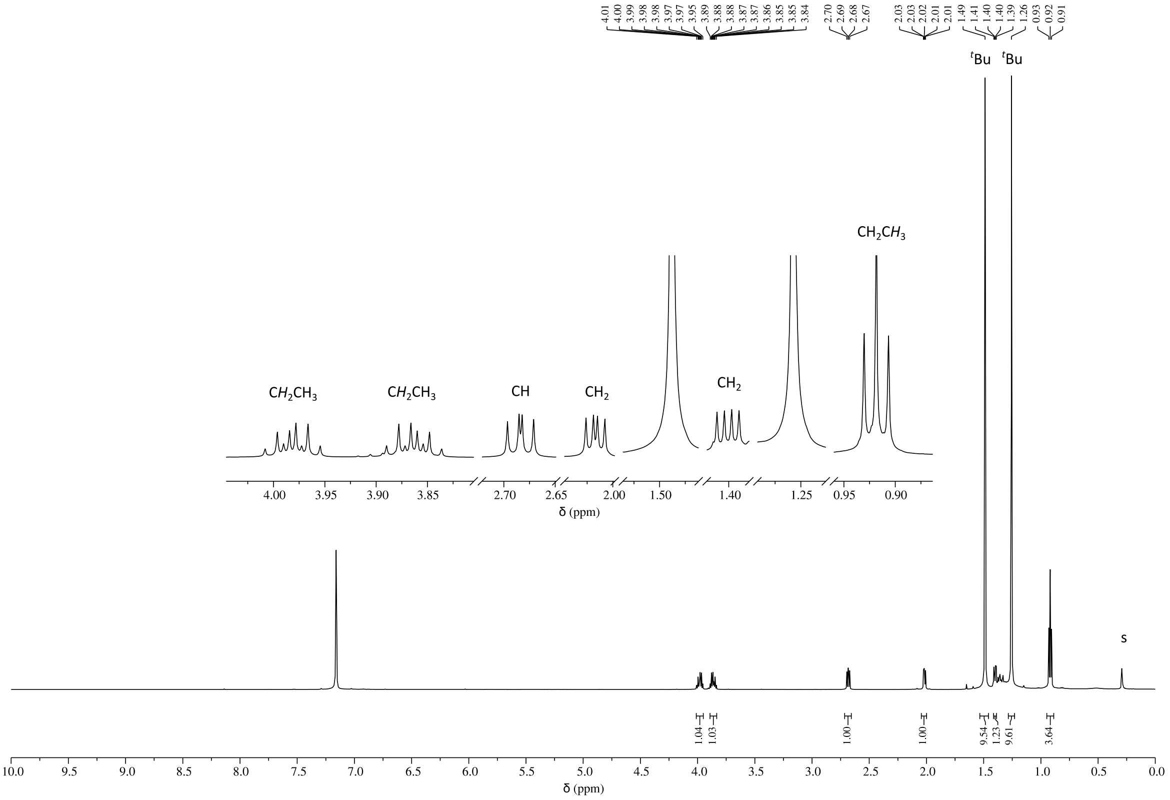

Figure S10.^{1}H NMR (600 MHz, 298 K, C_6D_6) spectrum of compound (±)-**6ba**. [s: silica grease]

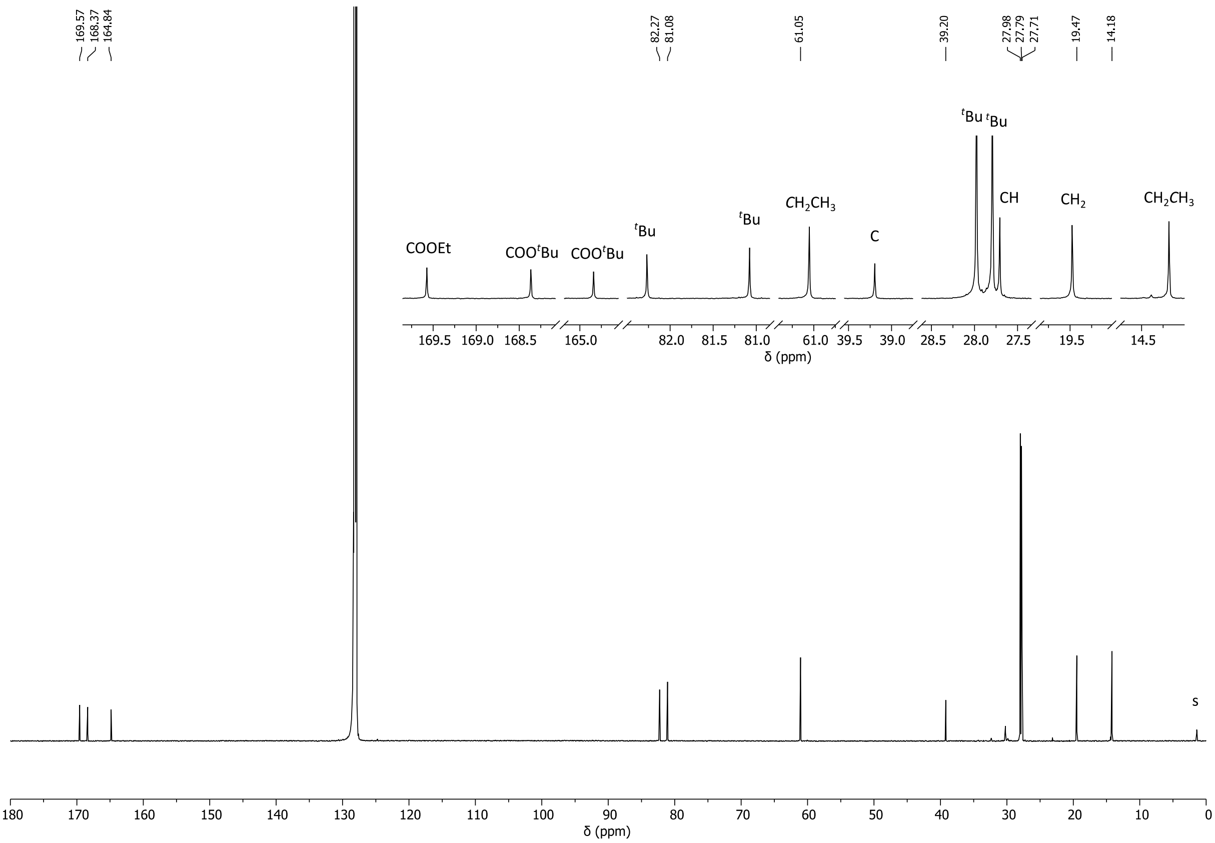

Figure S11.$^{13}C\{^1H\}$ NMR (150 MHz, 298 K, C_6D_6) spectrum of compound (±)-**6ba**. [s: silica grease]

Synthesis of compound (±)-6ca

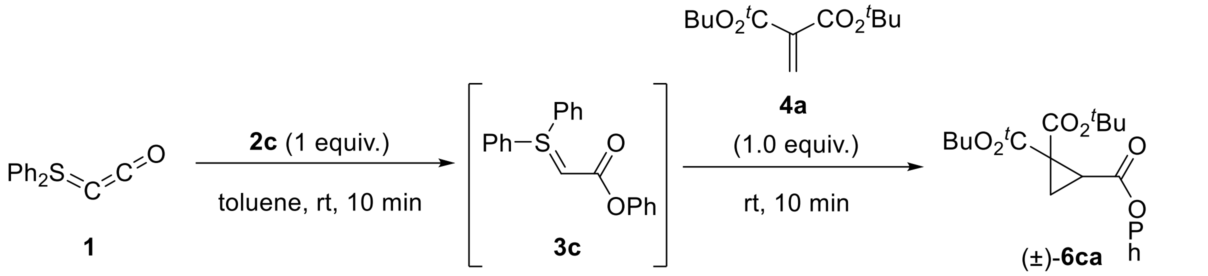

Reagent **1** (90.9 mg, 0.4 mmol, 1.0 equiv.) was dissolved in toluene (3 ml) and phenol **2c** (37.81 mg, 0.4 mmol, 1 equiv.) was added. After 10 minutes stirring at room temperature, compound **4a** (91.71 mg, 0.4 mmol, 1 equiv.) was added and stirred for further 10 min at room temperature. The reaction mixture was purified by silica gel column chromatography (ethyl acetate in pentane: 0% to 2% to 5%) to obtain the product (±)-**6ca** (116 mg, 0.32 mmol, 80%) as a yellow solid. Crystals of compound (±)-**6ca** suitable for X-ray diffraction were obtained from a *n*-pentane solution at 2 °C.

Characterization data of compound (±)-**6ca:**

m.p.: 89 °C.
^{1}H NMR (500 MHz, 298 K, C_6D_6): δ = 7.12 (m, 2H, *o*-Ph-CH) 7.01 (m, 2H, *m*-Ph-CH) 6.86 (m, 1H, *p*-Ph-CH) 2.86 (dd, $^3J_{HH}$ = 6.71, $^3J_{HH}$ = 8.54 Hz, 1H, CH) 2.05 (dd, $^2J_{HH}$ = 4.45, $^3J_{HH}$ = 6.71 Hz, 1H, CH_2) 1.47 (dd, $^2J_{HH}$ = 4.48, $^3J_{HH}$ = 8.55 Hz, 1H, CH_2) 1.43 (s, 9H, ^{t}Bu) 1.28 (s, 9H, ^{t}Bu).
^{13}C{^{1}H} NMR (125 MHz, 298 K, C_6D_6): δ = 168.2 ($CO_2{}^t$Bu) 168.1 ($CO_2{}^t$Bu) 164.7 (CO_2Ph) 151.5 (Ph-C) 129.5 (2x Ph-CH) 125.9 (Ph-CH) 121.9 (2x Ph-CH) 82.6 (^{t}Bu) 81.6 (^{t}Bu) 39.8 (C) 28.0 (^{t}Bu) 27.8 (^{t}Bu) 27.6 (CH) 19.8 (CH_2).
IR (ATR) ṽ [cm^{-1}] 2979, 1752, 1724, 1497, 1480, 1458, 1388, 1369, 1334, 1282, 1258, 1224, 1197, 1173, 1132, 1061, 1024, 1009, 988, 926, 845, 808, 763, 735, 703, 691, 526, 499, 470.
HR-MS-ESI (+) calc. $C_{20}H_{26}O_6Na^+$ $[M+Na]^+$ 385.1622; found 385.1616.

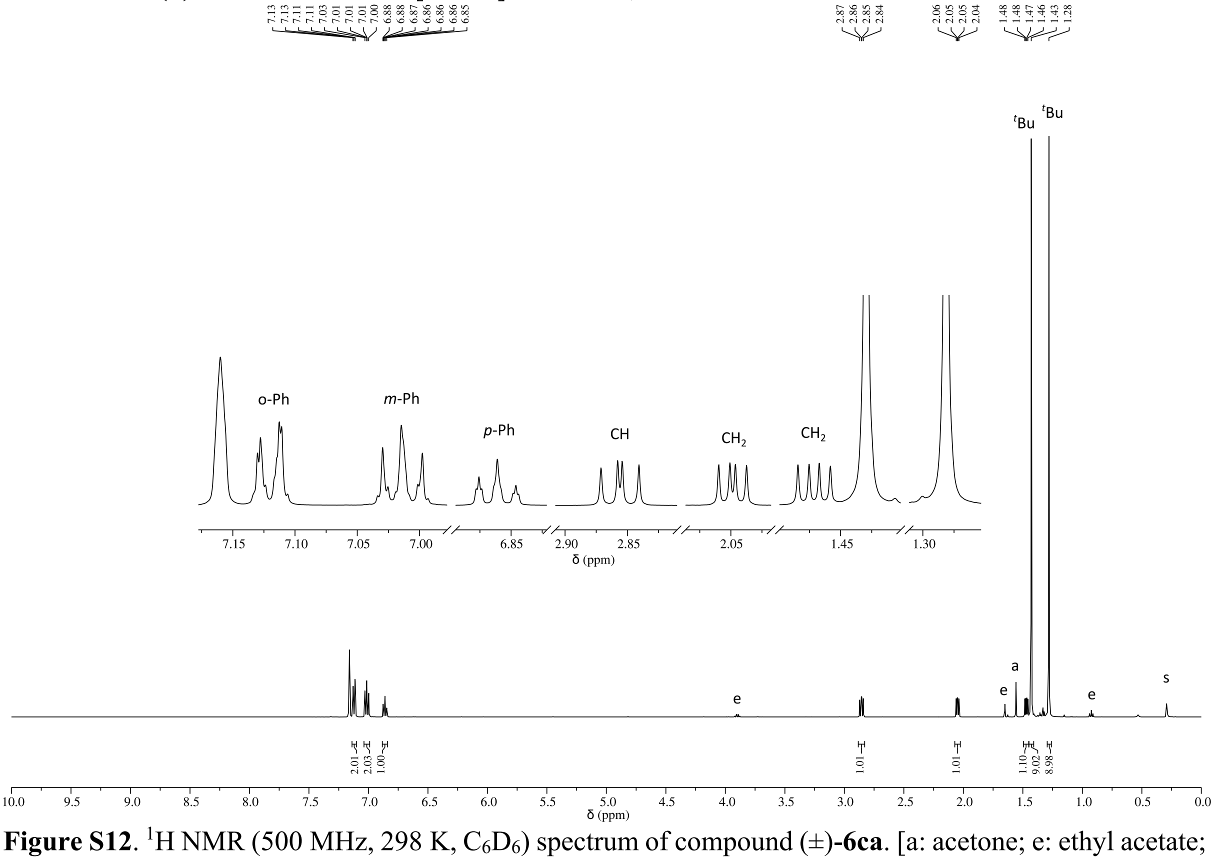

Figure S12. ^{1}H NMR (500 MHz, 298 K, C_6D_6) spectrum of compound (±)-**6ca**. [a: acetone; e: ethyl acetate; s: silica grease]

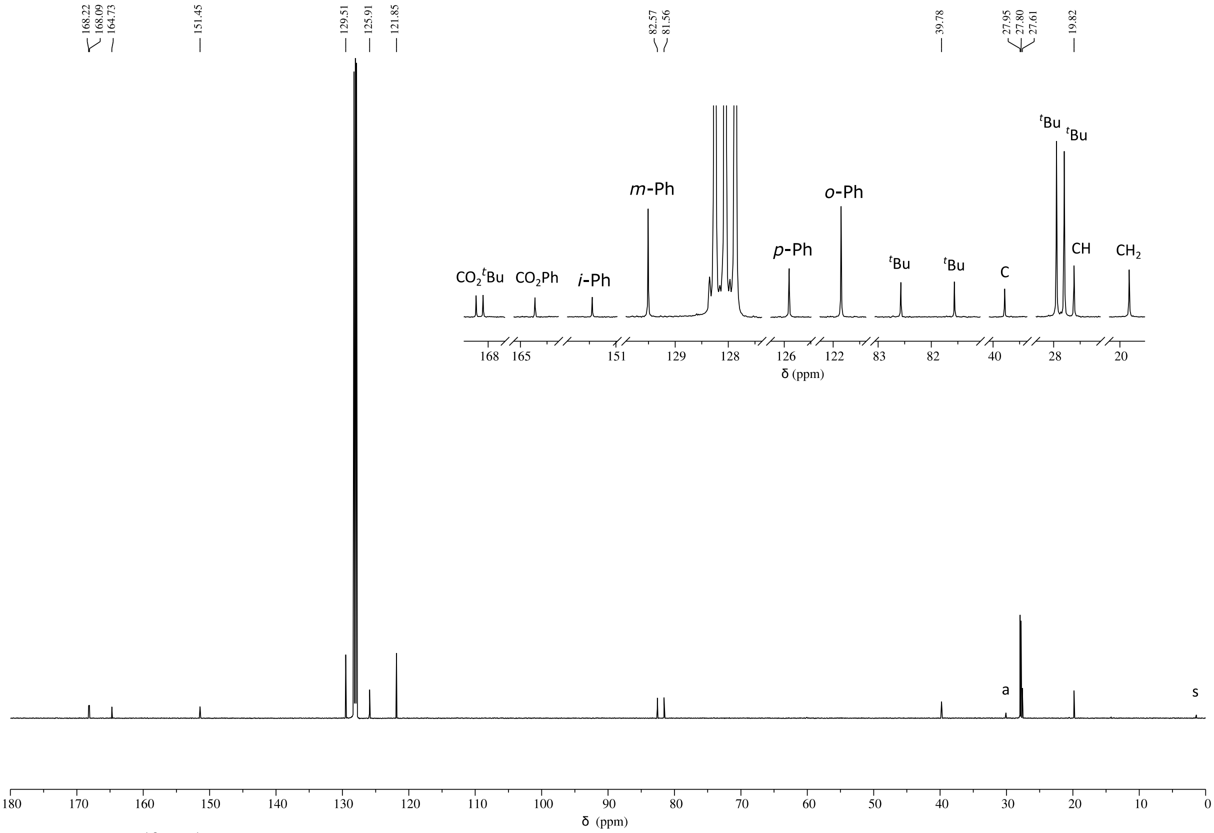

Figure S13.^{13}C{^{1}H} NMR (125 MHz, 298 K, C_6D_6) spectrum of compound (±)-**6ca**. [a: acetone; s: silica grease]

Synthesis of compound (±)-6da:

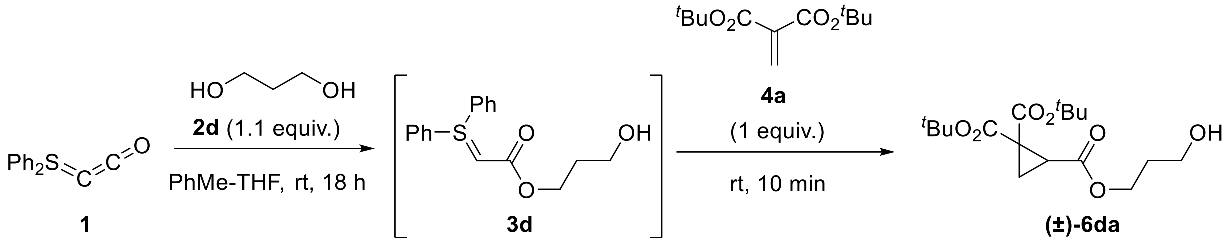

Reagent **1** (67.89 mg, 0.3 mmol, 1 equiv.) was dissolved in toluene-THF (2:1, 3 ml) and diol **2d** (25.11 mg, 0.33 mmol, 1.1 equiv.) was added and stirred for 18 h at room temperature. Then, compound **4a** (68.49 mg, 0.3 mmol, 1 equiv.) was added and reacted for 10 min at room temperature. The crude product was purified by silica gel column chromatography (ethyl acetate in pentane: 0% to 10% to 15% to 20% to 30% to 50%) to obtain compound (±)-**6da** (65.5 mg, 0.19 mmol, 63%) as a light-yellow oil.

Characterisation data of compound (±)-**6da**:

^{1}H NMR (500 MHz, C_6D_6, 298 K): δ = 4.20 (m, 1H, O*CH₂*) 4.02 (m, 1H, O*CH₂*) 3.38 (m, 2H, *CH₂*OH) 2.66 (dd, $^3J_{HH}$ = 6.7, $^3J_{HH}$ = 8.6 Hz, 1H, CH-CO_2R) 1.98 (dd, $^2J_{HH}$ = 4.4, $^3J_{HH}$ = 6.7 Hz, 1H, CH_2) 1.54 (m, 2H, OCH_2*CH₂*) 1.47 (s, 9H, *ᵗ*Bu) 1.40 (dd, $^2J_{HH}$ = 4.4, $^3J_{HH}$ = 8.5 Hz, 1H, CH_2) 1.26 (s, 9H, *ᵗ*Bu).
^{13}C{^{1}H} NMR (125 MHz, C_6D_6, 298 K): δ = 170.1 ($CO_2{}^t$Bu) 168.3 (*C*$O_2(CH_2)_3$OH) 165.0 ($CO_2{}^t$Bu) 82.4 (^{t}Bu) 81.4 (^{t}Bu) 62.3 (O*CH₂*) 58.7 (*CH₂*OH) 39.3 (C) 32.0 (OCH_2*CH₂*) 28.0 (^{t}Bu) 27.8 (^{t}Bu) 27.6 (*CH*-CO_2R) 19.6 (CH_2).
IR (ATR) ṽ [cm^{-1}] 3546, 2978, 1721, 1478, 1458, 1395, 1369, 1334, 1285, 1257, 1229, 1157, 1127, 1054, 994, 966, 916, 838, 757, 739, 523, 467, 412.
HR-MS-ESI (+) calc. $C_{17}H_{28}O_7Na^+$ [M+Na]$^+$ 367.1727; found 367.1728.

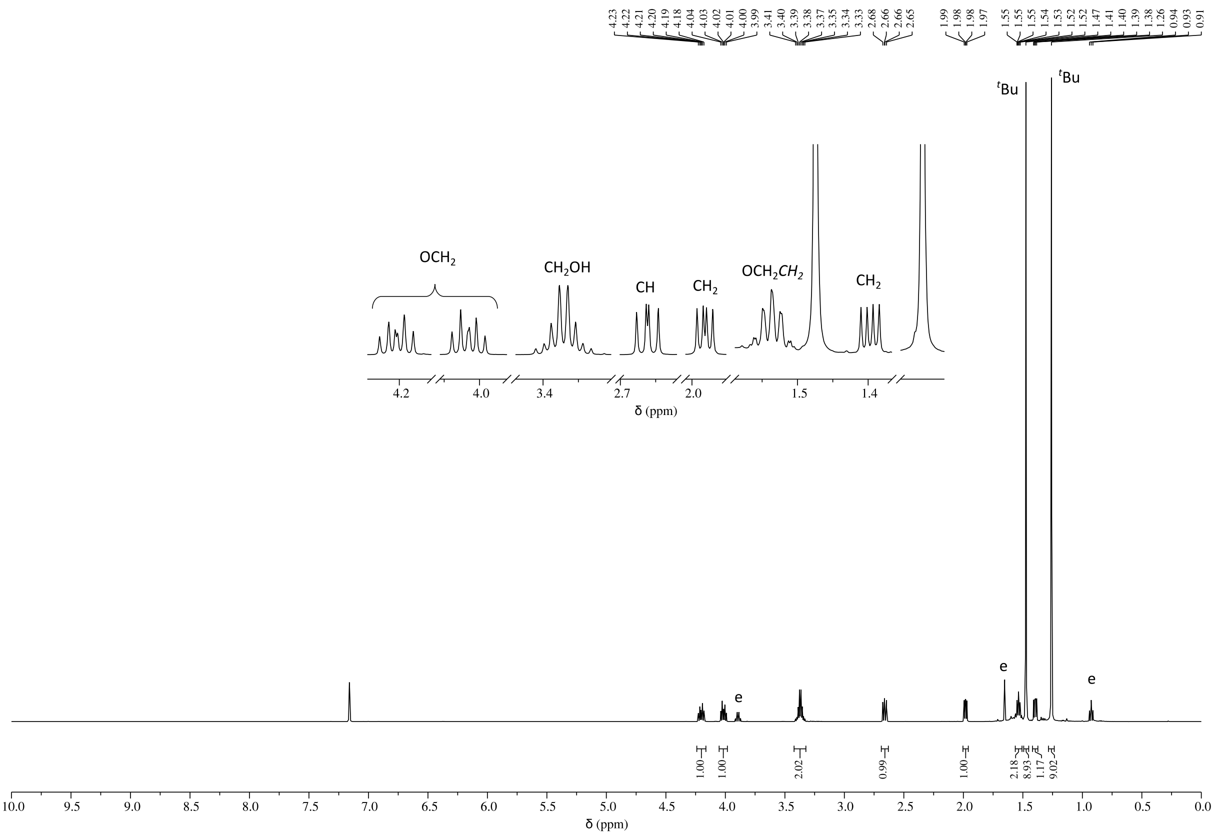

Figure S14. ^{1}H NMR (500 MHz, 298 K, C_6D_6) spectrum of compound (±)-**6da**. [e: ethyl acetate]

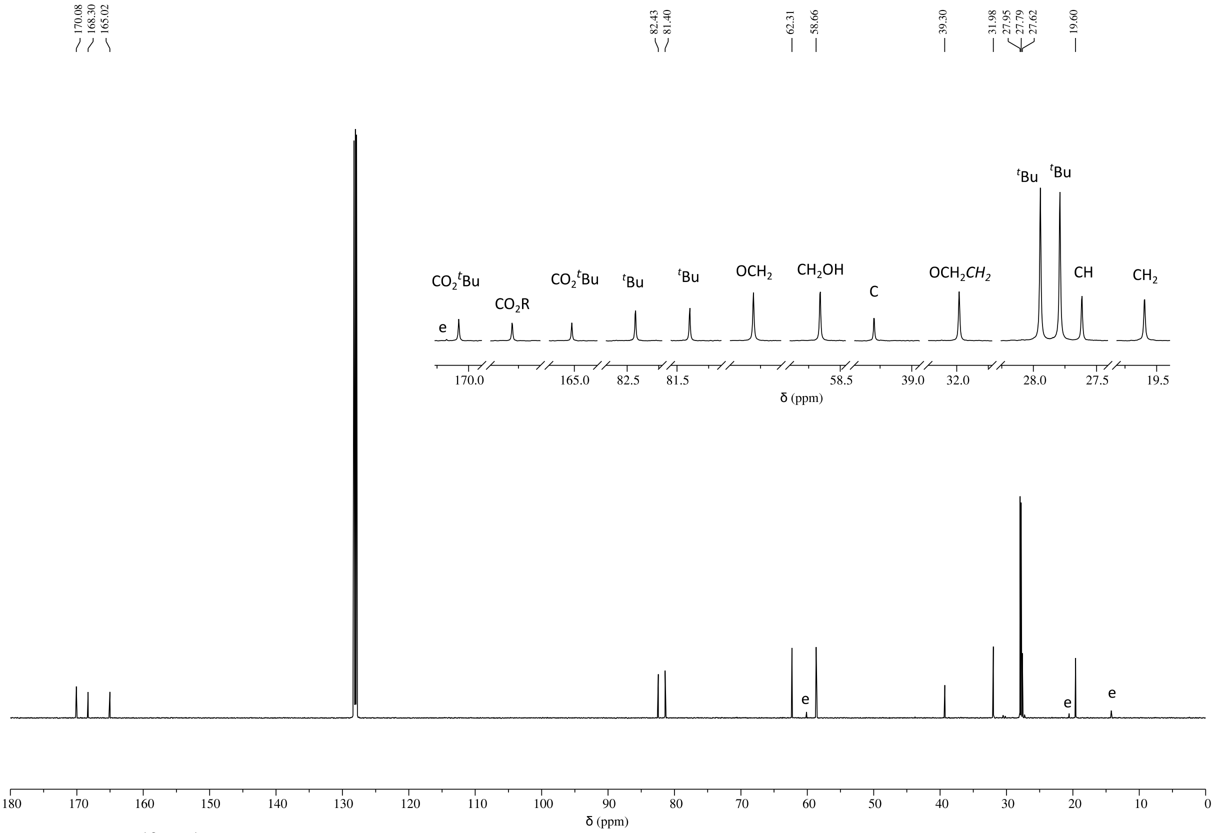

Figure S15.$^{13}C\{^1H\}$ NMR (125 MHz, 298 K, C_6D_6) spectrum of compound (±)-**6da**. [e: ethyl acetate]

Synthesis of compound (±)-6ea

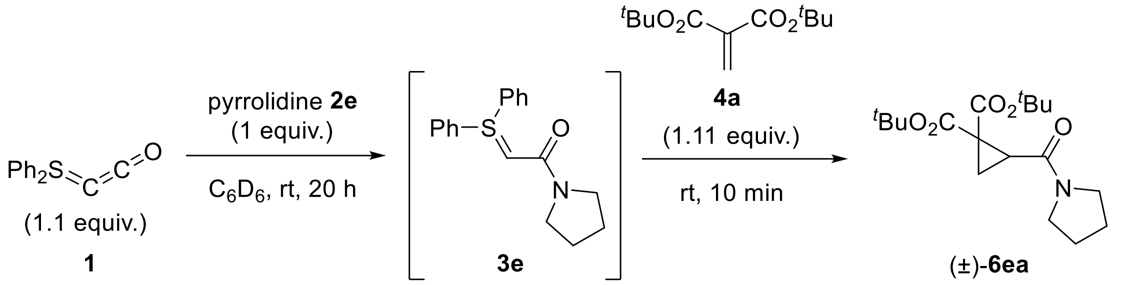

Reagent **1** (23.59 mg, 104.24 µmol, 1.1 equiv.) was dissolved in C_6D_6 (0.5 ml) and compound **2e** (6.74 mg, 0.86 g/ml, 7.84 µl, 94.77 µmol, 1.0 equiv.) was added and reacted for 20 h at room temperature. Then, compound **4a** (23.8 mg, 104.24 µmol, 1.1 equiv.) was added and the reaction was reacted further 10 min at room temperature. The formed product was purified by silica gel column chromatography (ethyl acetate in cyclohexane: 0% to 10 % to 20%) to obtain compound (±)-**6ea** (23.4 mg, 68.94 µmol, 73%) as a colorless solid.

Characterisation data of compound (±)-**6ea**:

m.p.: 83 °C.
^{1}H NMR (500 MHz, 298 K, C_6D_6): δ = 3.37 (m, 1H, N-CH_2) 3.28 (m, 1H, N-CH_2) 3.17 (m, 1H, N-CH_2) 2.85 (m, 1H, N-CH_2) 2.55 (dd, $^3J_{HH}$ = 6.8 Hz, $^3J_{HH}$ = 8.5 Hz, 1H, CH) 2.41 (dd, $^2J_{HH}$ = 3.9 Hz, $^3J_{HH}$ = 6.8 Hz, 1H, CH_2) 1.50 (s, 9H, ^{t}Bu) 1.44 (dd, $^2J_{HH}$ = 3.9 Hz, $^3J_{HH}$ = 8.4 Hz, 1H, CH_2) 1.36 (s, 9H, ^{t}Bu) 1.21 (m, 4H, CH_2CH_2).
^{13}C{^{1}H} NMR (125 MHz, 298 K, C_6D_6): δ = 169.5 (C(O)NR_2) 165.7 ($CO_2{}^t$Bu) 165.4 ($CO_2{}^t$Bu) 81.8 (^{t}Bu) 80.6 (^{t}Bu) 46.2 (N-CH_2) 46.1 (N-CH_2) 38.5 (C) 28.5 (*CH*) 28.1 (^{t}Bu) 27.9 (^{t}Bu) 26.0 (CH_2CH_2) 24.4 (CH_2CH_2) 18.8 (CH_2).
IR (ATR) ṽ [cm^{-1}] 2977, 2936, 2878, 1738, 1713, 1650, 1454, 1417, 1390, 1363, 1352, 1331, 1283, 1256, 1229, 1214, 1161, 1128, 1069, 1047, 1003, 846, 835, 810, 778, 760, 746, 738, 535, 519, 468, 414.
HR-MS-ESI (+) calc. $C_{18}H_{29}NO_5Na^+$ $[M+Na]^+$ 362.1938; found 362.1935.

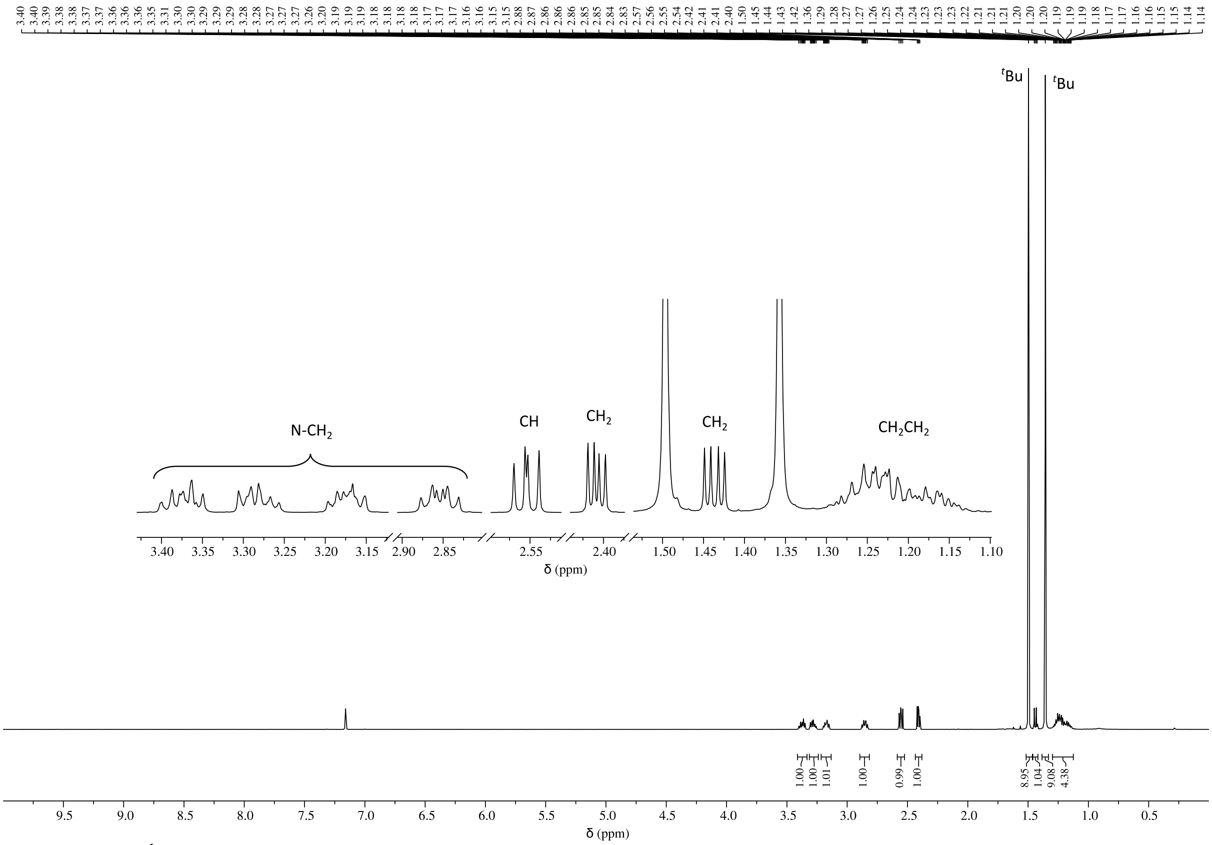

Figure S16. ^{1}H NMR (500 MHz, 298 K, C_6D_6) spectrum of compound (±)-**6ea**.

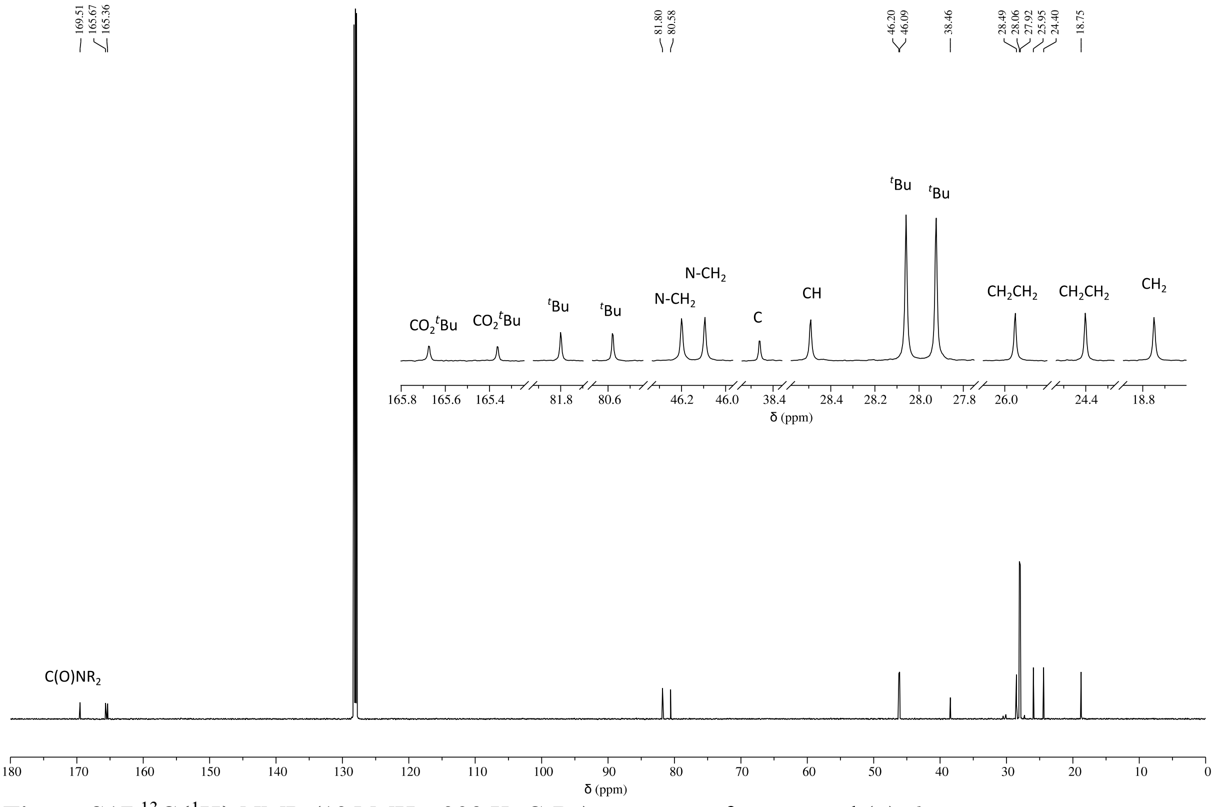

Figure S17.^{13}C{^{1}H} NMR (125 MHz, 298 K, C_6D_6) spectrum of compound (±)-**6ea**.

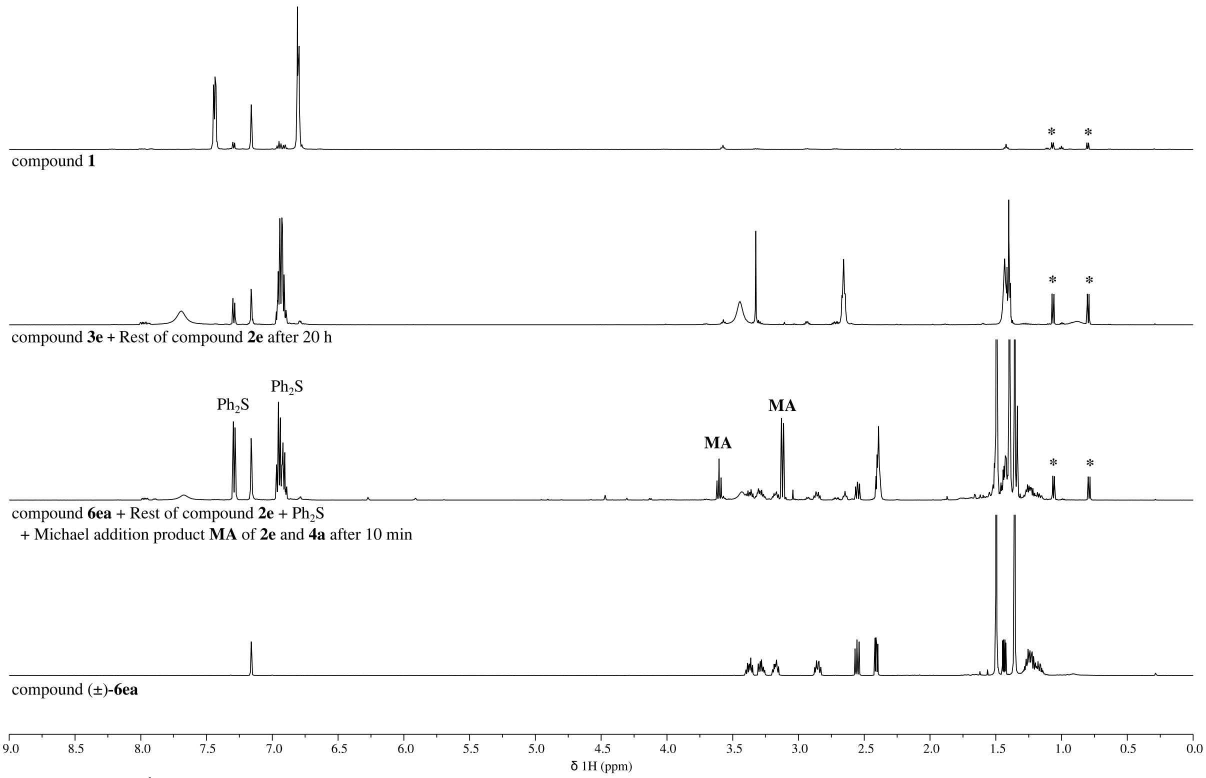

Figure S18. ^{1}H NMR spectra (500 MHz, 298 K, C_6D_6) of the in-situ reaction for the formation of compound (±)-**6ea**. ^{1}H-NMR spectra (from top to bottom) of compound **1**; 20 h after addition of compound **2e** to form compound **3e**; 10 min after addition of compound **4a** to form product (±)-**6ea**; compound (±)-**6ea** after purification. Due to the reaction of compound **2e** with compound **4a**, 0.9 equiv. of compound **2e** were used in the main reaction, to ensure an access of compound **4a** and complete product formation.

Synthesis of compound (±)-6fa:

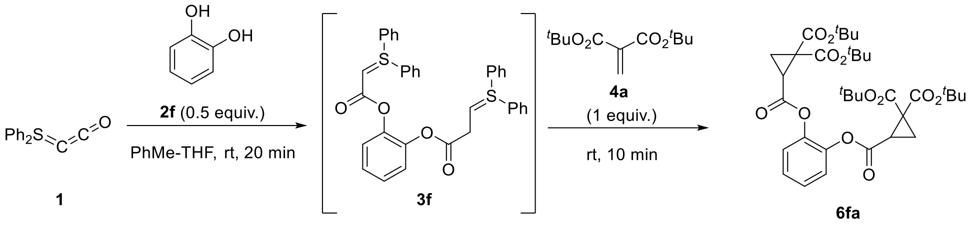

Reagent **1** (67.89 mg, 0.3 mmol, 1.0 equiv.) was dissolved in toluene-thf (2:1, 3 ml) and catechol **2f** (16.52 mg, 0.15 mmol, 0.5 equiv.) was added. After 20 min stirring at room temperature, compound **4a** (68.49 mg, 0.3 mmol, 1 equiv.) was added and reacted for further 10 min at room temperature. The formed product was purified by silica gel column chromatography (ethyl acetate in pentane: 0% to 2% to 5% to 8%) to obtain the mixture of compounds **6fa** (69.4 mg, 107.31 µmol, 72%) as a sticky colourless oil (*in-situ* diastereomeric ratio = 1.6:1)

Characterisation data of compounds **6fa**:

^{1}H NMR (600 MHz, C_6D_6, 298 K): δ = 7.24 (m, 1H+1H′, Ar) 7.16 (m, 1H+1H′, Ar) 6.76 (m, 2H+2H′, Ar) 2.99 (m, 2H+2H′, each *CH*CO_2Ar) 2.09 (m, 2H+2H′, CH_2) 1.54 (m, 2H+2H′, CH_2) 1.45 (s, 9H, ^{t}Bu) 1.44 (s, 9H, ^{t}Bu′) 1.30 (s, 9H, ^{t}Bu) 1.28 (s, 9H, ^{t}Bu′).
^{13}C{^{1}H} NMR (150 MHz, C_6D_6, 298 K): δ = 168.0 ($CO_2{}^t$Bu) 167.9 ($CO_2{}^t$Bu′) 167.8 ($CO_2{}^t$Bu) 167.6 ($CO_2{}^t$Bu′) 164.62 (CO_2Ar′) 164.60 (CO_2Ar) 142.9 (Ar-C) 142.8 (Ar-C′) 126.7 (Ar) 126.5 (Ar′) 123.8 (Ar) 123.7 (Ar′) 82.54 (^{t}Bu) 82.53 (^{t}Bu′) 81.7 (^{t}Bu) 81.6 (^{t}Bu′) 39.87 (C) 39.85 (C′) 27.9 (^{t}Bu+ ^{t}Bu′) 27.8 (^{t}Bu+ ^{t}Bu′) 27.3 (*CH*CO_2Ar′) 27.2 (*CH*CO_2Ar) 20.21 (CH_2) 20.16 (CH_2′).
IR (ATR) ṽ [cm^{-1}] 2918, 2849, 1769, 1723, 1493, 1459, 1390, 1369, 1334, 1286, 1243, 1221, 1166, 1122, 1101, 1036, 992, 838, 810, 769, 739, 466.
HR-MS-ESI (+) calc. $C_{34}H_{46}O_{12}Na^+$ $[M+Na]^+$ 669.2881; found 669.2884.

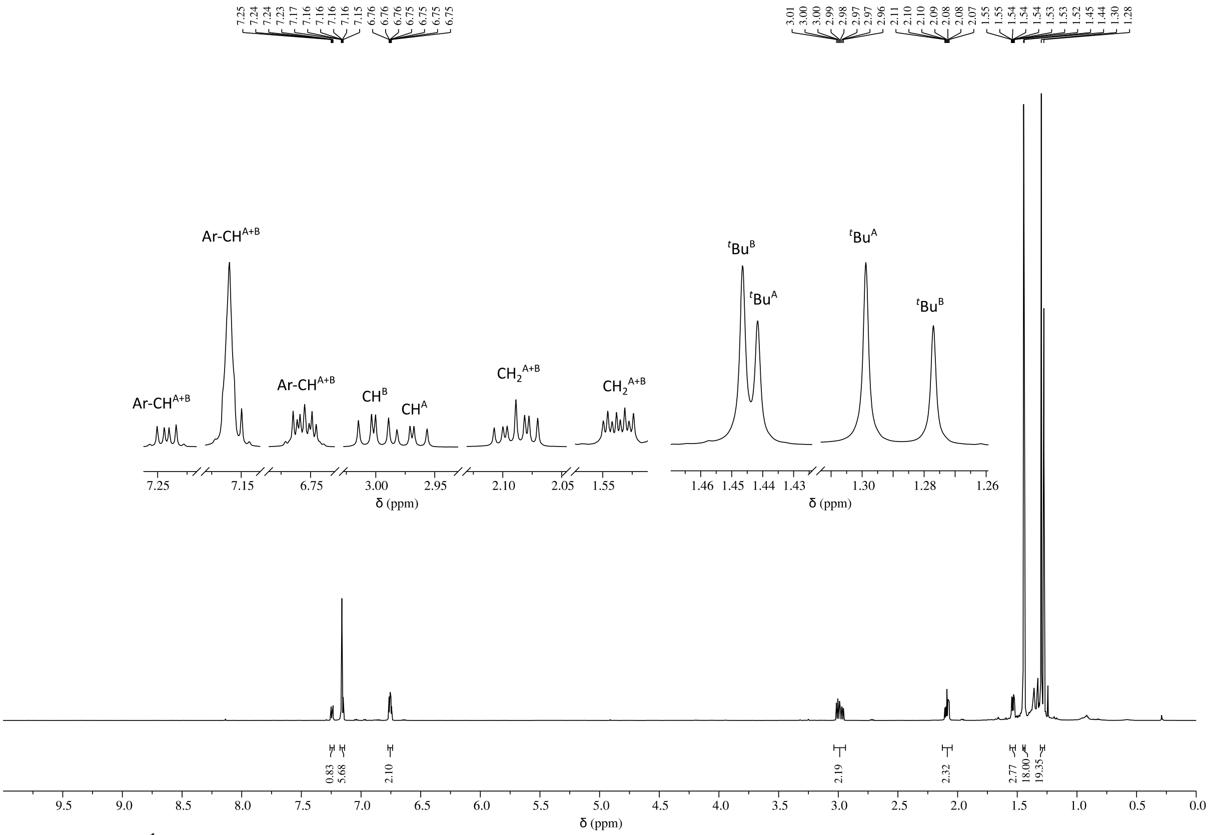

Figure S19. ^{1}H NMR (600 MHz, 298 K, C_6D_6) spectrum of major and minor diastereomers of compounds **6fa**.

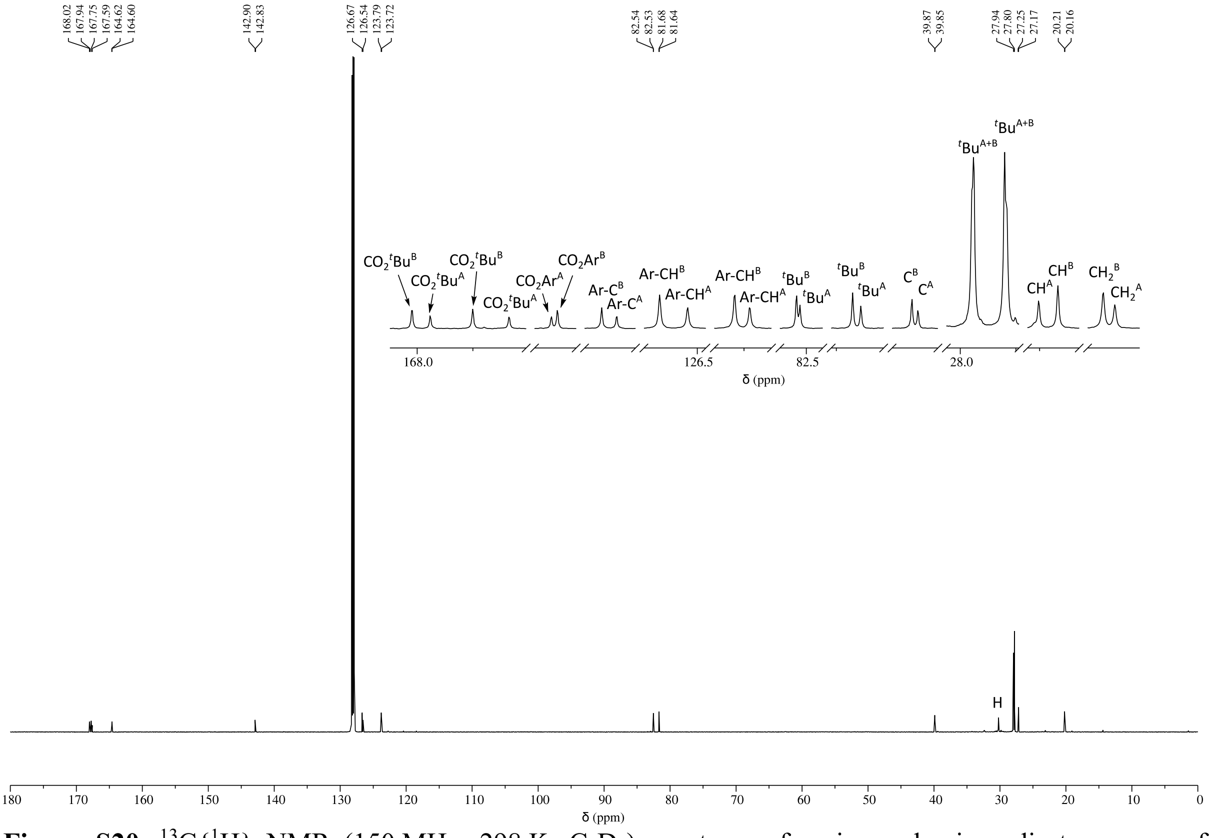

Figure S20. $^{13}C\{^{1}H\}$ NMR (150 MHz, 298 K, C_6D_6) spectrum of major and minor diastereomers of compounds **6fa**. [H: H-grease]

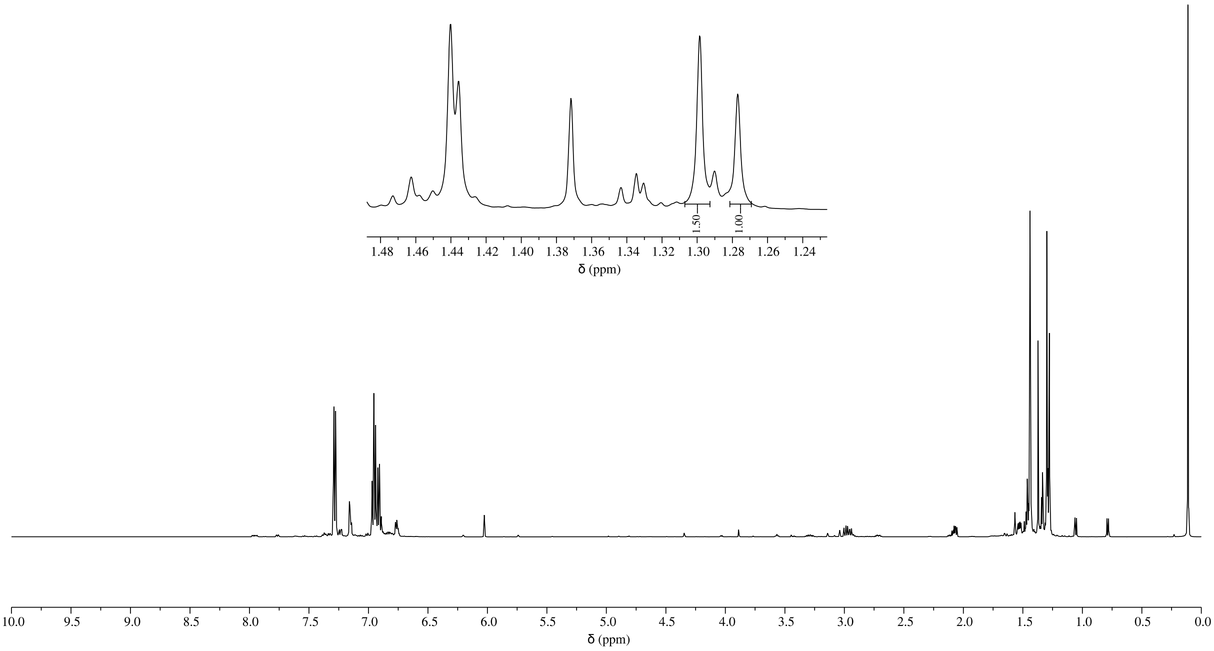

Figure S21. ^{1}H NMR (600 MHz, 298 K, C_6D_6) spectrum of the in-situ reaction to synthesis compounds **6fa**.

Synthesis of compound (±)-6ga:

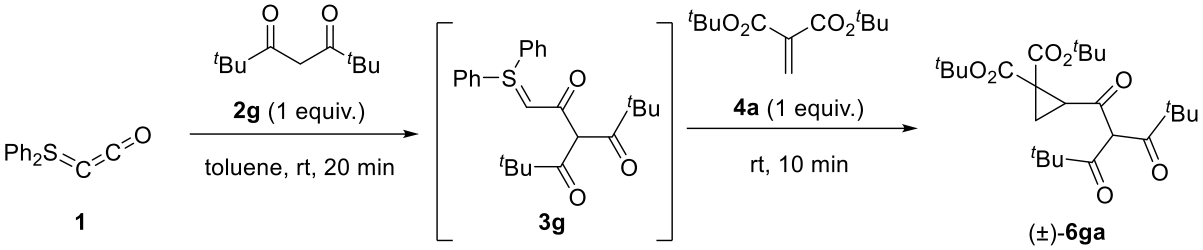

Reagent **1** (67.89 mg, 0.3 mmol, 1 equiv.) was dissolved in toluene (3 ml) and diketone **2g** (55.28 mg, 0.3 mmol, 1 equiv.) was added. After 20 min stirring at room temperature, compound **4a** (68.49 mg, 0.3 mmol, 1 equiv.) was added and reacted for further 10 min at room temperature. The formed product was purified by silica gel column chromatography (ethyl acetate in pentane: 0% to 2% to 4% to 6% to 8%) to obtain the product (±)-**6ga** (103.4 mg, 0.228 mmol, 76%) as a colourless oil.

Characterization data of compound (±)-**6ga**:

^{1}H NMR (500 MHz, $CDCl_3$, 298 K): δ = 6.21 (s, 1H, CH(C(O)R)$_3$) 2.73 (dd, $^3J_{HH}$ = 6.6, $^3J_{HH}$ = 8.9 Hz, 1H, CH^{cyclopropane}) 1.92 (dd, $^2J_{HH}$ = 4.5, $^3J_{HH}$ = 6.6 Hz, 1H, CH_2) 1.66 (dd, $^2J_{HH}$ = 4.5, $^3J_{HH}$ = 8.9 Hz, 1H, CH_2) 1.47 (s, 9H, $CO_2{}^t$Bu) 1.42 (s, 9H, $CO_2{}^t$Bu) 1.12 (m, br, 18H, ^{t}Bu).
^{13}C{^{1}H} NMR (125 MHz, $CDCl_3$, 298 K): δ = 202.3 (br, *C*(O)^{t}Bu) 168.1 (CH*C*(O)CH) 167.7 (br, *C*(O)^{t}Bu) 166.8 (*C*O$_2{}^t$Bu) 165.0 (*C*O$_2{}^t$Bu) 105.6 (*CH*(C(O)R)$_3$) 82.9 (CO$_2{}^t$Bu) 81.7 (CO$_2{}^t$Bu) 44.1 (br, ^{t}Bu) 39.2 (C) 37.6 (br, ^{t}Bu) 28.1 (CO$_2{}^t$Bu) 27.9 (CO$_2{}^t$Bu) 27.6 (br, ^{t}Bu) 27.3 (CH^{cyclopropane}) 26.5 (br, ^{t}Bu) 20.7 (CH_2).
IR (ATR) $\tilde{\nu}$ [cm^{-1}] 2978, 1771, 1727, 1692, 1618, 1480, 1394, 1369, 1336, 1287, 1259, 1221, 1173, 1130, 1079, 984, 867, 841, 739, 466.
HR-MS-ESI (+) calc. $C_{23}H_{18}O_4Na^+$ [M+Na]$^+$ 475.2666; found 475.2663.

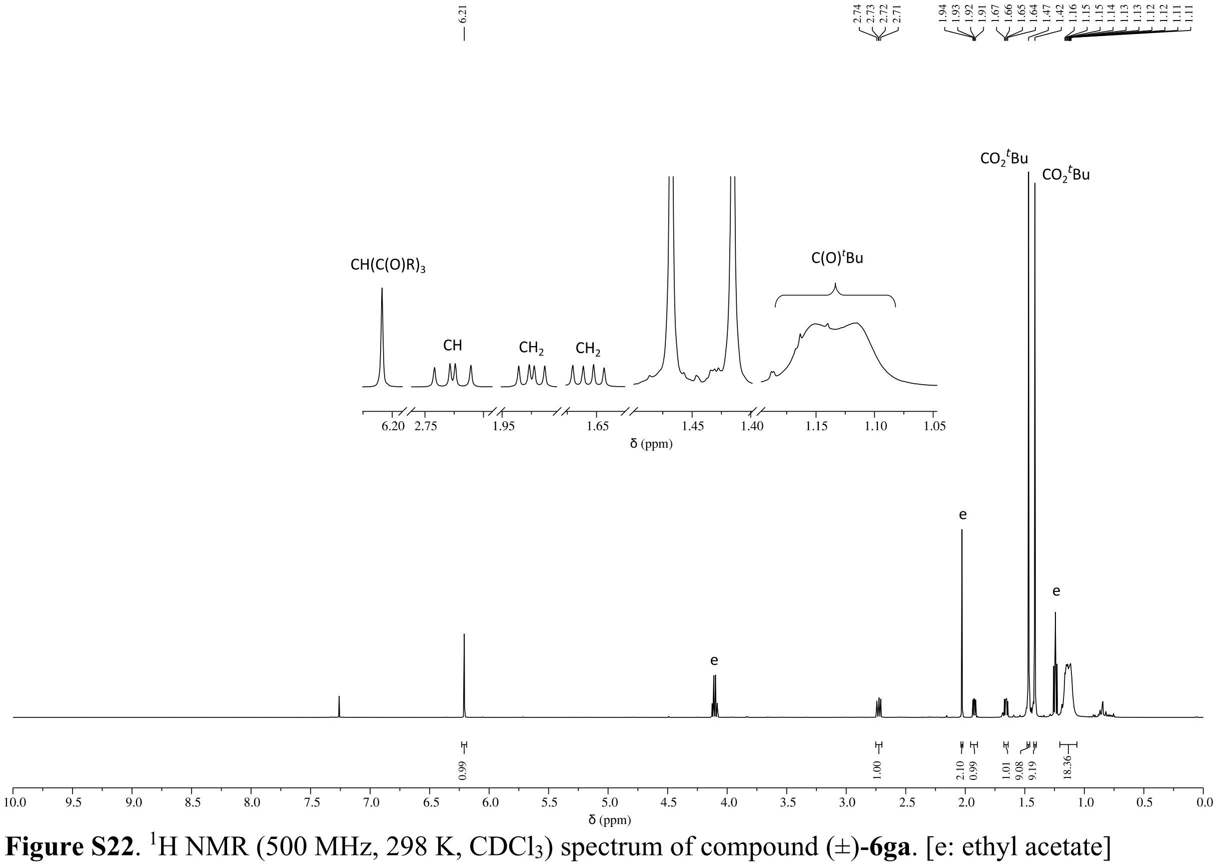

Figure S22. ^{1}H NMR (500 MHz, 298 K, $CDCl_3$) spectrum of compound (±)-**6ga**. [e: ethyl acetate]

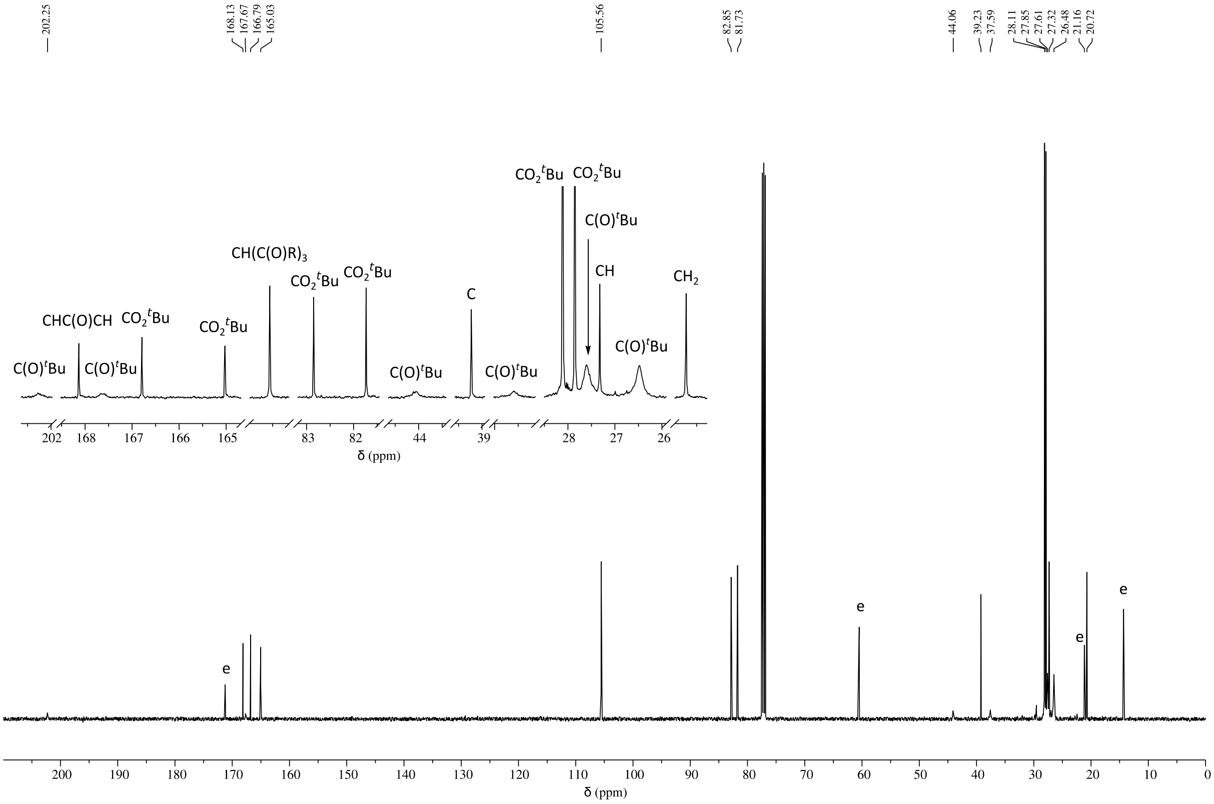

Figure S23.^{13}C{^{1}H} NMR (125 MHz, 298 K, $CDCl_3$) spectrum of compound (±)-**6ga**. [e: ethyl acetate]

Synthesis of compound (±)-6ab:

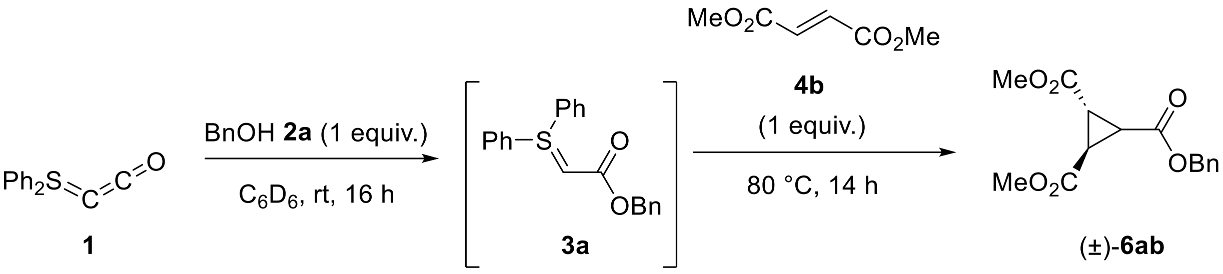

Reagent **1** (21.43 mg, 95.7 µmol, 1 equiv.) was dissolved in C_6D_6 (0.5 ml) and compound **2a** (10.24 mg, 95.7 µmol, 1 equiv.) was added and stirred for 16 h at room temperature. Then, dimethyl fumarate **4b** (13.65 mg, 95.7 µmol, 1 equiv.) was added and the reaction mixture was heated up to 80 °C for 14 h. The product was purified by silica gel column chromatography (ethyl acetate in cyclohexane: 0% to 5% to 10% to 15%) to obtain the desired product (±)-**6ab** (19.4 mg, 66.37 µmol, 70%) as a light-yellow oil.

Characterization data of compound (±)-**6ab**:

^{1}H NMR (500 MHz, C_6D_6, 298 K): δ = 7.12 (m, 2H, Ph-CH) 7.05 (m, 3H, Ph-CH) 4.94 (m, 2H, CH_2) 3.21 (s, 3H, CO_2Me) 3.15 (s, 3H, CO_2Me) 3.01 (t, $^3J_{HH}$ = 5.6 Hz, 1H, $CHCO_2Bn$) 2.47 (m, 2H, $CHCO_2Me$).
^{13}C{^{1}H} NMR (125 MHz, C_6D_6, 298 K): δ = 170.1 (CO$_2$Me) 167.6 (CO$_2$Me) 167.3 (CO$_2$Bn) 136.0 (Ph-C) 128.8 (Ph-CH) 128.7 (Ph-CH) 128.4 (Ph-CH) 67.3 (OCH_2Ph) 51.93 (CO_2Me) 51.86 (CO_2Me) 28.7 (CHCO$_2$Me) 28.6 (CHCO$_2$Me) 25.8 (CHCO$_2$Bn).
IR (ATR) ṽ [cm^{-1}] 2955, 1725, 1455, 1437, 1382, 1354, 1308, 1277, 1193, 1158, 1056, 1025, 926, 899, 827, 803, 750, 698, 673, 600.
HR-MS-ESI (+) calc. $C_{15}H_{16}O_6Na^+$ $[M+Na]^+$ 315.0839; found 315.0837.

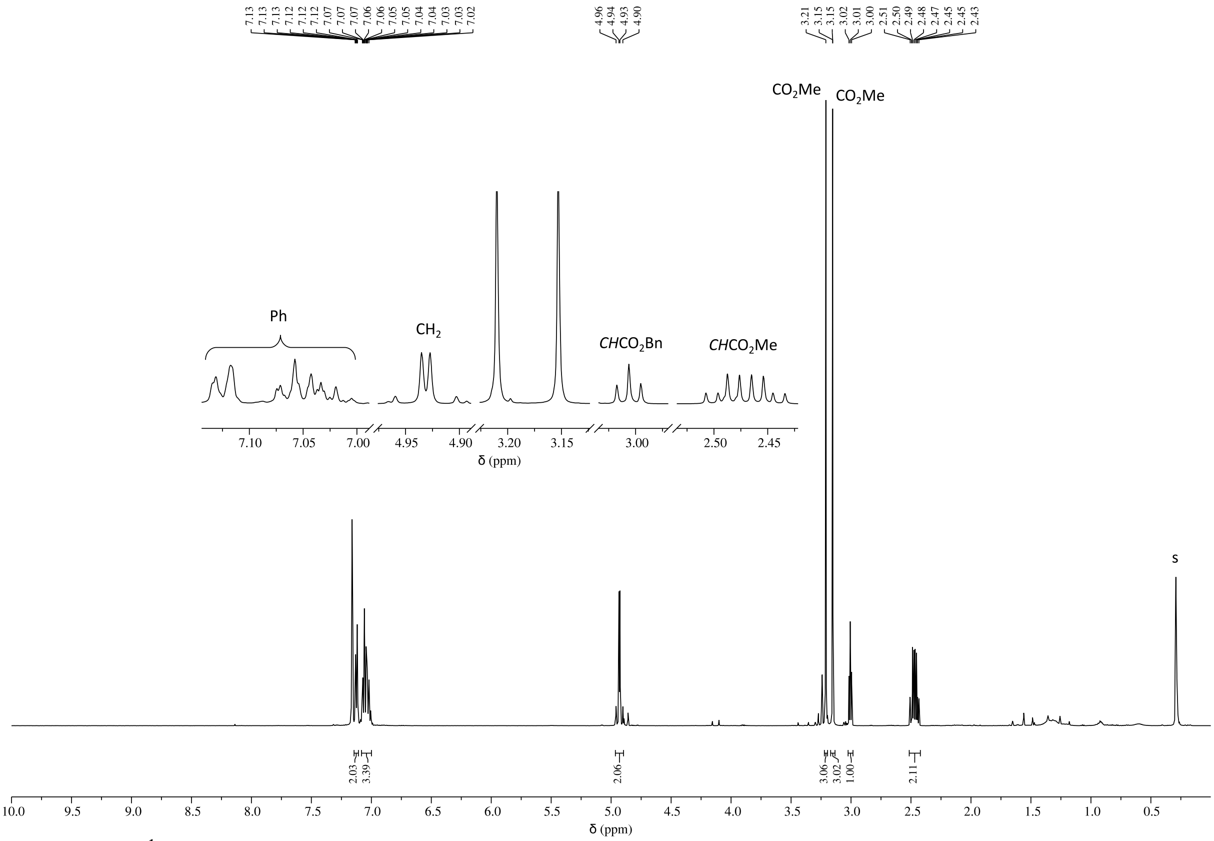

Figure S24. ^{1}H NMR (500 MHz, 298 K, C_6D_6) spectrum of compound (±)-**6ab**. [s: silicon grease]

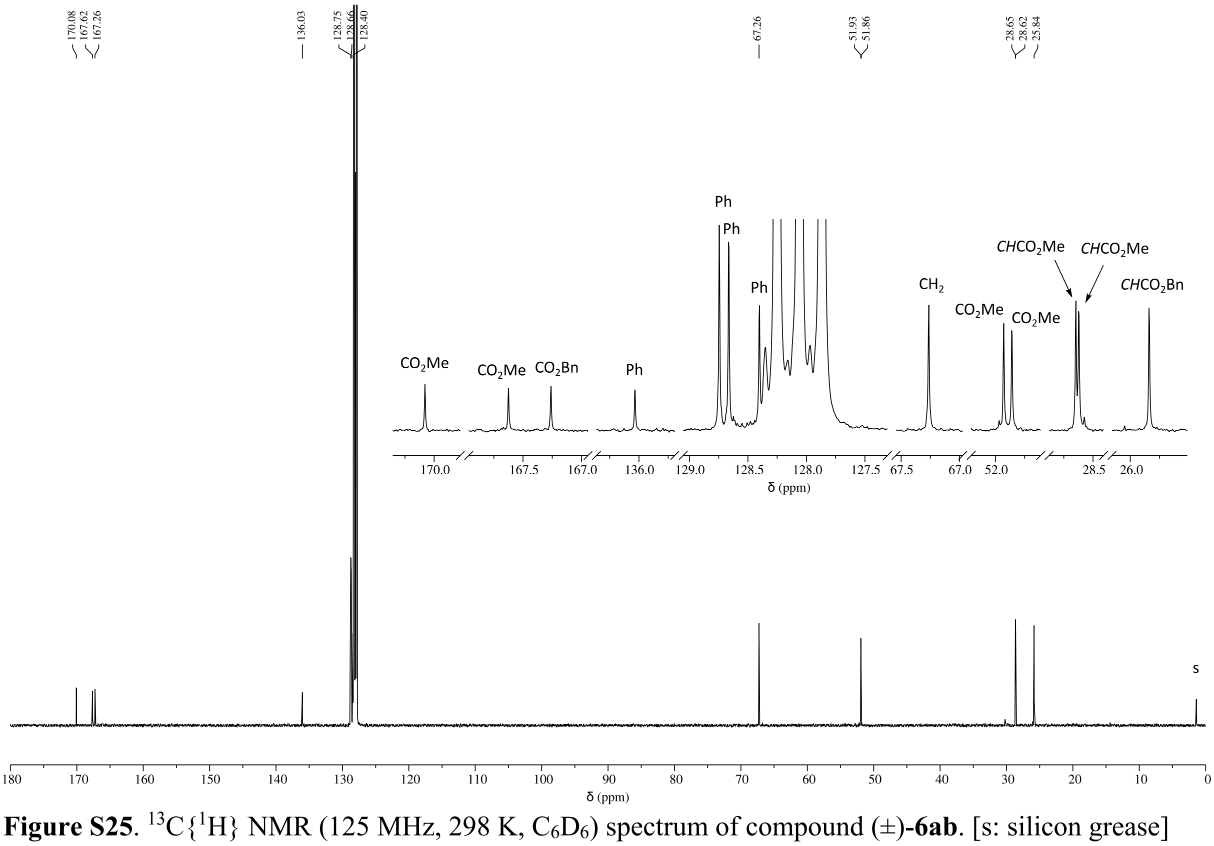

Figure S25. $^{13}C\{^1H\}$ NMR (125 MHz, 298 K, C_6D_6) spectrum of compound (±)-**6ab**. [s: silicon grease]

Synthesis of compound (±)-6ac:

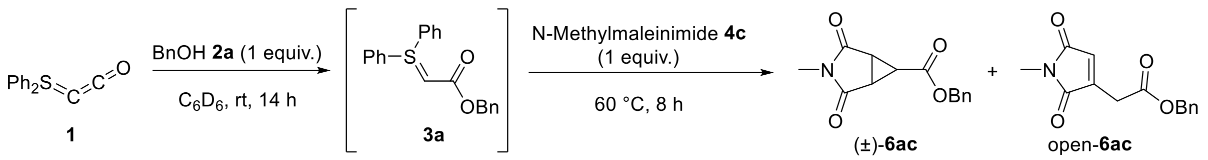

Reagent **1** (21.43 mg, 94.7 µmol, 1 equiv.) was dissolved in C_6D_6 (0.5 ml) and benzyl alcohol **2a** (10.24 mg, 94.7 µmol, 1 equiv.) was added and stirred at room temperature. After 14 hours *N*-methylmaleinimid **4c** (10.52 mg, 94.7 µmol, 1 equiv.) was added. The reaction was stirred for 8 h at 60 °C and then purified by silica gel column chromatography (ethyl acetate in cyclohexane: 0% to 10% to 15% to 20% to 25%). The main product *cis*-(±)-**6ac** (10.6 mg, 40.89 µmol, 43%) could be obtained as a colorless solid. The mixture of *trans*-(±)-**6ac** and open-**6ac** (9.7 mg, 37.41 µmol, 40%, ratio = 1.3:1) could be obtained as colourless oil. The *in situ* ^{1}H-NMR spectrum shows a ratio of 1.53:1:0.1.

Characterization data of compound *cis*-(±)-**6ac**:
^{1}H NMR (500 MHz, C_6D_6, 298 K): δ = 7.02 (m, 5H, 5H, Ph) 4.71 (s, 2H, CH_2) 2.64 (s, 3H, CH_3) 1.77 (d, $^3J_{HH}$ = 8.3 Hz, 2H, CH) 1.53 (t, $^3J_{HH}$ = 8.3 Hz, 1H, $CHCO_2Bn$).
$^{13}C\{^1H\}$ NMR (125 MHz, C_6D_6, 298 K): δ = 170.8 (*C*ONR_2) 167.5 (*C*O_2Bn) 135.4 (Ph) 128.9 (Ph) 128.8 (Ph) 128.7 (Ph) 67.6 (CH_2) 31.0 (*CH*CO_2Bn) 26.7 (CH) 24.5 (CH_3).
IR (ATR) ṽ [cm^{-1}] 3029, 2958, 2924, 2854, 1777, 1721, 1698, 1498, 1439, 1387, 1371, 1302, 1273, 1261, 1213, 1132, 1081, 1028, 995, 966, 941, 889, 859, 837, 803, 770, 752, 701, 682, 623, 602, 562, 540, 498.
HR-MS-ESI (+) calc. $C_{14}H_{13}NO_4Na^+$ $[M+Na]^+$ 282.0737; found 282.0732.

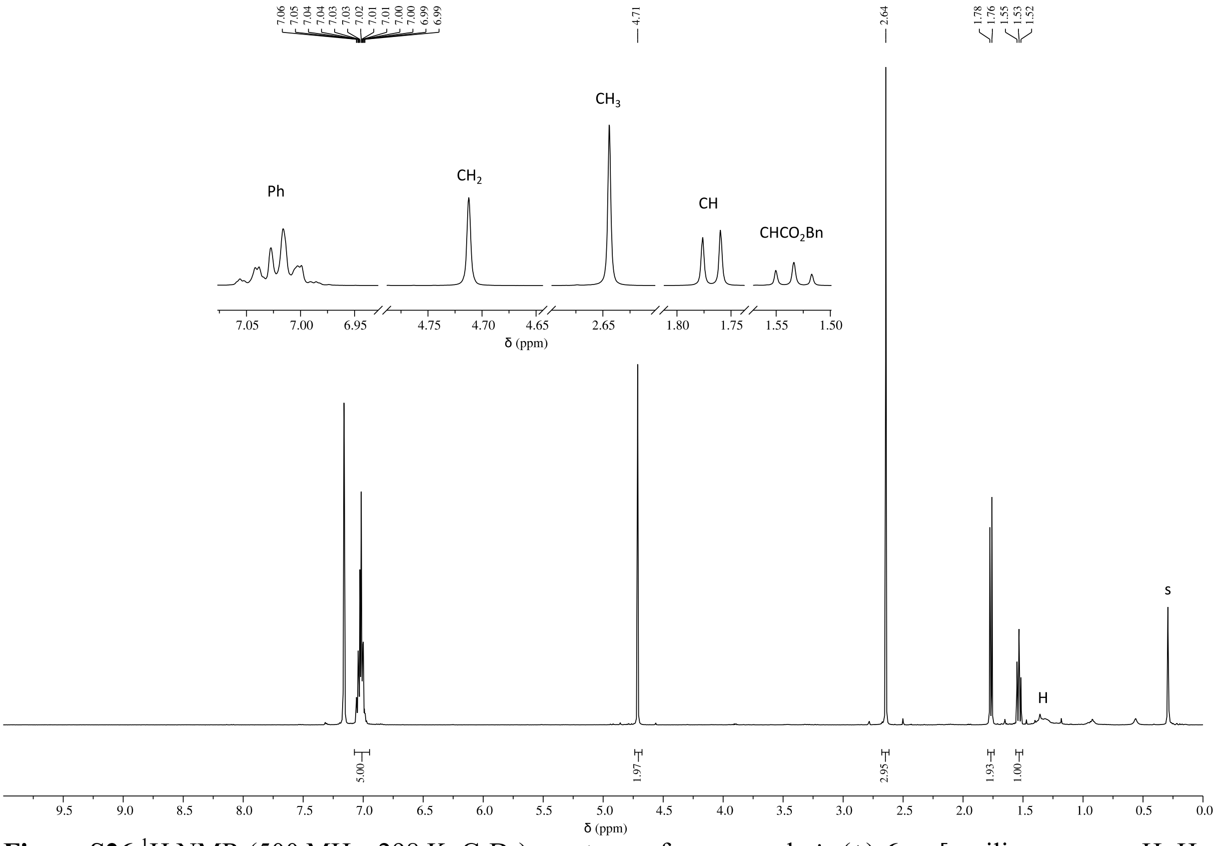

Figure S26. ^{1}H NMR (500 MHz, 298 K, C_6D_6) spectrum of compound *cis*-(±)-**6ac**. [s: silicon grease, H: H-grease]

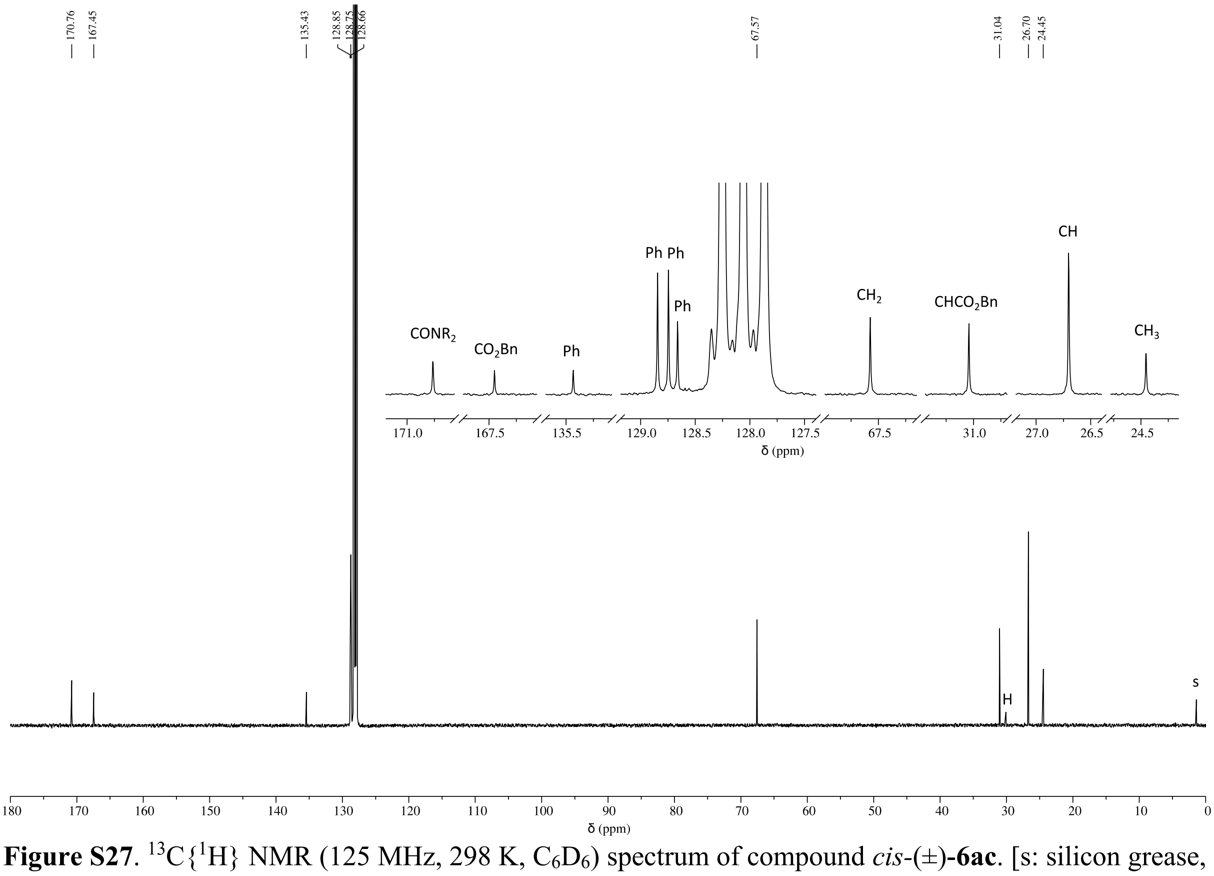

Figure S27. $^{13}C\{^1H\}$ NMR (125 MHz, 298 K, C_6D_6) spectrum of compound *cis*-(±)-**6ac**. [s: silicon grease, H: H-grease]

Characterization data of compound mixture *trans*-(±)-**6ac** and open-**6ac**:

^{1}H NMR (501 MHz, $CDCl_3$, 298 K): δ = 7.40–7.32 (m, 10H, 5H^{trans}, 5H^{open}, Ph) 6.86 (t, $^3J_{HH}$ = 2.6 Hz, *CH*$CONR_2^{open}$) 5.24 (s, 2H, *CH_2*Ph^{open}) 5.16 (s, 2H, *CH_2*Ph^{trans}) 3.68 (d, $^3J_{HH}$ = 2.6 Hz, 2H, CH_2^{open}) 3.11 (s, 3H, Me^{open}) 2.90 (d, $^3J_{HH}$ = 2.8 Hz, 2H, CH^{trans}) 2.86 (s, 3H, Me^{trans}) 2.44 (t, $^3J_{HH}$ = 2.8 Hz, *CH*CO_2Bn^{trans}) **$^{13}C\{^1H\}$ NMR** (126 MHz, C_6D_6, 298 K): δ = 173.6 (C(O)NR_2^{open}) 172.5 (C(O)NR_2^{trans}) 169.1 (C(O)NR_2^{open}) 167.3 (CO_2Bn ^{trans}) 164.8 (CO_2Bn^{open}) 140.7 (C^{open}) 135.2 (Ph^{open}) 134.8 (Ph ^{trans}) [128.93, 128.89, 128.88, 128.8, 128.6, 128.5] (Ph ^{trans} +Ph^{open}) 122.1 (*CH*$CONR_2^{open}$) 68.0 (CH_2 ^{trans}) 67.3 (CH_2Ph^{open}) 34.5 (CH_2^{open}) 32.6 (*CH*CO_2Bn ^{trans}) 26.7 (CH ^{trans}) 25.2 (N-CH_3^{open}) 24.7 (N-CH_3 ^{trans}).

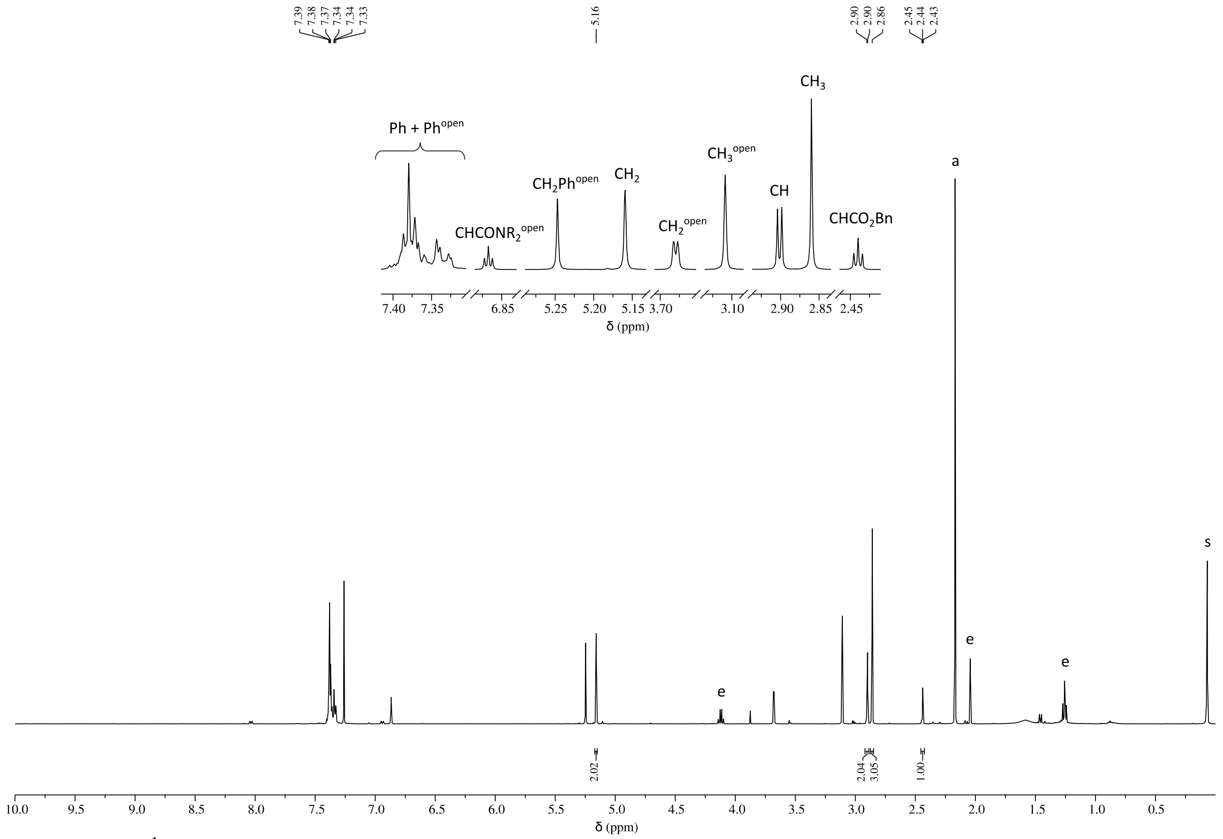

Figure S28. ^{1}H NMR (500 MHz, 298 K, $CDCl_3$) spectrum of compound *trans*-(±)-**6ac**, mixed with the ring opening product open-**6ac.** [s: silicon grease, a: acetone, e: ethylacetate]

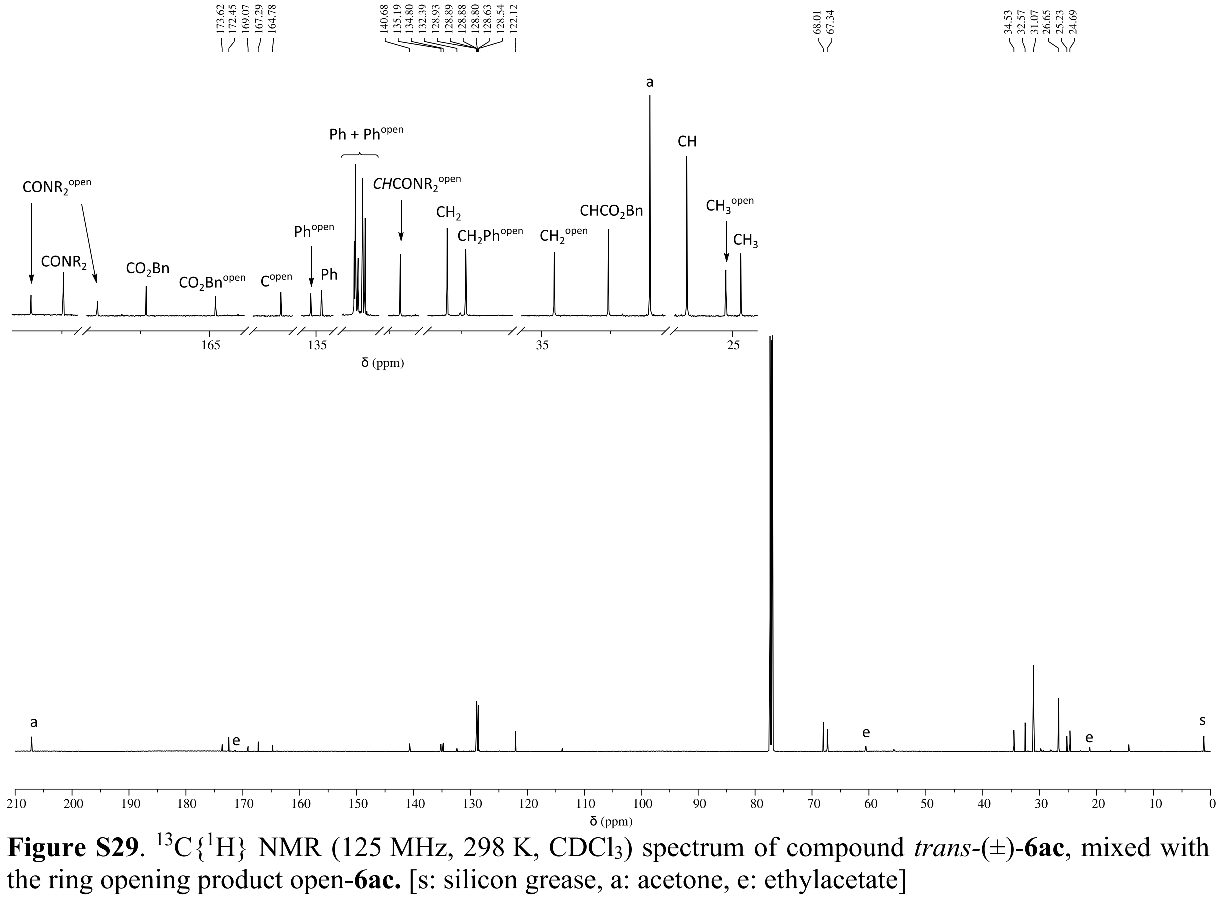

Figure S29. $^{13}C\{^{1}H\}$ NMR (125 MHz, 298 K, $CDCl_3$) spectrum of compound *trans*-(±)-**6ac**, mixed with the ring opening product open-**6ac.** [s: silicon grease, a: acetone, e: ethylacetate]

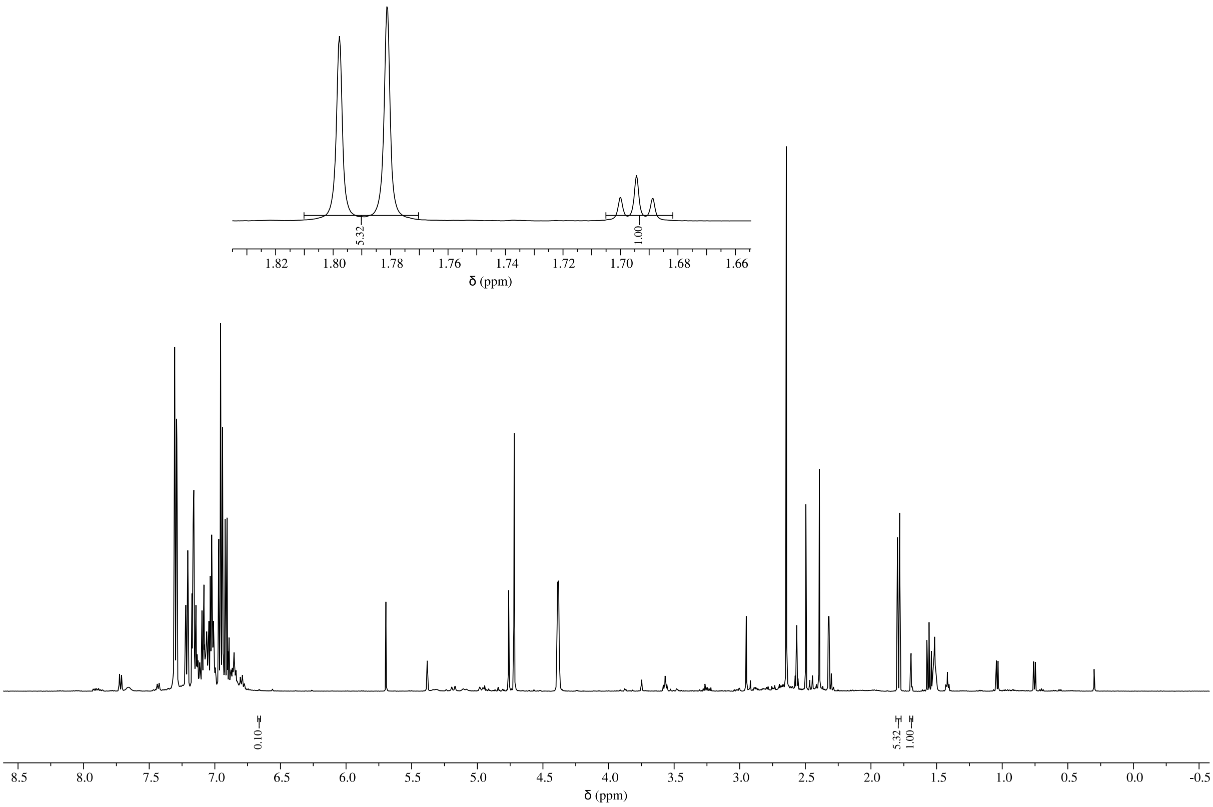

Figure S30.^{1}H NMR (500 MHz, 298 K, C_6D_6) spectrum of the in-situ reaction to synthesis compounds (±)-**6ae**. It shows a ratio of 1.53 : 1 : 0.1 between the two diastereomers and the ring opening product open-**6ac.**

Synthesis of compound (±)-6ad:

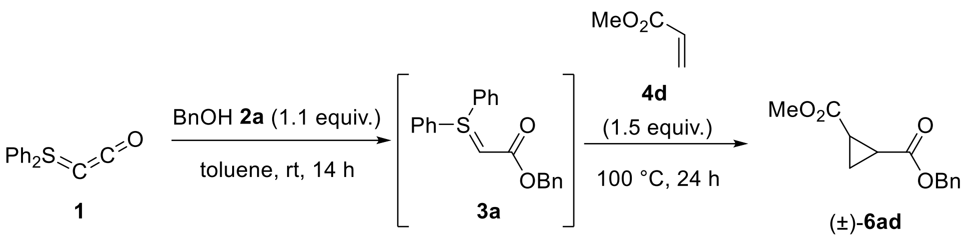

Reagent **1** (67.89 mg, 0.3 mmol, 1 equiv.) was dissolved in toluene (3 ml) and benzyl alcohol **2a** (35.69 mg, 0.33 mmol, 1.1 equiv.) was added and stirred for 14 h at room temperature. Then, methyl acrylate **4d** (38.74 mg, 0.45 mmol, 1.5 equiv.) was added and the reaction was stirred for 24 h at 100 °C. Purification of the crude product by silica gel column chromatography (ethyl acetate in *n*-pentane: 0% to 2% to 5% to 10% to 15% to 20%) yielded *trans*-(±)-**6ad** (37.9 mg, 161.79 µmol, 54%) and *cis*-(±)-**6ad** (9.4 mg, 40.13 µmol, 13%. Both isomers could be obtained as a slightly yellow oil. The *in-situ* ^{1}H-NMR spectrum shows a d.r. of 4.3:1, favouring the *trans*-isomer.

*Note: The NMR-data of trans-(±)-**6ad** is in agreement with the literature.[2] cis-(±)-**6ad** is not literature known.*

Characterization data of compound *trans*-(±)-**6ad**:

^{1}H NMR (600 MHz, $CDCl_3$, 298 K): δ = 7.36 (m, 5H, Ph) 5.13 (m, 2H, *CH₂*Ph) 3.70 (s, 3H, Me) 2.22 (m, 2H, *CH*CO_2Me, *CH*CO_2Bn) 1.47 (m, 2H, CH_2).

^{13}C{^{1}H} NMR (151 MHz, $CDCl_3$, 298 K): δ = 172.2 (*C*O_2Me) 171.7 (*C*O_2Bn) 135.6 (Ph) 128.7 (Ph) 128.5 (Ph) 128.4 (Ph) 67.0 (*CH_2*Ph) 52.3 (Me) 22.5 (*CH*CO_2Me) 22.4 (*CH*CO_2Bn) 15.6 (CH_2).

IR (ATR) ṽ [cm^{-1}] 2958, 1723, 1500, 1450, 1407, 1375, 1327, 1267, 1163, 1118, 1092, 1059, 1020, 932, 897, 847, 749, 698, 664, 601, 578, 508, 453.

HR-MS-ESI (+) calc. $C_{13}H_{14}O_4Na^+$ [M+Na]$^+$ 257.0784; found 257.0780.

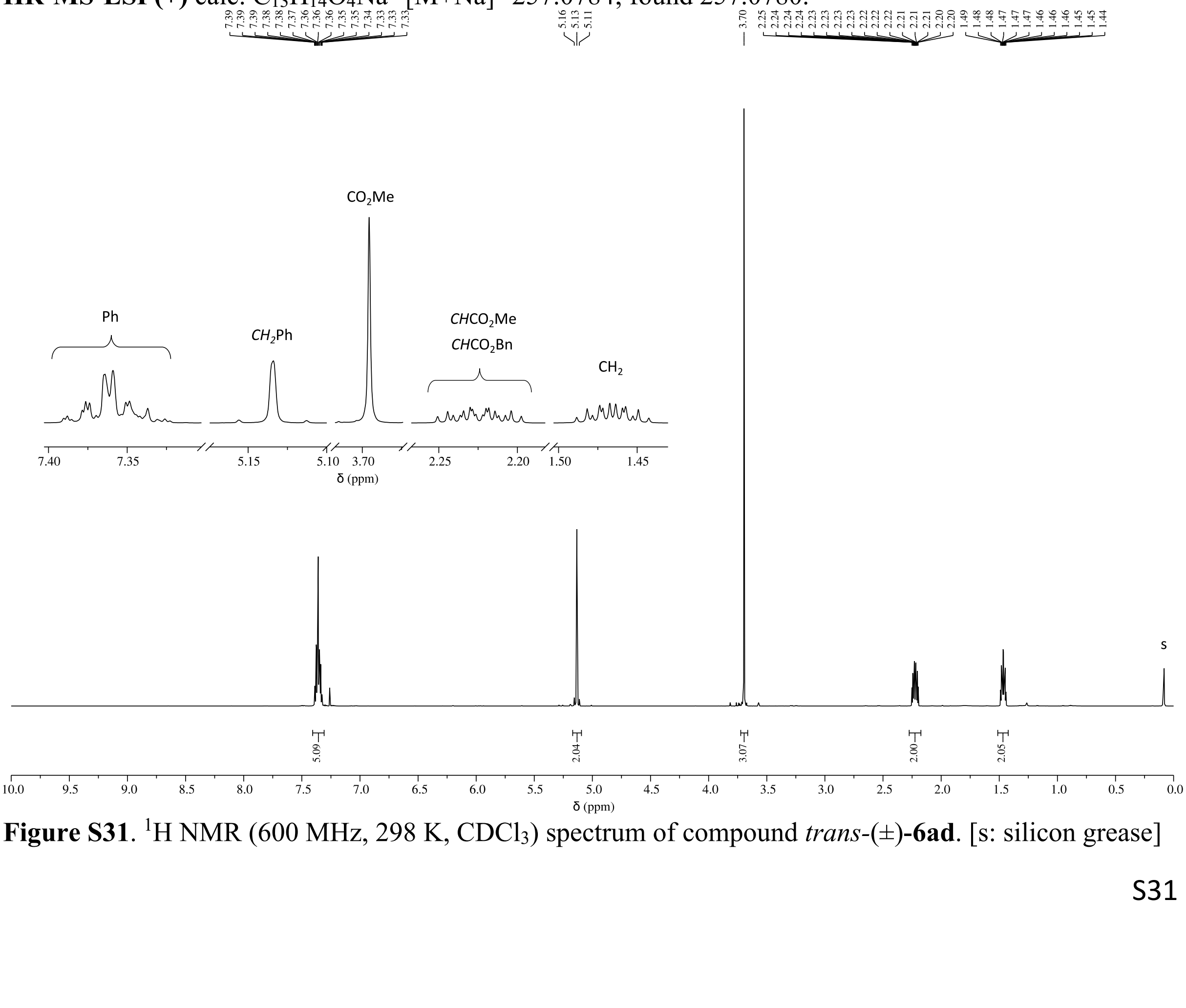

Figure S31. ^{1}H NMR (600 MHz, 298 K, $CDCl_3$) spectrum of compound *trans*-(±)-**6ad**. [s: silicon grease]

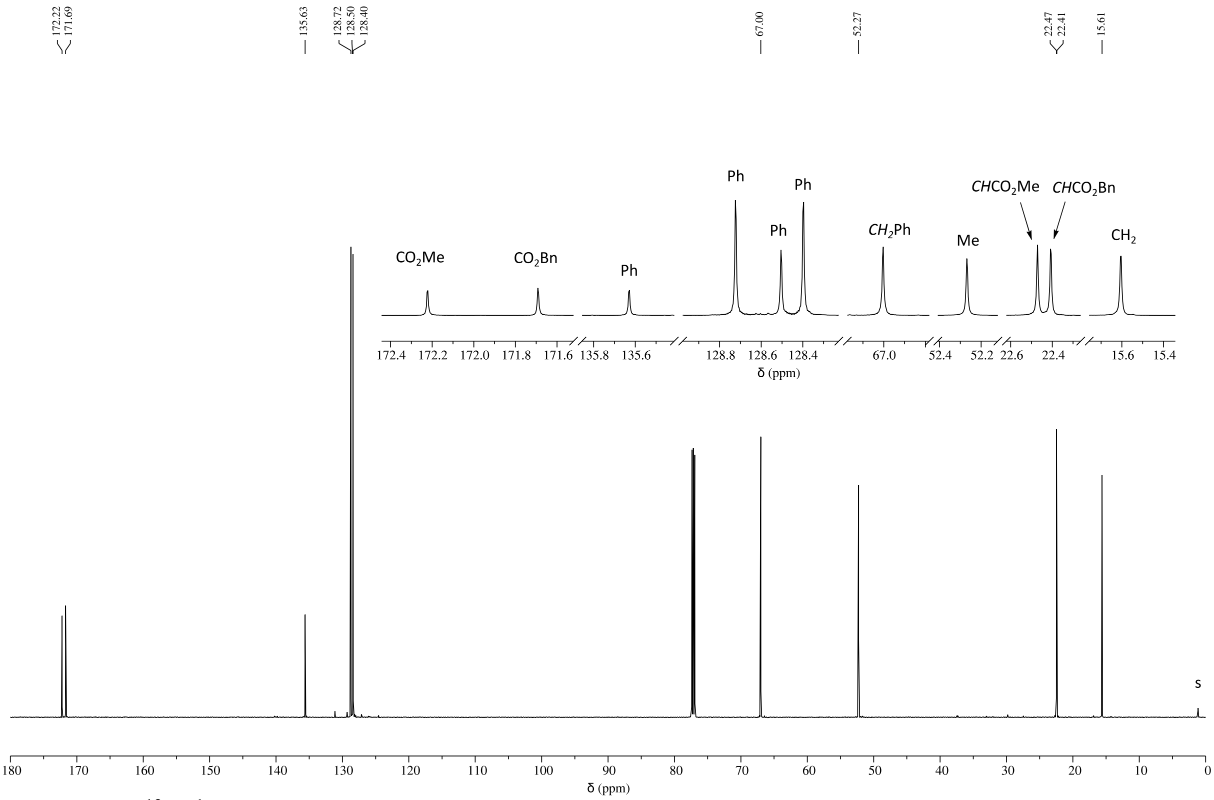

Figure S32.$^{13}C\{^{1}H\}$ NMR (150 MHz, 298 K, $CDCl_3$) spectrum of compound *trans*-(±)-**6ad**. [s: silicon grease]

Characterization data of compound *cis*-(±)-**6ad**:

^{1}H NMR (600 MHz, $CDCl_3$, 298 K): δ = 7.36 (m, 5H, Ph) 5.14 (m, 2H, *CH_2*Ph) 6.60 (s, 3H, Me) 2.09 (m, 2H, *CH*CO_2Me, *CH*CO_2Bn) 1.72 (td, $^{2}J_{HH}$ = 5.0, $^{3}J_{HH}$ = 6.7 Hz, 1H, CH_2) 1.26 (td, $^{2}J_{HH}$ = 5.0, $^{3}J_{HH}$ = 8.5 Hz, 1H, CH_2).
$^{13}C\{^{1}H\}$ NMR (151 MHz, $CDCl_3$, 298 K): δ = 170.4 (*C*O_2Me) 169.9 (*C*O_2Bn) 135.9 (Ph) 128.7 (Ph) 128.6 (Ph) 128.4 (Ph) 67.0 (*CH_2*Ph) 52.2 (Me) [21.8, 21.7] (*CH*CO_2Me, *CH*CO_2Bn) 12.0 (CH_2).
IR (ATR) ṽ [cm^{-1}] 2957, 2855, 1728, 1500, 1440, 1383, 1358, 1263, 1207, 1157, 1107, 1067, 1045, 1029, 972, 908, 805, 737, 698, 602, 459.

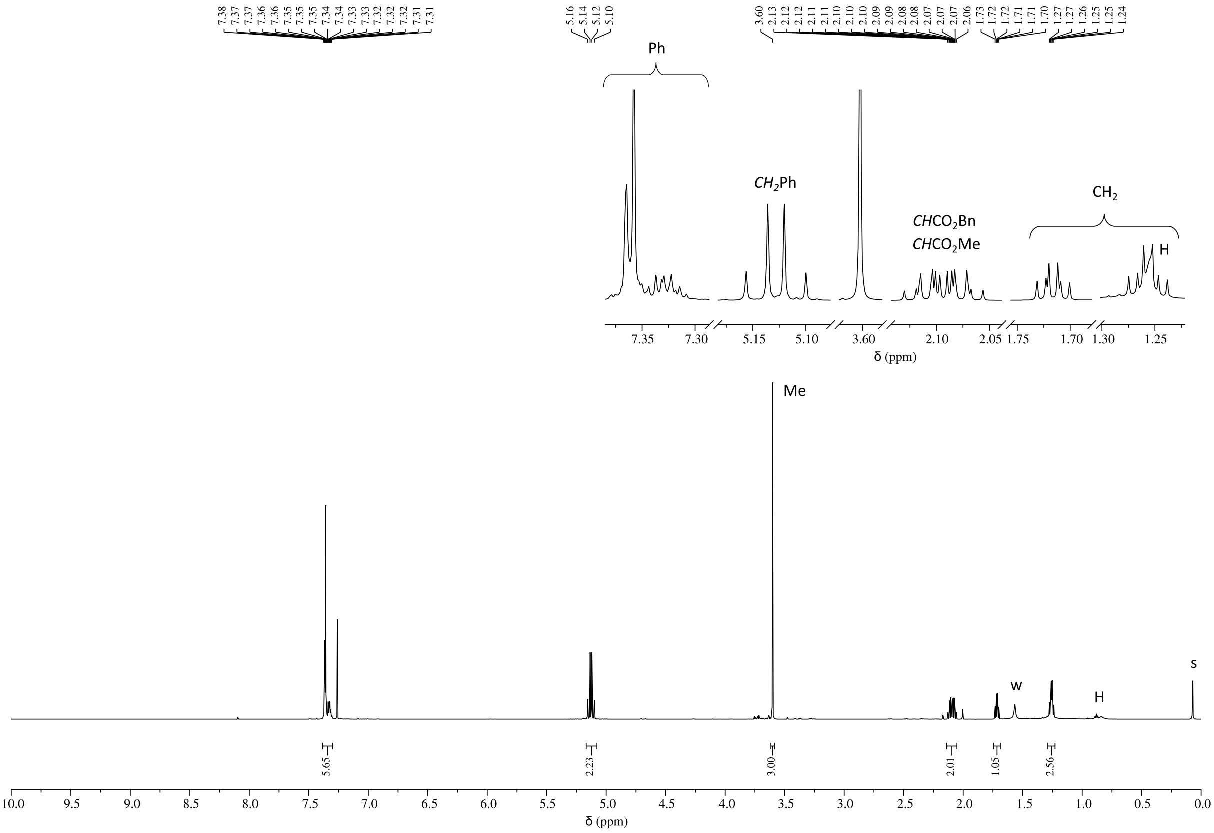

Figure S33. ^{1}H NMR (600 MHz, 298 K, $CDCl_3$) spectrum of compound *cis*-(±)-**6ad**. [w: water; H: H-grease; s: silicon grease]

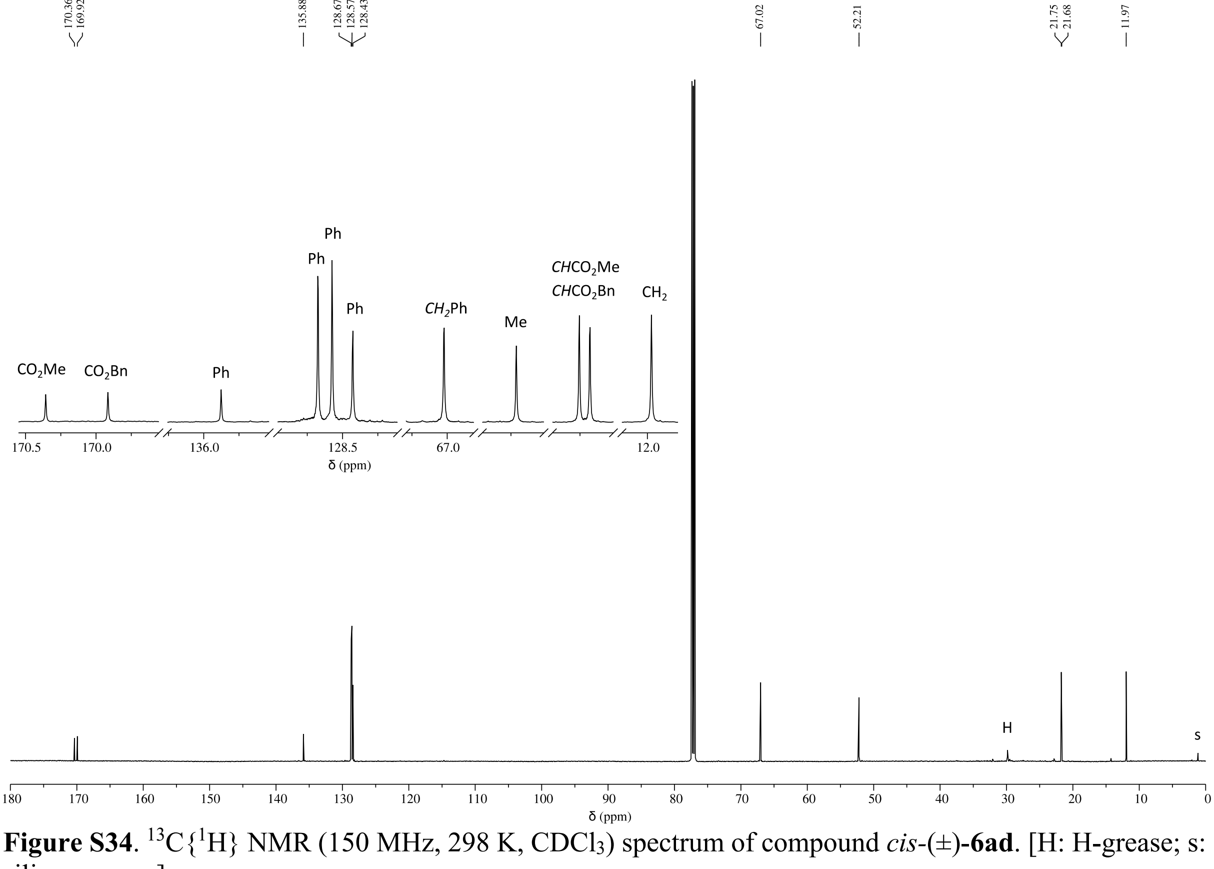

Figure S34. $^{13}C\{^{1}H\}$ NMR (150 MHz, 298 K, $CDCl_3$) spectrum of compound *cis*-(±)-**6ad**. [H: H-grease; s: silicon grease]

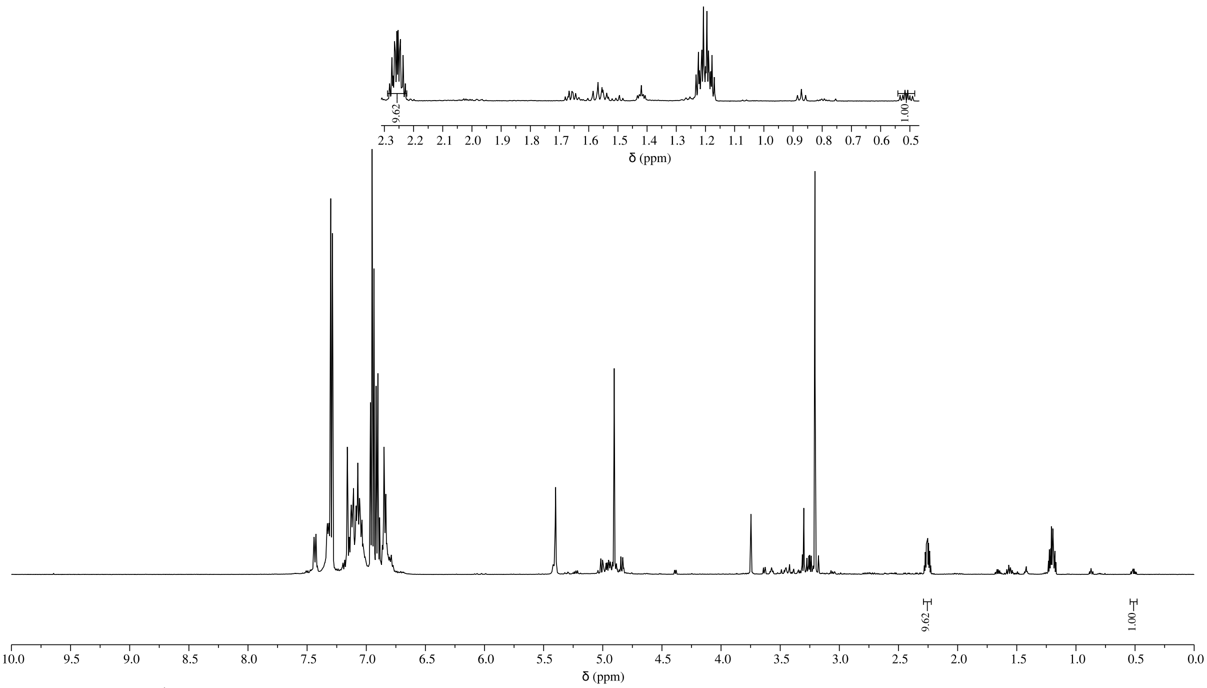

Figure S35. ^{1}H NMR (500 MHz, 298 K, C_6D_6) spectrum of the *in-situ* reaction for the synthesis of compound (±)-**6ad**.

Synthesis of compound (±)-6ae:

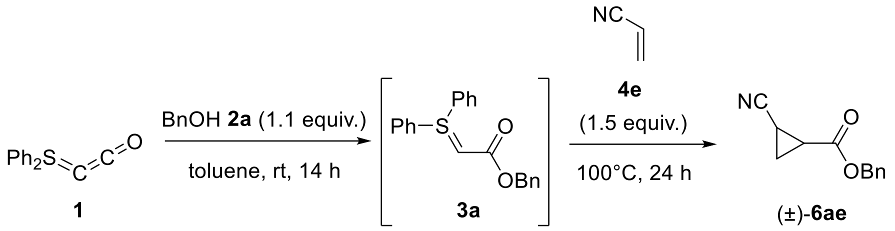

Reagent **1** (67.89 mg, 0.3 mmol, 1 equiv.) was dissolved in toluene (3 ml) and benzyl alcohol **2a** (35.69 mg, 0.33 mmol, 1.1 equiv.) was added and stirred for 14 h at room temperature. Then, acrylonitrile **4e** (23.88 mg, 0.81 g/ml, 29.48 µl, 0.45 mmol, 1.5 equiv.) was added and the reaction was heated up to 100 °C for 24 h. The crude product was purified by silica gel column chromatography (ethyl acetate in pentane: 0% to 2% to 5% to 10% to 15% to 20% to 25% to 30% to 40%) to obtain product *trans*-(±)-**6ae** (25.2 mg, 125.23 µmol, 42%) as a light-yellow oil and compound *cis*-(±)-**6ae** (15.8 mg, 78.52 µmol, 26%) as yellow oil. The *in-situ* ^{1}H-NMR spectrum shows a d.r. of 1.44 :1, favouring the *trans*-isomer.
Both NMR-spectra are in agreement with the literature.[3]

Characterization data of compound *trans*-(±)-**6ae**:

^{1}H NMR (600 MHz, $CDCl_3$, 298 K): δ = 7.38 (m, 5H, Ph) 5.16 (s, 2H, CH_2Ph) 2.31 (ddd, $^{3}J_{HH}$ = 4.3, $^{3}J_{HH}$ = 6.0, $^{3}J_{HH}$ = 8.8 Hz, 1H, *CH*CN) 1.97 (ddd, $^{3}J_{HH}$ = 4.3, $^{3}J_{HH}$ = 6.2, $^{3}J_{HH}$ = 9.3 Hz, 1H, *CH*CO_2Bn) 1.55 (ddd, $^{2}J_{HH}$ = 4.9, $^{3}J_{HH}$ = 6.0, $^{3}J_{HH}$ = 9.2 Hz, 1H, CH_2) 1.50 (ddd, $^{2}J_{HH}$ = 4.9, $^{3}J_{HH}$ = 6.2, $^{3}J_{HH}$ = 8.8 Hz, 1H, CH_2).
^{13}C{^{1}H} NMR (150 MHz, $CDCl_3$, 298 K): δ = 170.2 (*C*O_2Bn) 135.1 (Ph) 128.8 (Ph) 128.8 (Ph) 128.6 (Ph) 119.2 (CN) 67.7 (*CH_2*Ph) 21.2 (*CH*CN) 14.8 (*CH*CO_2Bn) 6.0 (CH_2).
IR (ATR) ṽ [cm^{-1}] 2248, 1730, 1500, 1457, 1409, 1372, 1308, 1270, 1200, 1173, 1126, 1055, 1005, 909, 867, 808, 739, 698, 601, 581, 537, 498.

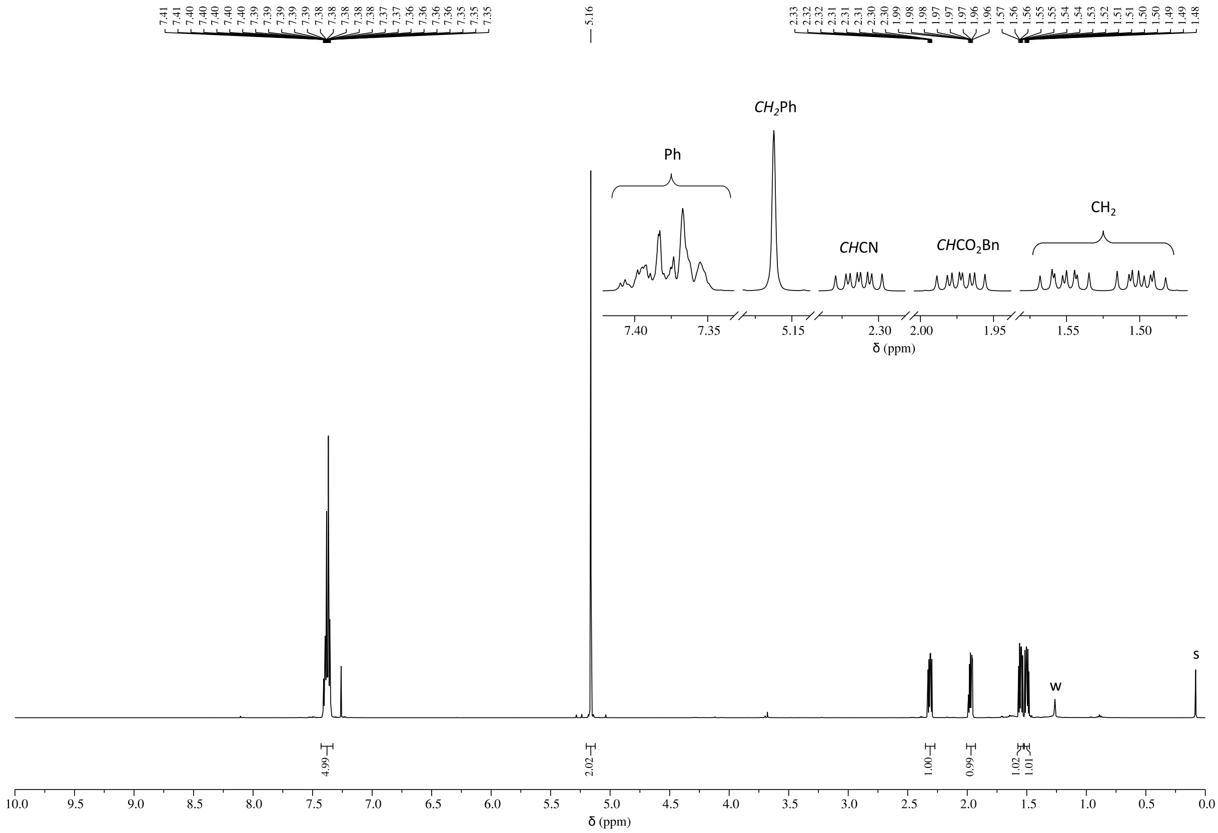

Figure S36. ^{1}H NMR (600 MHz, 298 K, $CDCl_3$) spectrum of compound *trans*-(±)-**6ae**. [w: water; s: silicon grease]

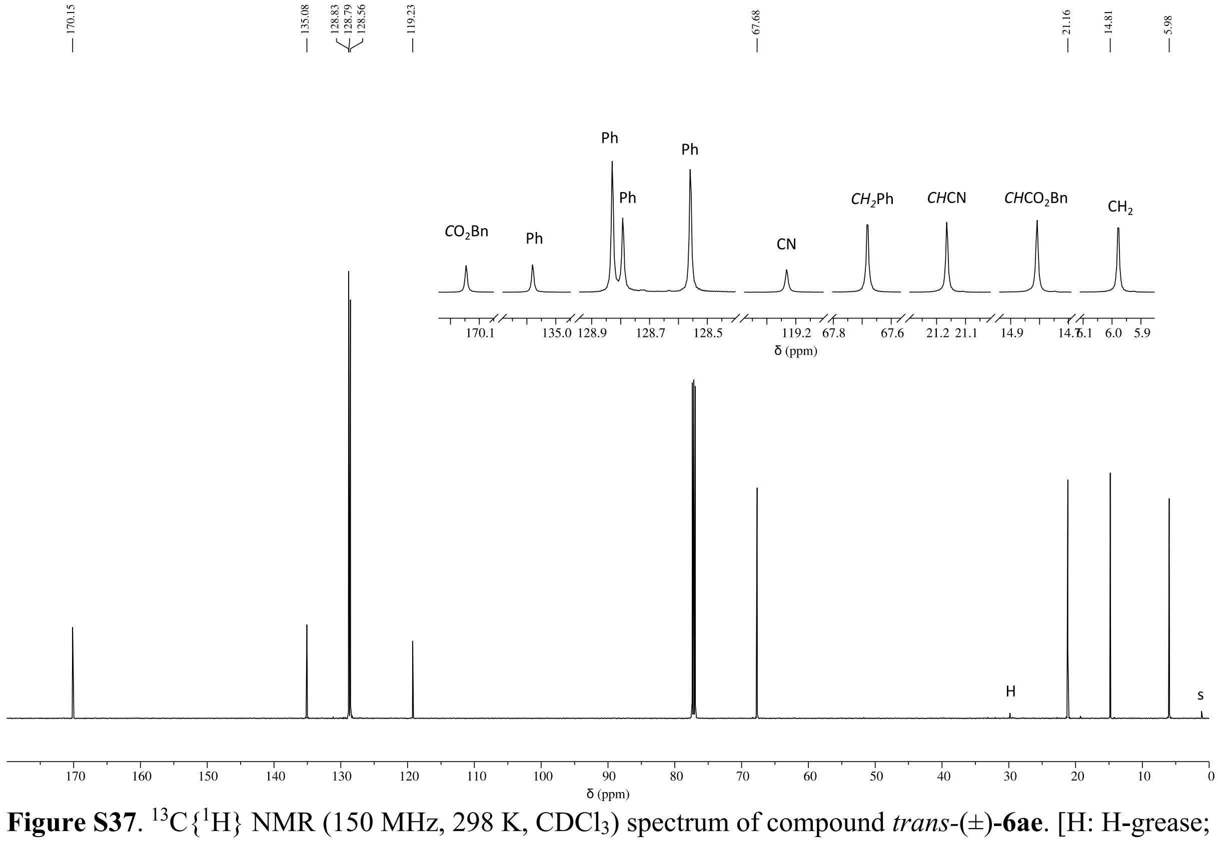

Figure S37. $^{13}C\{^1H\}$ NMR (150 MHz, 298 K, $CDCl_3$) spectrum of compound *trans*-(±)-**6ae**. [H: H-grease; s: silicon grease]

Characterization data of compound *cis*-(±)-**6ae**:

^{1}H NMR (600 MHz, $CDCl_3$, 298 K): δ = 7.38 (m, 5H, Ph) 5.32 (s, 2H, CH_2Ph) 2.17 (m, 1H, CHCN) 1.86 (ddd, $^{3}J_{HH}$ = 6.8, $^{3}J_{HH}$ = 8.1, $^{3}J_{HH}$ = 9.0 Hz, 1H, $CHCO_2Bn$) 1.71 (m, 1H, CH_2) 1.43 (ddd, $^{2}J_{HH}$ = 5.1, $^{3}J_{HH}$ = 8.1, $^{3}J_{HH}$ = 9.0 Hz, 1H, CH_2).
^{13}C{^{1}H} NMR (151 MHz, $CDCl_3$, 298 K): δ = 168.9 ($\mathit{C}O_2Bn$) 135.2 (Ph) 128.8 (Ph) 128.7 (Ph) 117.6 (CN) 67.7 (*CH_2*Ph) 20.1 (*CH*CN) 13.6 (CH_2) 6.0 (*CH*CO_2Bn).
IR (ATR) ṽ [cm^{-1}] 2248, 1732, 1500, 1457, 1402, 1365, 1275, 1172, 1126, 1043, 944, 897, 843, 781, 751, 698, 604, 580, 501.

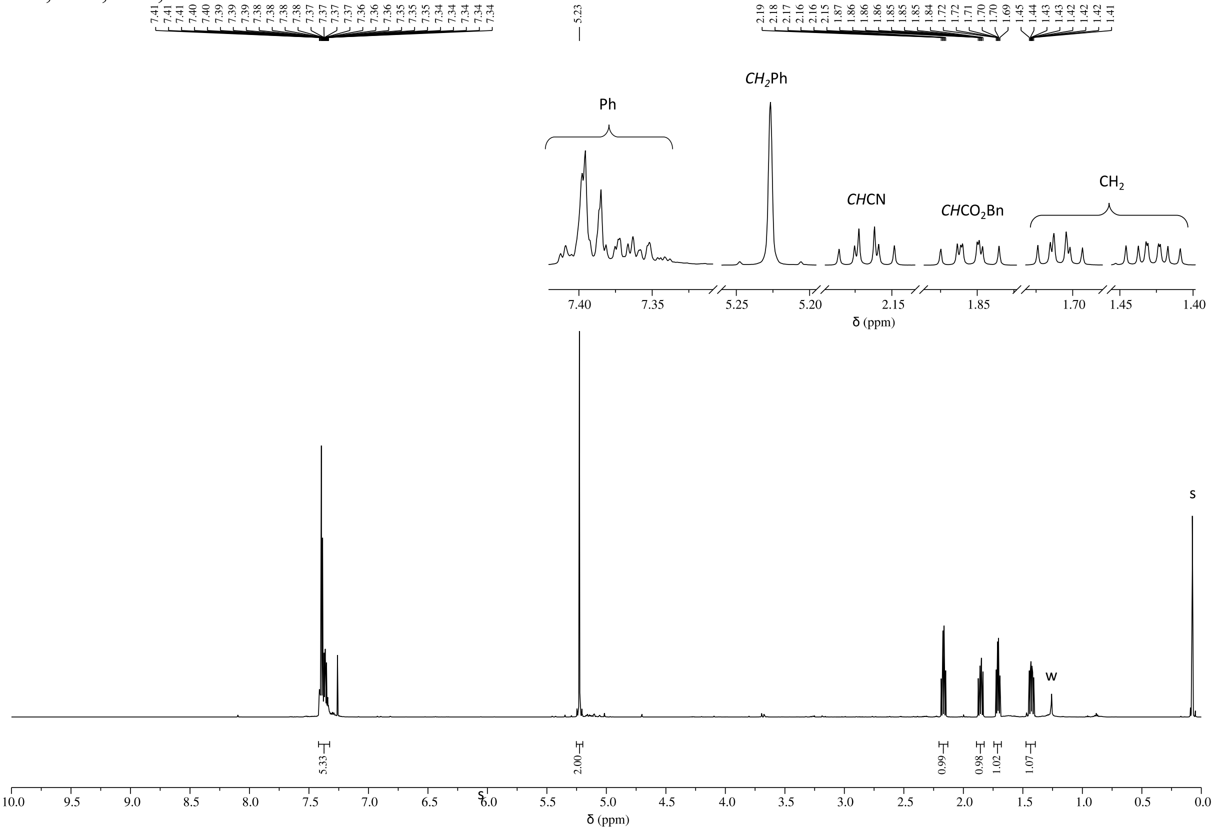

Figure S38. ^{1}H NMR (600 MHz, 298 K, $CDCl_3$) spectrum of compound *cis*-(±)-**6ae**. [w: water; s: silicon grease]

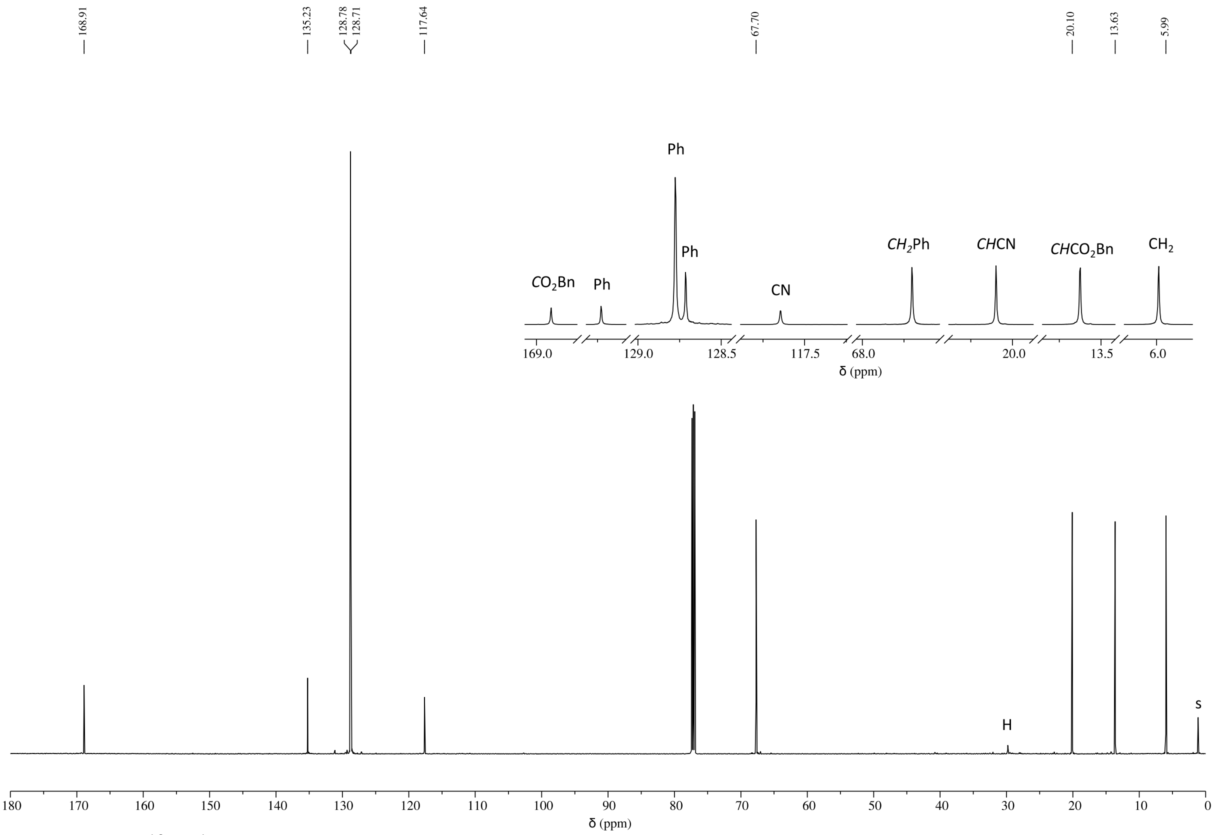

Figure S39. $^{13}C\{^1H\}$ NMR (150 MHz, 298 K, $CDCl_3$) spectrum of compound *cis*-(±)-**6ae**. [H: H-grease; s: silicon grease]

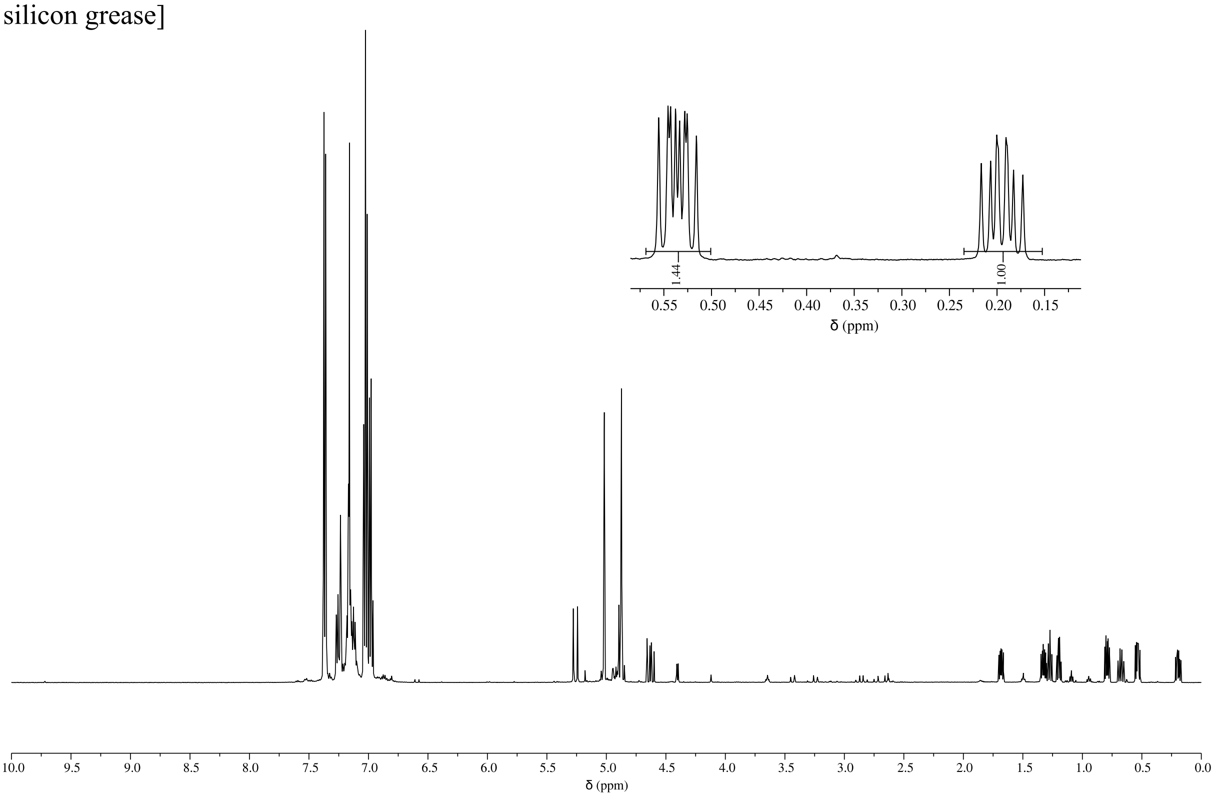

Figure S40. ^1H NMR (500 MHz, 298 K, C_6D_6) spectrum of the *in situ* reaction to synthesis compound (±)-**6ae**.

Synthesis of compound (±)-6af:

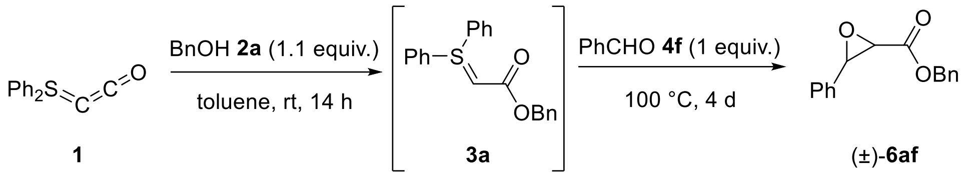

Reagent **1** (67.89 mg, 0.3 mmol, 1 equiv.) was dissolved in toluene (5 ml) and benzyl alcohol **2a** (35.69 mg, 0.33 mmol, 1.1 equiv.) was added and stirred for 14 h at room temperature. Then, benzaldehyde **4f** (31.84 mg, 0.3 mmol, 1 equiv.) was added and the reaction was heated to 100 °C for 4 days. Purification by silica gel column chromatography (ethyl acetate in pentane: 0% to 2% to 6% to 10% to 15%) yielded the mixture of the *cis*- and *trans*- isomers with 54% (41.5 mg) in total. The *in situ* ^{1}H-NMR spectrum shows an d.r. of 12:1, favouring the *trans*-isomer.

Characterization data of compound mixture of compounds *trans*-(±)-**6af** and *cis*-(±)-**6af**:

^{1}H NMR (500 MHz, C_6D_6, 298 K): δ = 7.34 (m, 2H^{cis}, Ph) 7.14 – 6.90 (m, 10H^{trans}+6H^{cis}, Ph) 6.82 (m, 2H^{cis}, Ph) 4.95 (m, 2H^{trans}, CH_2) 4.68 (m, 2H^{cis}, CH_2) 4.02 (d, $^{3}J_{HH}$ = 1.54 Hz, 1H^{trans}, *CH*Ph) 3.69 (d, $^{3}J_{HH}$ = 4.55 Hz, 1H^{cis}, *CH*Ph) 3.42 (d, $^{3}J_{HH}$ = 4.58 Hz, 1H^{cis}, *CH*CO_2Bn) 3.31 (d, $^{3}J_{HH}$ = 1.67 Hz, 1H^{trans}, *CH*CO_2Bn).
^{13}C{^{1}H} NMR (125 MHz, C_6D_6, 298 K): δ = 167.8 (*C*O_2Bn^{trans}) 166.2 (*C*O_2Bn^{cis}) 135.8 (Ph^{trans}) 135.6 (Ph^{trans}) 133.6 (Ph^{cis}) 128.9–127.2 (Ph^{trans}+Ph^{cis}) 128.9 (Ph^{trans}) 128.8 (Ph^{trans}) 128.8 (Ph^{trans}) 128.7 (Ph^{trans}) 128.6 (Ph^{trans}) 127.15 (Ph^{cis}) 126.1 (Ph^{trans}) 67.2 (CH_2^{trans}) 66.6 (CH_2^{cis}) 58.0 (*CH*Ph^{trans}) 57.5 (*CH*Ph^{cis}) 57.0 (*CH*CO_2Bn^{trans}) 56.1 (*CH*CO_2Bn^{cis}).
IR (ATR) ṽ [cm^{-1}] 3035, 1746, 1497, 1457, 1422, 1378, 1340, 1277, 1242, 1186, 1109, 1082, 1001, 892, 815, 734, 693, 600, 521.
HR-MS-ESI (+) calc. $C_{16}H_{14}O_3Na^+$ $[M+Na]^+$ 277.0835; found 277.0829.

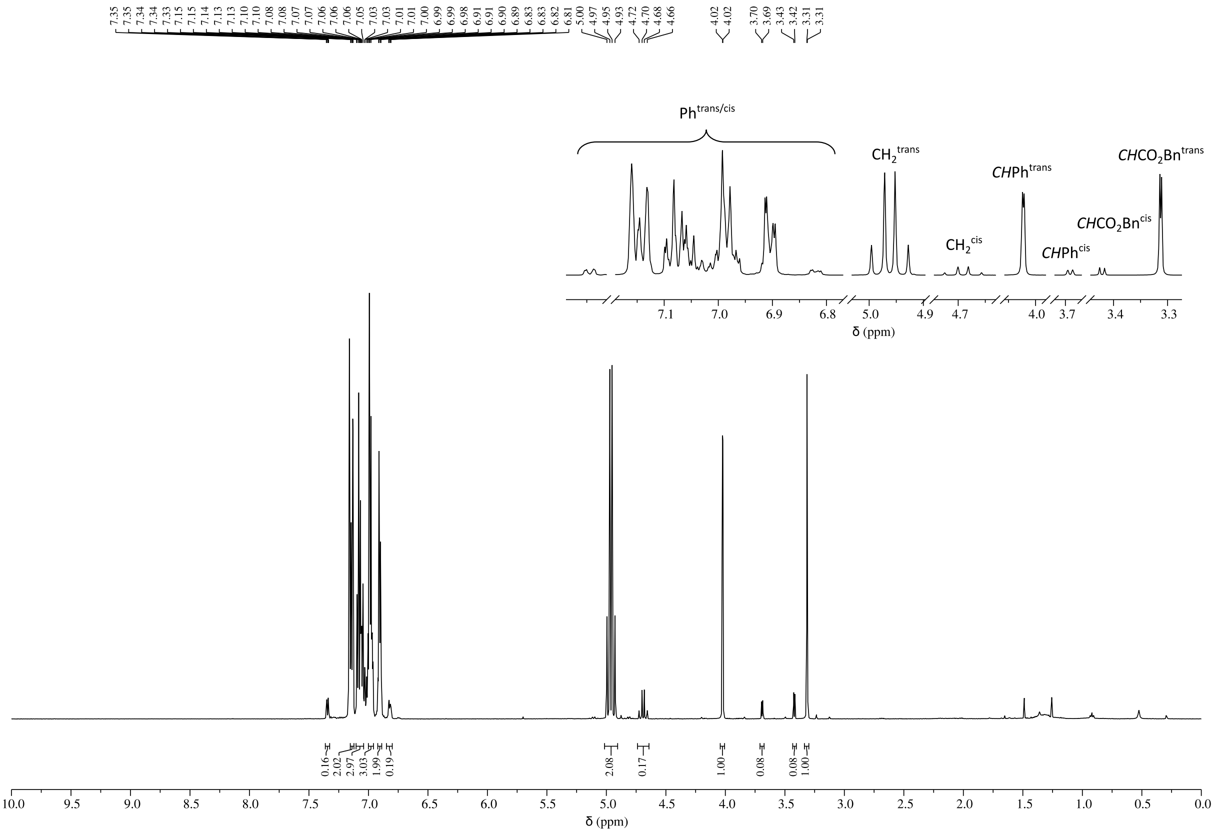

Figure S41. ^{1}H NMR (500 MHz, 298 K, C_6D_6) spectrum of compound mixture of *trans*-(±)-**6af** and *cis*-(±)-**6af**.

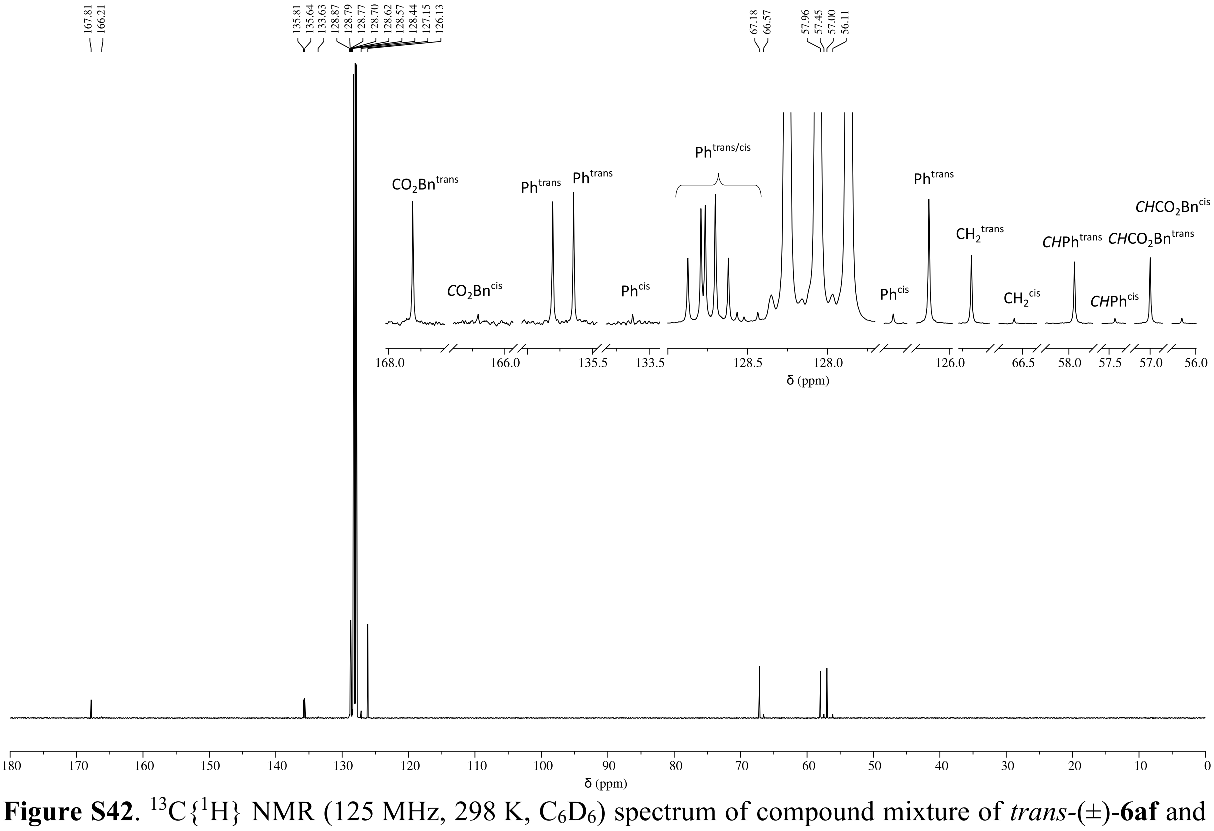

Figure S42. $^{13}C\{^1H\}$ NMR (125 MHz, 298 K, C_6D_6) spectrum of compound mixture of *trans*-(±)-**6af** and *cis*-(±)-**6af**.

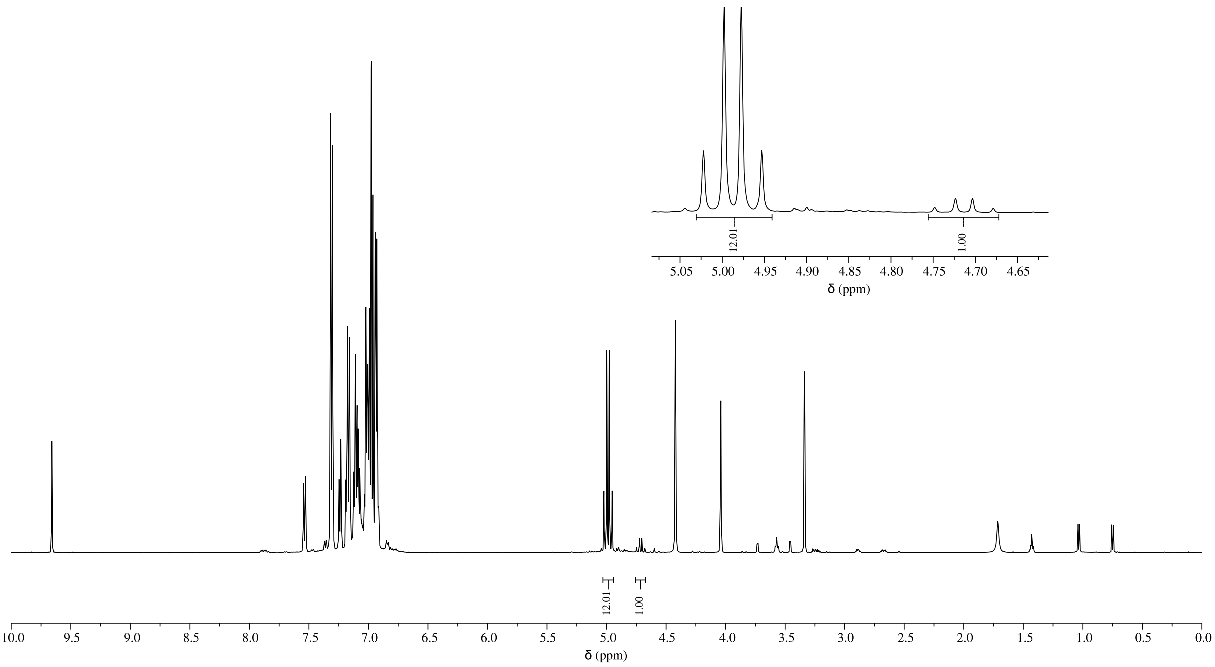

Figure S43. ^{1}H NMR (500 MHz, 298 K, C_6D_6) spectrum of the *in-situ* reaction to synthesis compounds (±)-**6af**.

Synthesis of compound (±)-6ag:

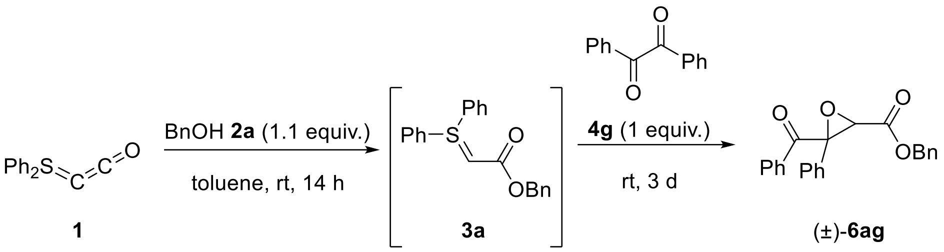

Reagent **1** (67.89 mg, 0.3 mmol, 1 equiv.) was dissolved in toluene (3 ml) and benzyl alkohol **2a** (35.69 mg, 0.33 mmol, 1.1 equiv.) was added and stirred for 14 h at room temperature. Then, benzil **4g** (63.07 mg, 0.3 mmol, 1 equiv.) was added and the reaction was stirred for 3 days at room temperature. The crude product was purified by silica gel column chromatography (ethyl acetate in pentane: 0% to 2% to 4% to 6% to 8%) to obtain the mixture of compounds (±)-**6ag** (89.2 mg, 0.249 mmol, 83%, d.r. = 1.5:1) as colourless sticky oil. The *in situ* ^{1}H-NMR spectrum shows a d.r. of 1.5:1.

Characterization data of compound mixture of *major*-(±)-**6ag** and *minor´*-(±)-**6ag**:

^{1}H NMR (600 MHz, $CDCl_3$, 298 K): δ = 8.07 (m, 2H, Ph) 8.02 (m, 2H´, Ph) 7.61 (m, 2H, Ph) 7.54 (m, 2H + 2H´, Ph) 7.41 (m, 2H + 2H´, Ph) 7.38 (m, 4H´, Ph) 7.30 (m, 5H + 5H´, Ph) 7.10 (m, 2H, Ph) 5.17 (m, 2H´, CH_2) 5.00 (m, 2H, CH_2) 4.16 (s, 1H, CH) 3.77 (s, 1H´, CH).
$^{13}C\{^1H\}$ NMR (150 MHz, $CDCl_3$, 298 K): δ =192.6 (C(O)Ph) 192.0 (C´(O)Ph) 166.3 (C´O_2Bn) 165.5 (CO_2Bn) [134.9, 134.7, 134.5, 133.6, 133.4, 130.8] (Ph-C/Ph-C`) 134.1 (Ph) 134.0 (Ph´) 130.0 (Ph) 129.8 (Ph´) [129.4, 129.14, 129.05] (Ph/Ph´) [128.69, 128.67, 128.65, 128.59, 128.57, 128.55, 128.5, 128.43] (Ph/Ph´) 128.40 (Ph) 126.9 (Ph) 125.6 (Ph´) 68.4 (C) 67.8 (C´) 67.7 (CH_2´) 67.2 (CH_2) 60.9 (CH´) 58.5 (CH).
IR (ATR) ṽ [cm^{-1}] 1756, 1683, 1599, 1582, 1499, 1450, 1415, 1263, 1193, 1179, 1079, 1003, 910, 868, 798, 734, 694, 633, 587, 502.
HR-MS-ESI (+) calc. $C_{23}H_{18}O_4Na^+$ $[M+Na]^+$ 381.1097; found 381.1086.

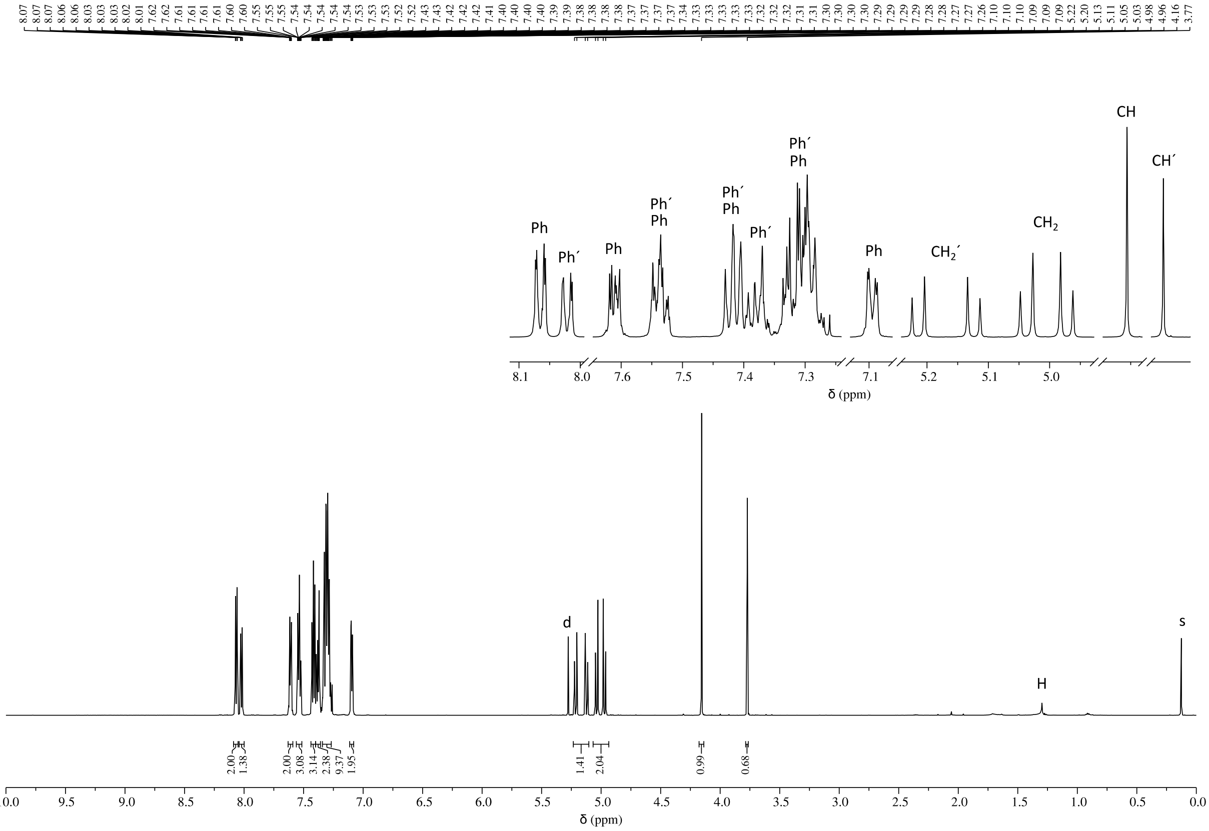

Figure S44. ^{1}H NMR (600 MHz, 298 K, $CDCl_3$) spectrum of compound mixture of *major*-(±)-**6ag** and *minor´*-(±)-**6ag**. [d: dichloromethane H: H-grease; s: silicon grease]

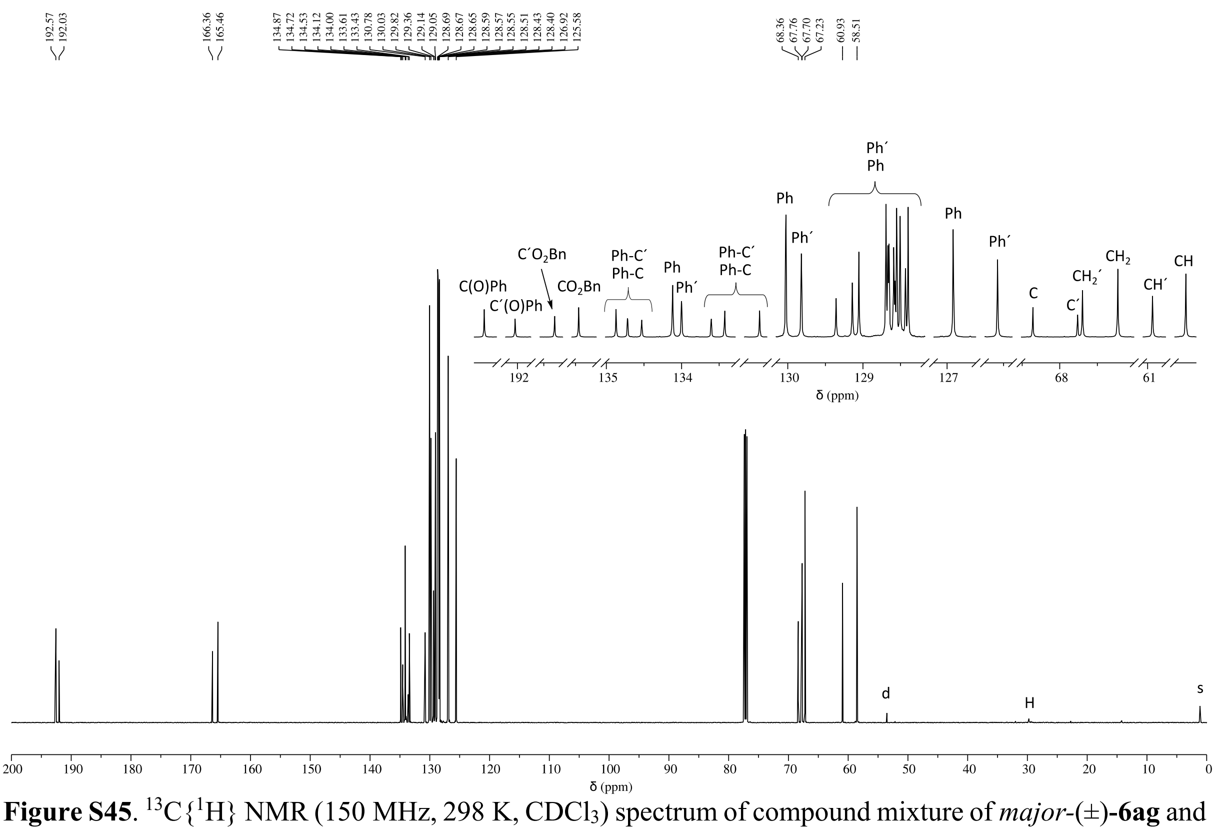

Figure S45. ^{13}C{^{1}H} NMR (150 MHz, 298 K, $CDCl_3$) spectrum of compound mixture of *major*-(±)-**6ag** and *minor´*-(±)-**6ag**. [d: dichloromethane H: H-grease; s: silicon grease]

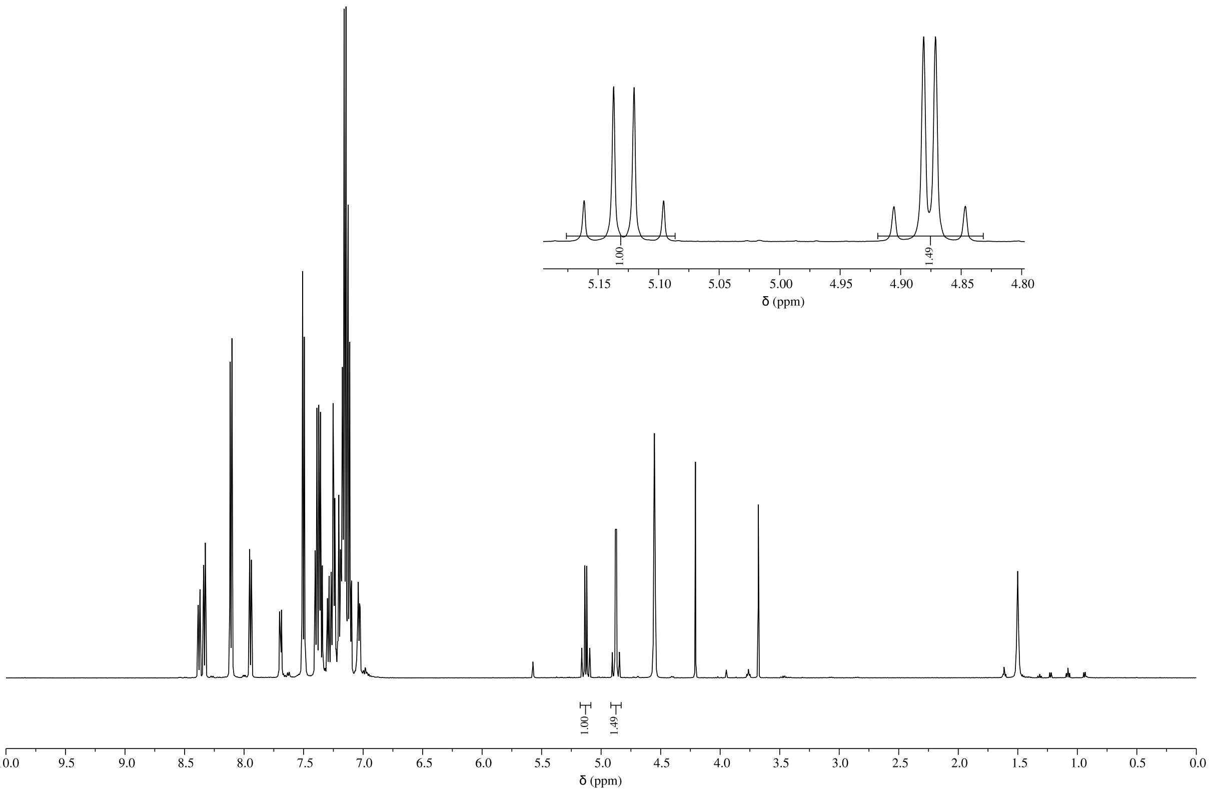

Figure S46. ^{1}H NMR (500 MHz, 298 K, C_6D_6) spectrum of the *in-situ* reaction to synthesis compound (±)-**6ag**.

Synthesis of compound 7aa:

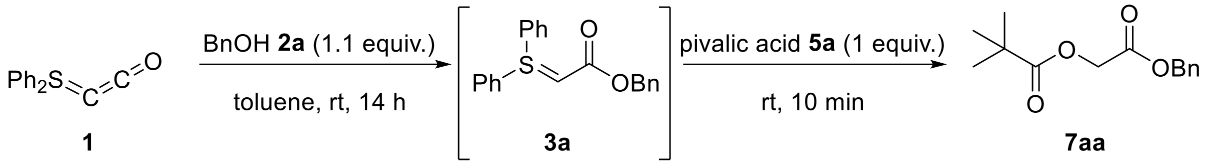

Reagent **1** (67.89 mg, 0.3 mmol, 1 equiv.) was dissolved in toluene (3 ml) and benzyl alcohol **2a** (35.69 mg, 0.33 mmol, 1.1 equiv.) was added and stirred at room temperature. Then, pivalic acid **5a** (30.64 mg, 0.3 mmol, 1 equiv.) was added and stirred for 10 min at room temperature. The crude product was purified by silica gel column chromatography (ethyl acetate in pentane: 0% to 2% to 5%) to obtain product **7aa** (64 mg, 0.26 mmol, 85%) as a colourless oil.

Characterization data of compound **7aa**:

^{1}H NMR (500 MHz, $CDCl_3$, 298 K): δ = 7.35 (m, 5H, Ph) 5.19 (s, 2H, *CH_2*Ph) 4.65 (s, 2H, CH_2) 1.24 (s, 9H, ^{t}Bu).
^{13}C{^{1}H} NMR (125 MHz, $CDCl_3$, 298 K): δ = 178.0 ($CO_2{}^t$Bu) 168.0 (CO_2Bn) 135.3 (Ph) 128.8 (Ph) 128.7 (Ph) 128.6 (Ph) 67.2 (*CH_2*Ph) 60.8 (CH_2) 38.8 (^{t}Bu) 27.2 (^{t}Bu).
IR (ATR) ṽ [cm^{-1}] 2977, 1739, 1482, 1459, 1423, 1399, 1366, 1288, 1211, 1138, 1060, 1030, 952, 861, 770, 753, 697, 491.
HR-MS-ESI (+) calc. $C_{16}H_{14}O_3Na^+$ [M+Na]$^+$ 273.1097; found 273.1096.

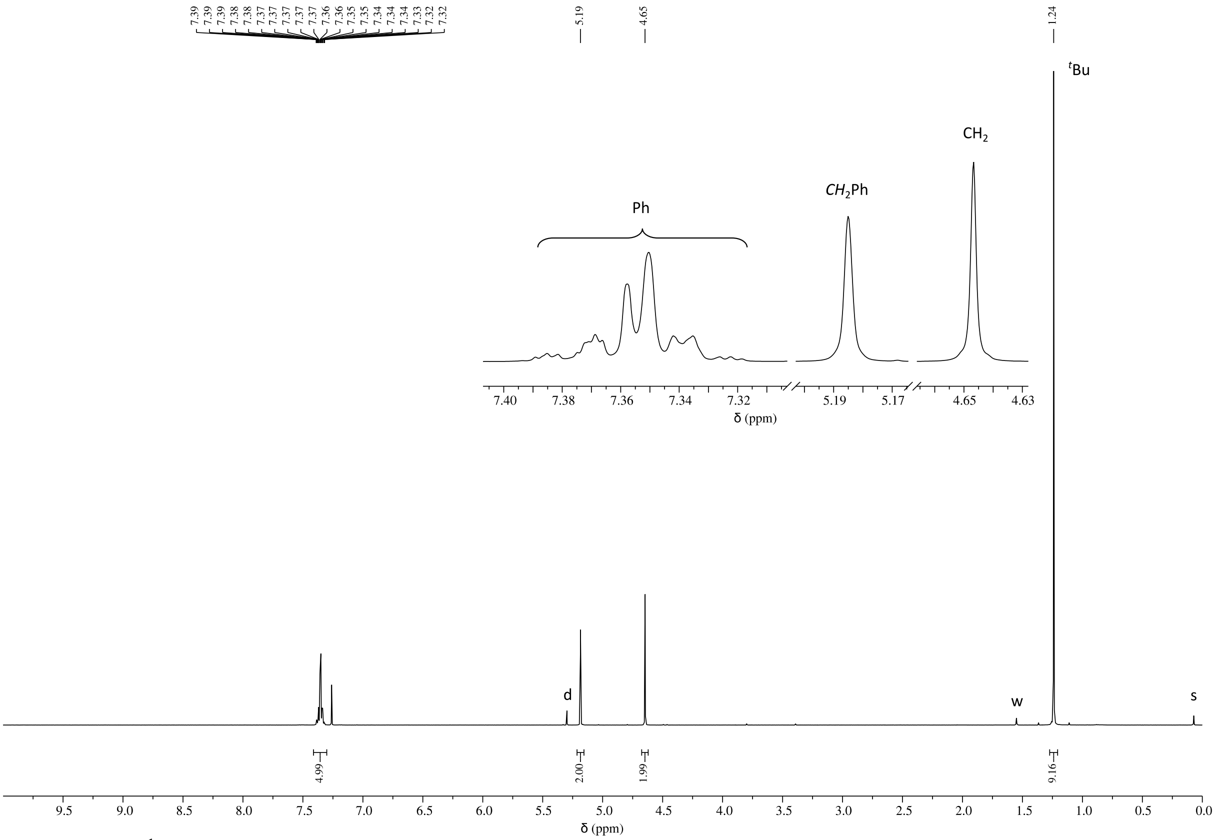

Figure S47. ^{1}H NMR (500 MHz, 298 K, $CDCl_3$) spectrum of compound **7aa**. [d: dichlormethane; w: water, s: silicon grease]

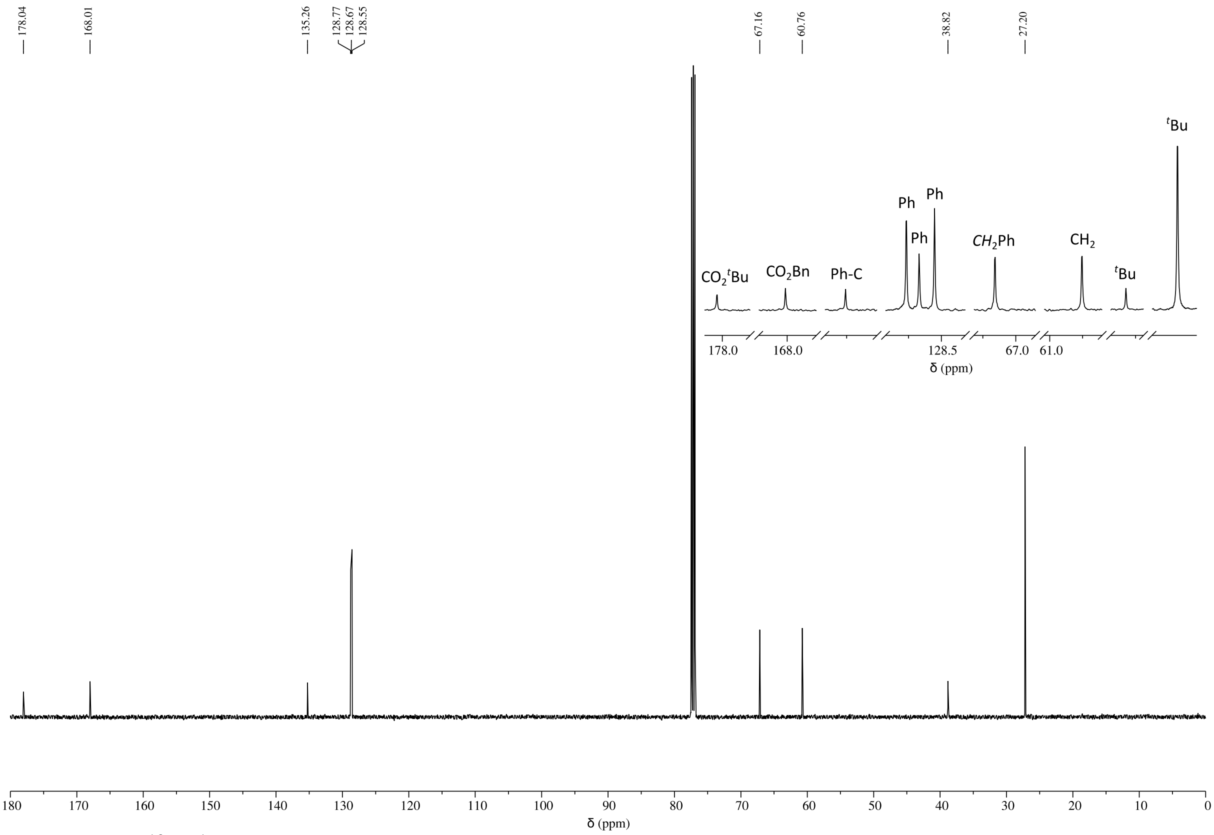

Figure S48. $^{13}C\{^1H\}$ NMR (125 MHz, 298 K, $CDCl_3$) spectrum of compound **7aa**.

Synthesis of compound 7ab:

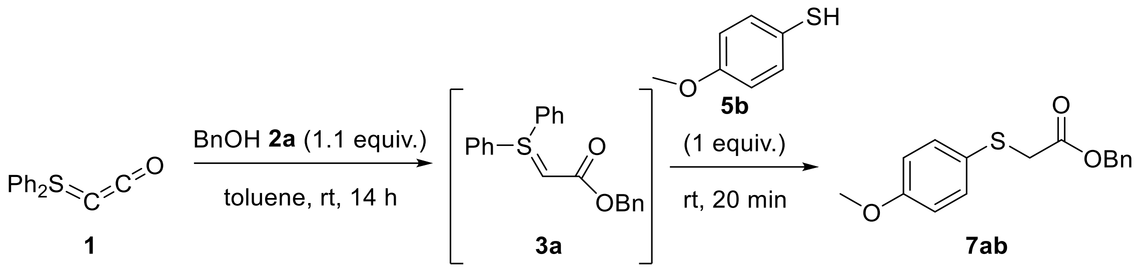

Reagent **1** (67.89 mg, 0.3 mmol, 1 equiv.) was dissolved in toluene (3 ml) and benzyl alcohol **2a** (35.69 mg, 0.33 mmol, 1.1 equiv.) was added and stirred at room temperature. Then, thiol **5b** (42.06 mg, 1.14 g/ml, 36.89 ml, 0.3 mmol, 1 equiv.) was added and stirred for 20 min at room temperature. The crude product was purified by silica gel column chromatography (ethyl acetate in pentane: 0% to 2% to 4% to 6% to 8%) to obtain product **7ab** (66.3 mg, 0.23 mmol, 77%) as a colourless oil.

Characterization data of compound **7ab**:

^{1}H NMR (501 MHz, $CDCl_3$, 298 K): δ = 7.37 (m, 2H, *p*Ar) 7.34 (m, 3H, Ph) 7.28 (m, 2H, Ph) 6.80 (m, 2H, *p*Ar) 5.12 (s, 2H, *CH₂*Ph) 3.79 (s, 3H, OMe) 3.55 (s, 2H, CH_2).
^{13}C{^{1}H} NMR (126 MHz, $CDCl_3$, 298 K): δ = 169.9 (CO_2Bn) 159.8 (*p*Ar-*C*-OMe) 135.6 (Ph-C) 134.5 (*p*Ar) 128.7 (Ph) 128.48 (Ph) 128.46 (Ph) 124.84 (*p*Ar-C) 114.80 (*p*Ar) 67.18 (CH_2Ph) 55.4 (OMe) 38.77 (CH_2).
IR (ATR) ṽ [cm^{-1}] 2840, 1731, 1593, 1573, 1495, 1457, 1408, 1376, 1270, 1244, 1175, 1123, 1029, 972, 891, 826, 800, 738, 697, 639, 626, 575, 524, 495.
HR-MS-ESI (+) calc. $C_{16}H_{14}O_3Na^+$ $[M+Na]^+$ 311.0712; found 311.0716.

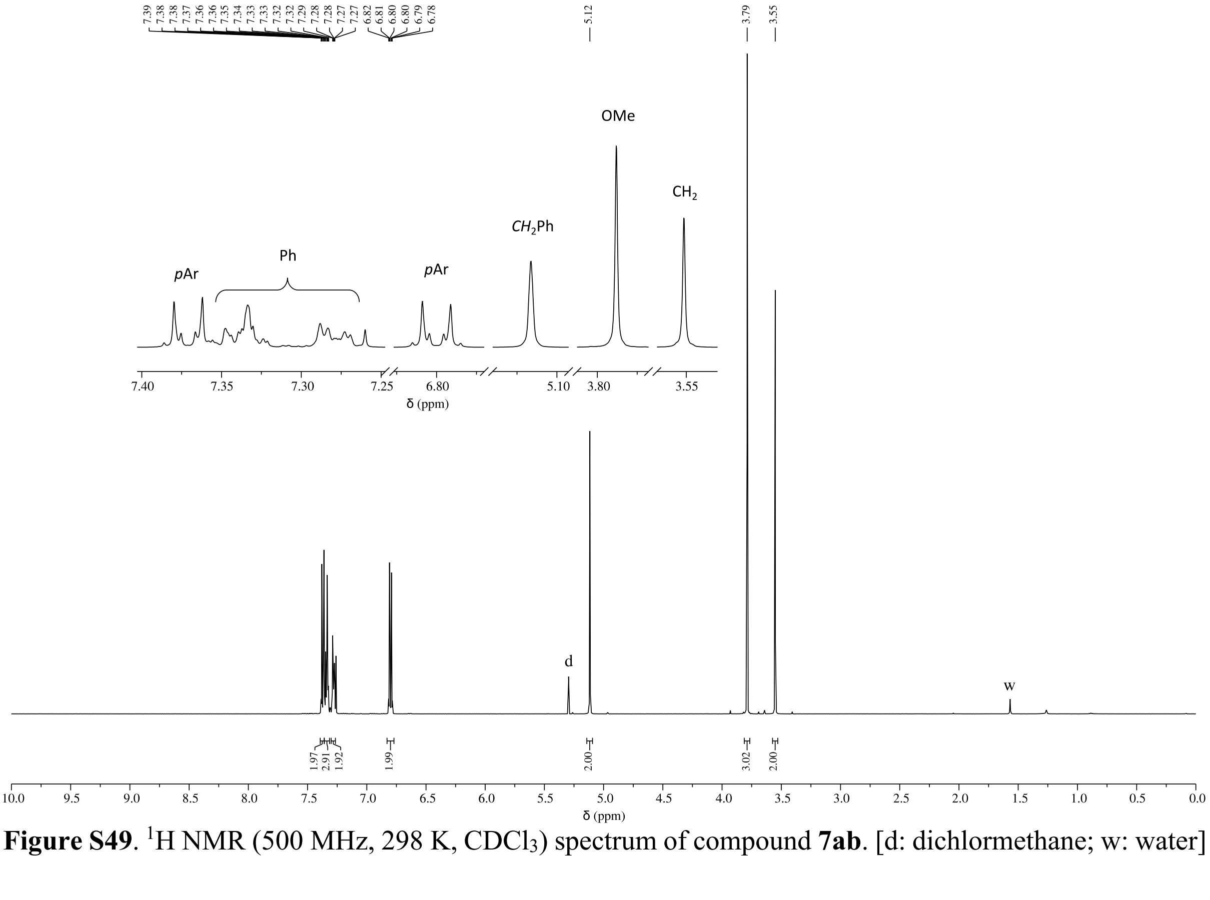

Figure S49. ^{1}H NMR (500 MHz, 298 K, $CDCl_3$) spectrum of compound **7ab**. [d: dichlormethane; w: water]

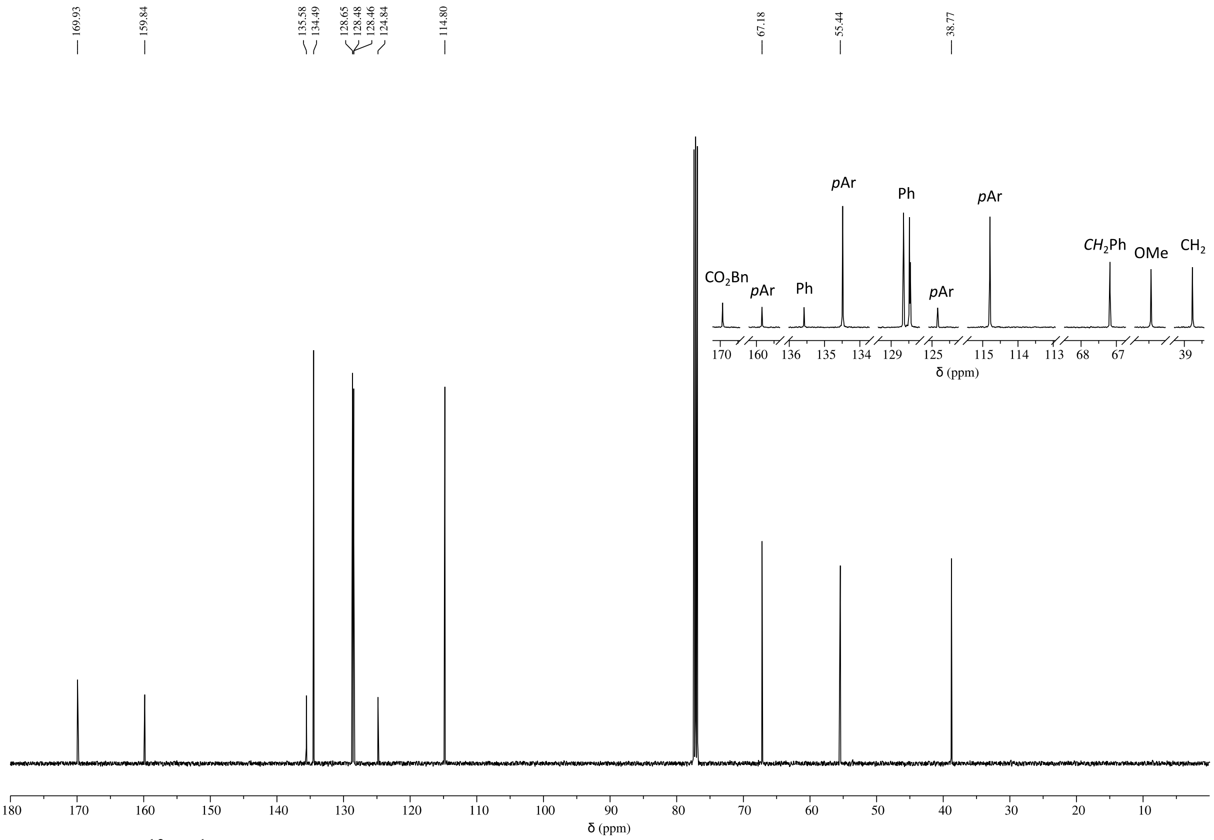

Figure S50. $^{13}C\{^{1}H\}$ NMR (125 MHz, 298 K, $CDCl_3$) spectrum of compound **7ab**.

Synthesis of compound (±)-10a

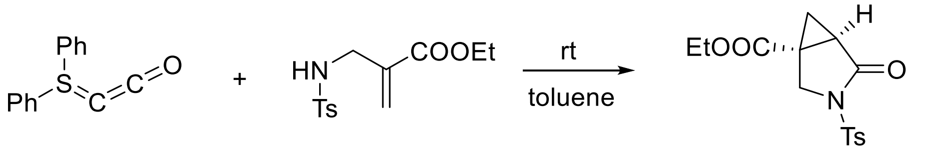

1 **8a** (±)-**10a**

A solution of compound **8a** (56.6 mg, 0.2 mmol) in toluene (1.0 mL) was added dropwise to a solution of reagent **1** (45.2 mg, 0.2 mmol) in toluene (0.5 mL) and stirred at room temperature for 15 min. All the volatiles were removed under reduced pressure, the remaining residue was purified by silica gel column chromatography (eluent: *n*-pentane/EtOAc = 2:1) to give compounds (±)-**10a** (49.7 mg, 0.154 mmol, 77%) as a colorless solid.

Characterization data of compound (±)-**10a**:

m.p. 99 °C.
^{1}H NMR (500 MHz, 298 K, C_6D_6): δ = 8.03 (m, 2H, *o*-C_6H_4), 6.72 (m, 2H, *m*-C_6H_4), [4.12, 3.81](each m, each 1H, NCH_2), 3.67 (m, 2H, CH_2^{Et}), [1.79, 1.20](each m, each 1H, CH_2), 1.79 (s, 3H, CH_3), 0.74 (t, $^3J_{HH}$ = 7.1 Hz, 3H, CH_3^{Et}), 0.38 (t, $^3J_{HH}$ = 4.6 Hz, 1H, CH).
^{13}C{^{1}H} NMR (125 MHz, 298 K, C_6D_6): δ = 169.0 (OC=O), 168.6 (C=O), 144.8 (*p*-C_6H_4), 135.9 (*i*-C_6H_4), 129.6 (*m*-C_6H_4), 128.7 (*o*-C_6H_4), 61.1 (CH_2^{Et}), 47.8 (NCH_2), 29.8 (CH), 25.0 (C), 21.1 (Me), 18.8 (CH_2), 13.8 (CH_3^{Et}).
IR (ATR) [cm^{-1}]: ṽ = 2988, 2926, 1733, 1599, 1479, 1449, 1404, 1366, 1294.99, 1248.48, 1211, 1171, 1130, 1092, 1067, 1029, 964, 929, 904, 874, 814, 754, 735, 694, 668, 605, 575, 550, 502, 435.
HR-MS-ESI(+) calc. $C_{15}H_{17}NNaO_5S^+$ $[M+Na]^+$ 346.0720, found 346.0718.

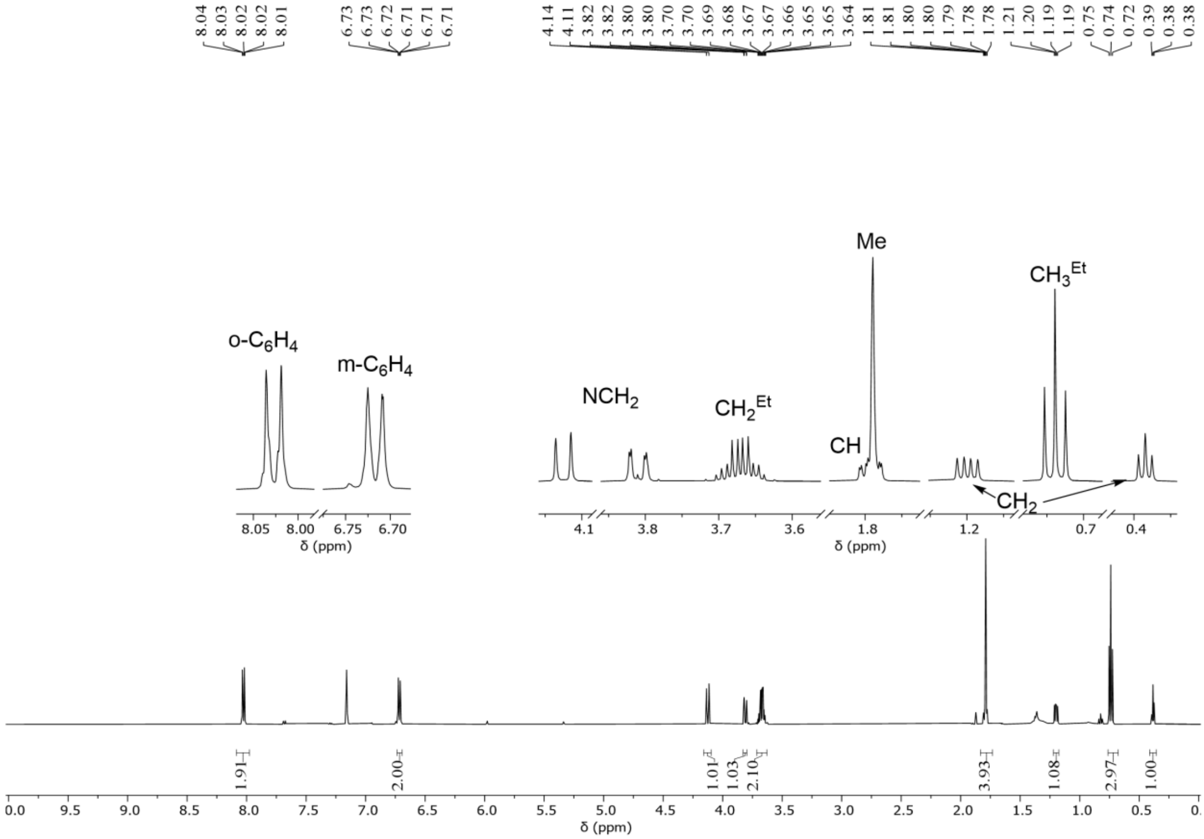

Figure S51. ^{1}H NMR (500 MHz, 298 K, C_6D_6) spectrum of compound (±)-**10a**.

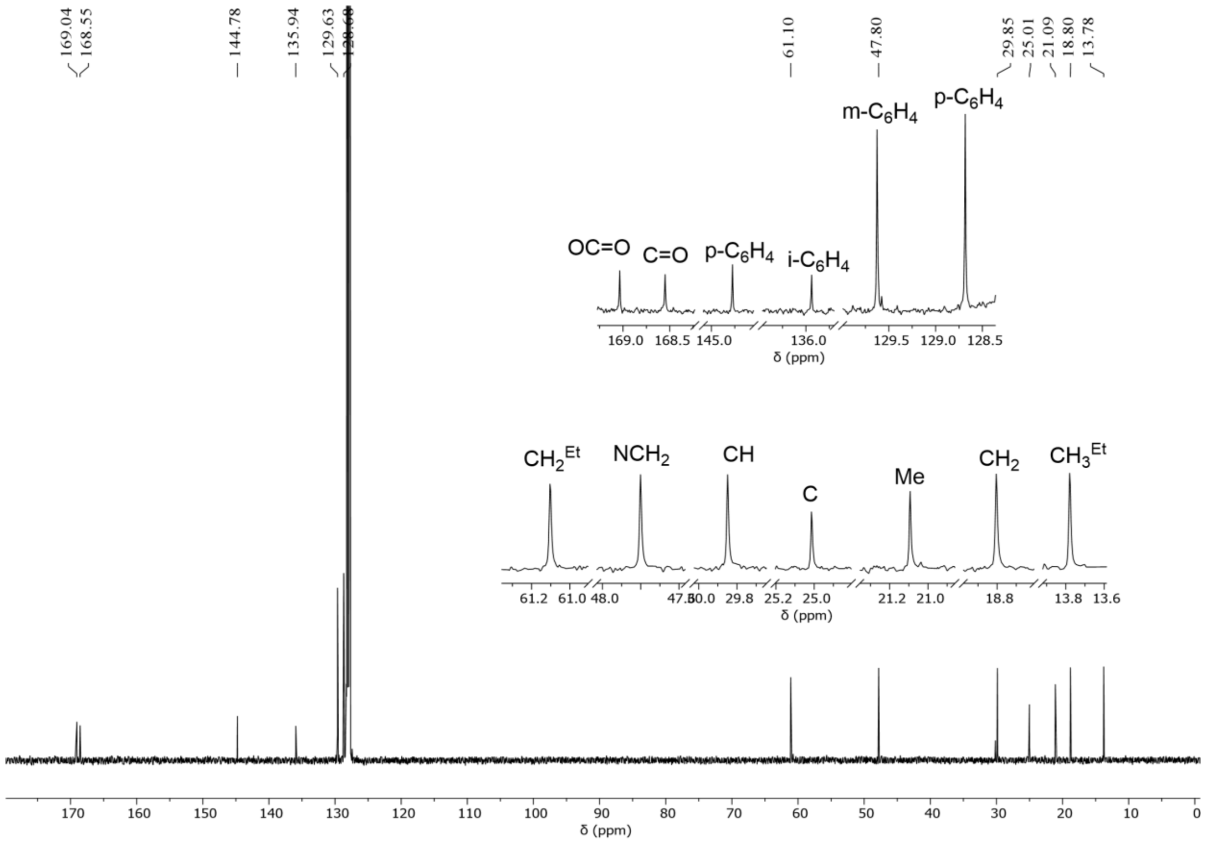

Figure S52. $^{13}C\{^1H\}$ NMR (125 MHz, 253 K, C_6D_6) spectrum of compound (±)-**10a**.

Synthesis of compound (±)-10b

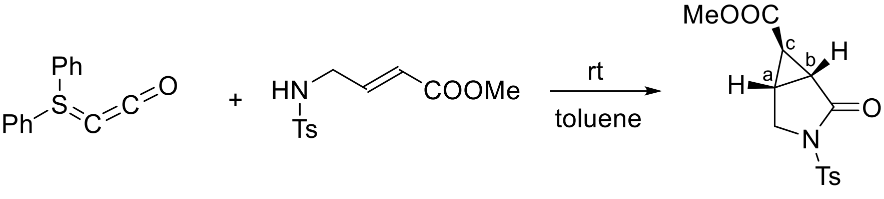

1 **8b** (±)-**10b**

A solution of compound **8b** (53.8 mg, 0.2 mmol) in toluene (1.0 mL) was added dropwise to a solution of reagent **1** (45.2 mg, 0.2 mmol) in toluene (0.5 mL) and stirred at room temperature for 15 min. All the volatiles were removed under reduced pressure, the remaining residue was purified by silica gel column chromatography (eluent: *n*-pentane/EtOAc = 2:1) to give compound (±)-**10b** (54.2 mg, 0.176 mmol, 88%) as a colorless solid. Crystals of compound (±)-**10b** suitable for X-ray diffraction were obtained from a solution of the colorless solid in EtOAc and *n*-pentane (ratio: 1:2) at rt.

Characterization data of compound (±)-**10b**:

m.p. 123 °C.
^{1}H NMR (500 MHz, 298 K, C_6D_6): δ = 7.98 (m, 2H, *o*-C_6H_4), 6.64 (m, 2H, *m*-C_6H_4), [3.43, 3.157](each m, each 1H, CH_2), 3.163 (s, 3H, OCH_3), 1.96 (m, 1H, CH^b), 1.83 (s, 3H, CH_3), 1.48 (m, 1H, CH^a), 1.40 (m, 1H, CH^c).
^{13}C{^{1}H} NMR (125 MHz, 298 K, C_6D_6): δ = 169.6 (OC=O), 169.1 (C=O), 144.9 (*p*-C_6H_4), 135.9 (*i*-C_6H_4), 129.6 (*m*-C_6H_4), 128.6 (*o*-C_6H_4), 51.7 (OCH_3), 47.9 (CH_2), 29.0 (CH^b), 24.9 (CH^c), 21.1 (Me), 20.5 (CH^a).
IR (ATR) [cm^{-1}]: ṽ = 1729, 1598, 1443, 1403, 1360, 1308, 1281, 1168, 1129, 1089, 1052, 1012, 985, 932, 891, 864, 815, 776, 755, 737, 705, 663, 633, 597, 548, 536, 503, 458, 422.
HR-MS-ESI(+) calc. $C_{14}H_{15}NNaO_5S^+$ $[M+Na]^+$ 332.0563, found 332.0566.

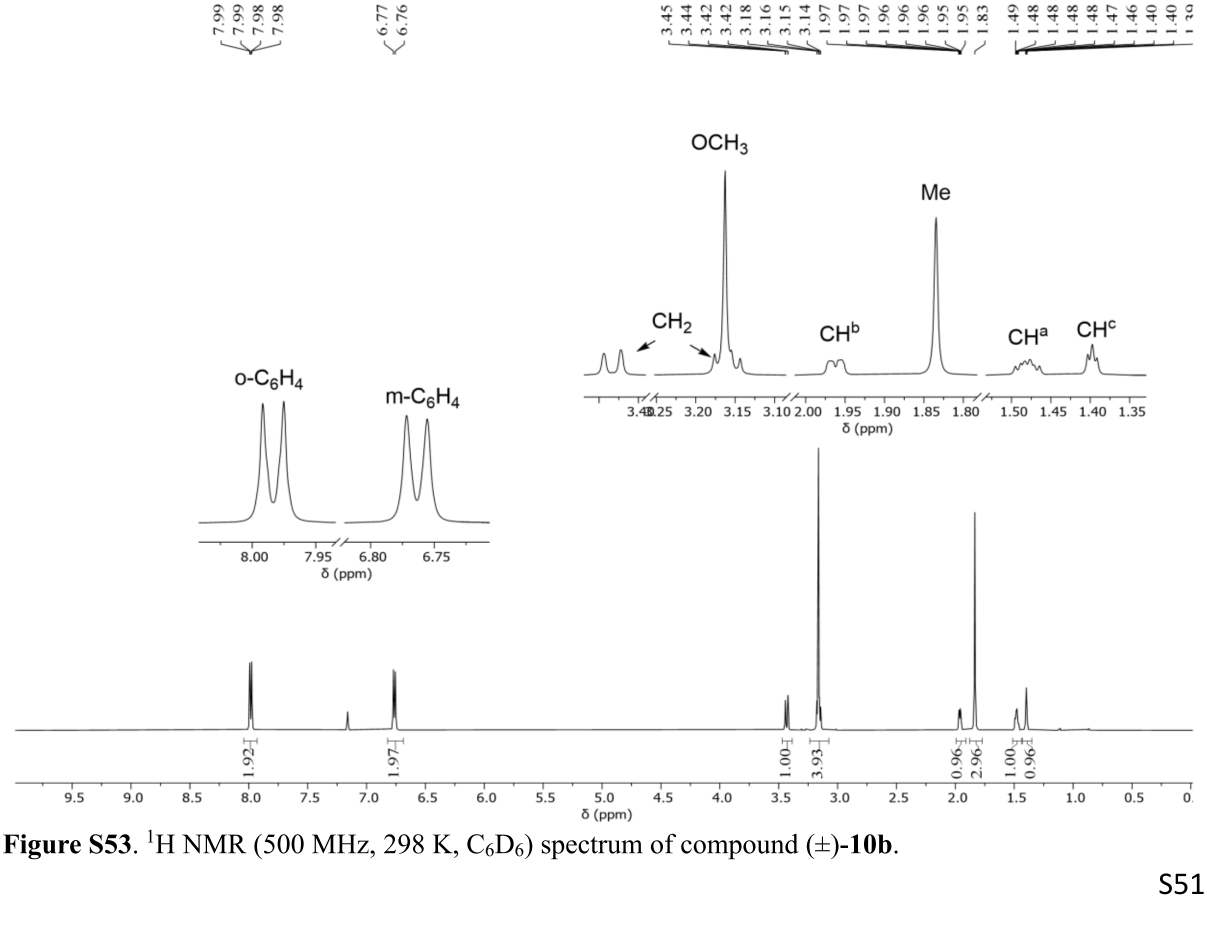

Figure S53. ^{1}H NMR (500 MHz, 298 K, C_6D_6) spectrum of compound (±)-**10b**.

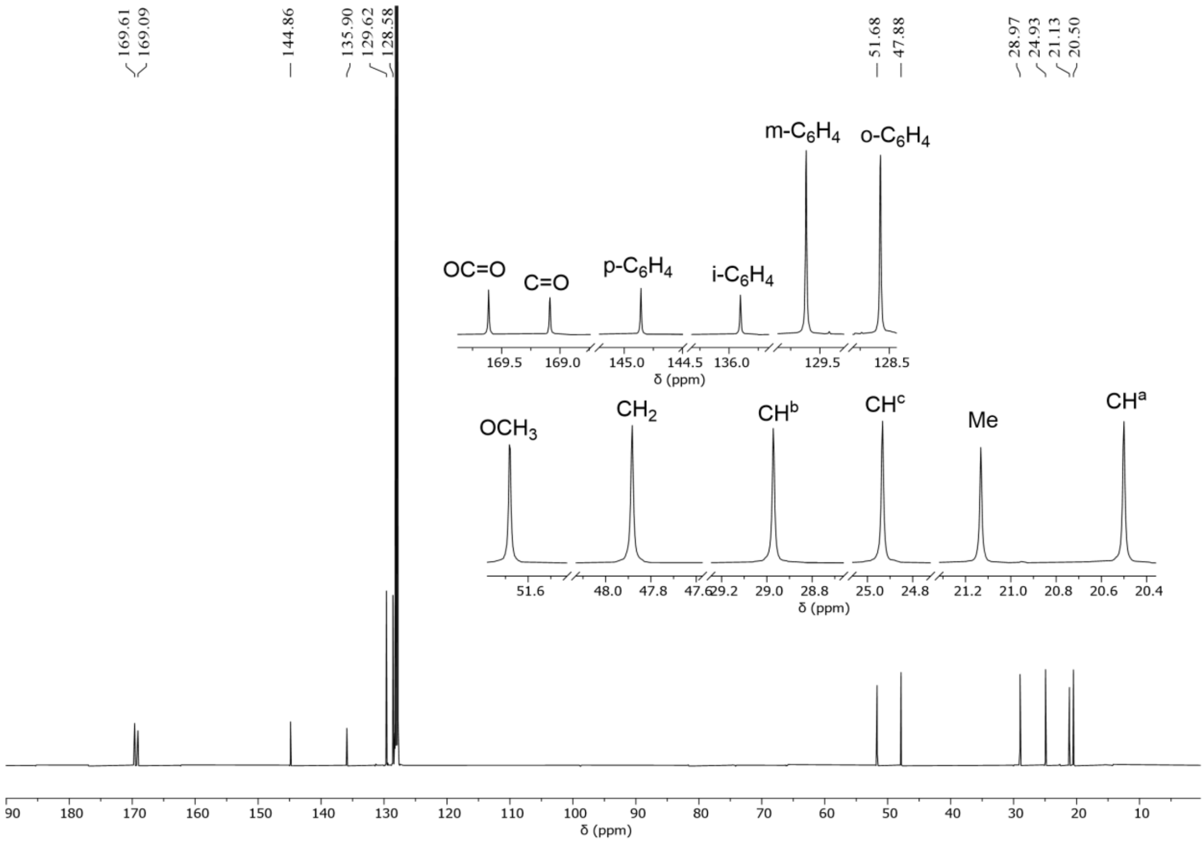

Figure S54. $^{13}C\{^{1}H\}$ NMR (125 MHz, 298 K, C_6D_6) spectrum of compound (±)-**10b**.

Synthesis of compound (±)-**10c**

*Experiment 1: the reaction of compound **1** and E-**8c***

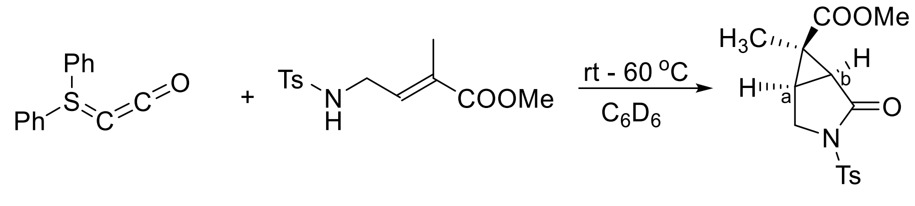

1 *E*-**8c** (±)-**10c**

A solution of compound *E*-**8c** (28.3 mg, 0.1 mmol) in C_6D_6 (0.5 mL) was added dropwise to a solution of reagent **1** (22.6 mg, 0.1 mmol) in C_6D_6 (0.5 mL) and kept at room temperature for 12 hours, and then at 60 °C for another 12 hours. All the volatiles were removed under reduced pressure, the remaining residue was purified by silica gel column chromatography (eluent: *n*-pentane/EtOAc = 2:1) to give compound (±)-**10c** (28.1 mg, 0.087 mmol, 87%) as a colorless solid.

Characterization data of compound (±)-**10c**:

m.p. 116 °C.

^{1}H NMR (500 MHz, 298 K, C_6D_6): δ = 8.03 (m, 2H, *o*-C_6H_4), 6.74 (m, 2H, *m*-C_6H_4), 3.40 (m, 2H, CH_2), 3.09 (s, 3H, OCH_3), 2.15 (m, 1H, CH^b), 1.81 (s, 3H, Me^{C6H4}), 1.51 (m, 1H, CH^a), 0.96 (s, 3H, Me).

^{13}C{^{1}H} NMR (125 MHz, 298 K, C_6D_6): δ = 171.4 (OC=O), 168.3 (C=O), 144.9 (*p*-C_6H_4), 136.2 (*i*-C_6H_4), 129.6 (*m*-C_6H_4), 128.7 (*o*-C_6H_4), 51.9 (OCH_3), 45.1 (CH_2), 34.1 (CH^b), 27.4 (C), 25.1 (CH^a), 21.1 (Me^{C6H4}), 7.6 (Me).

IR (ATR) [cm^{-1}]: $\tilde{v}$ = 1727, 1598, 1438, 1362, 1299, 1242, 1189, 1170, 1140, 1091, 1042, 1014, 870, 816, 757, 706, 668, 646, 601, 550, 538, 504, 465.

HR-MS-ESI(+) calc. $C_{15}H_{17}NNaO_5S^+$ $[M+Na]^+$ 346.0720, found 346.0724.

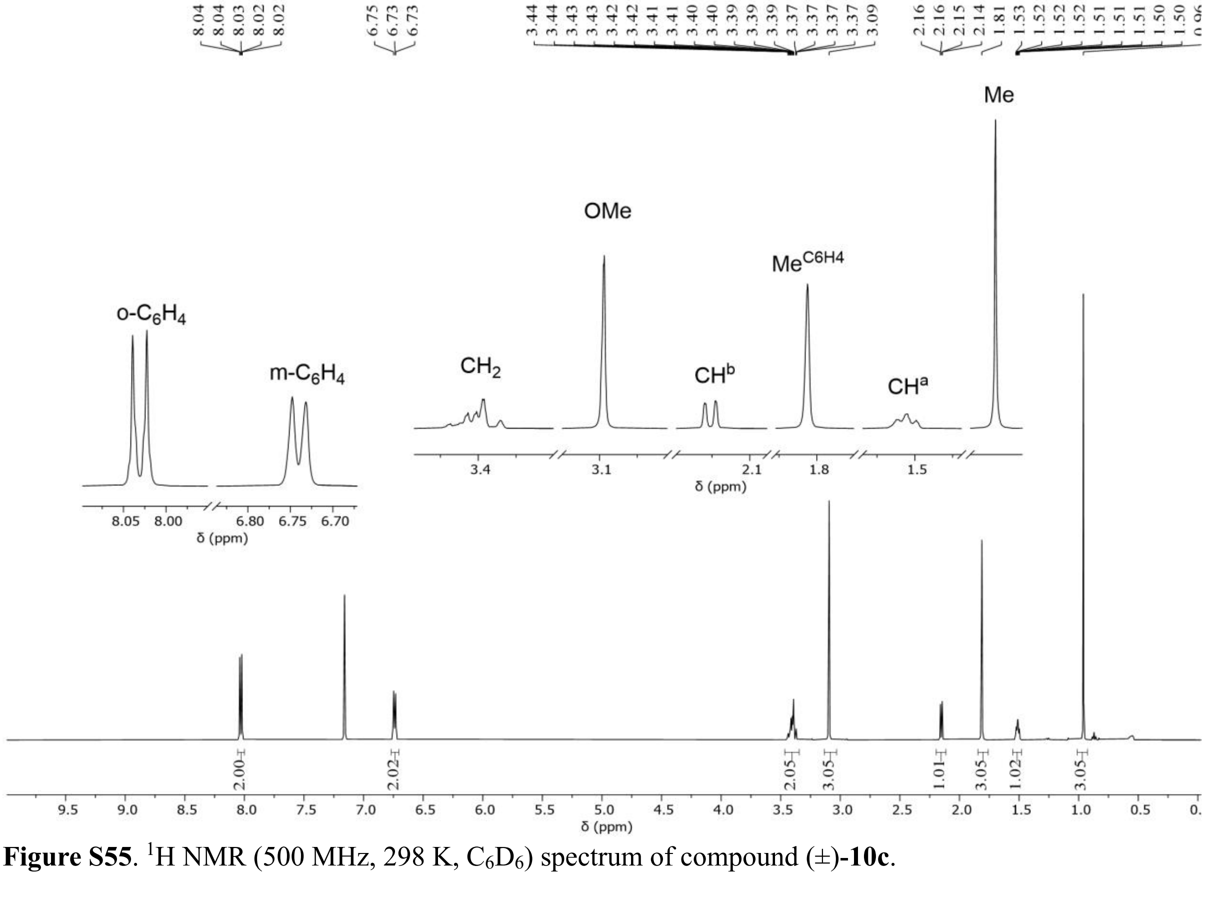

Figure S55. ^{1}H NMR (500 MHz, 298 K, C_6D_6) spectrum of compound (±)-**10c**.

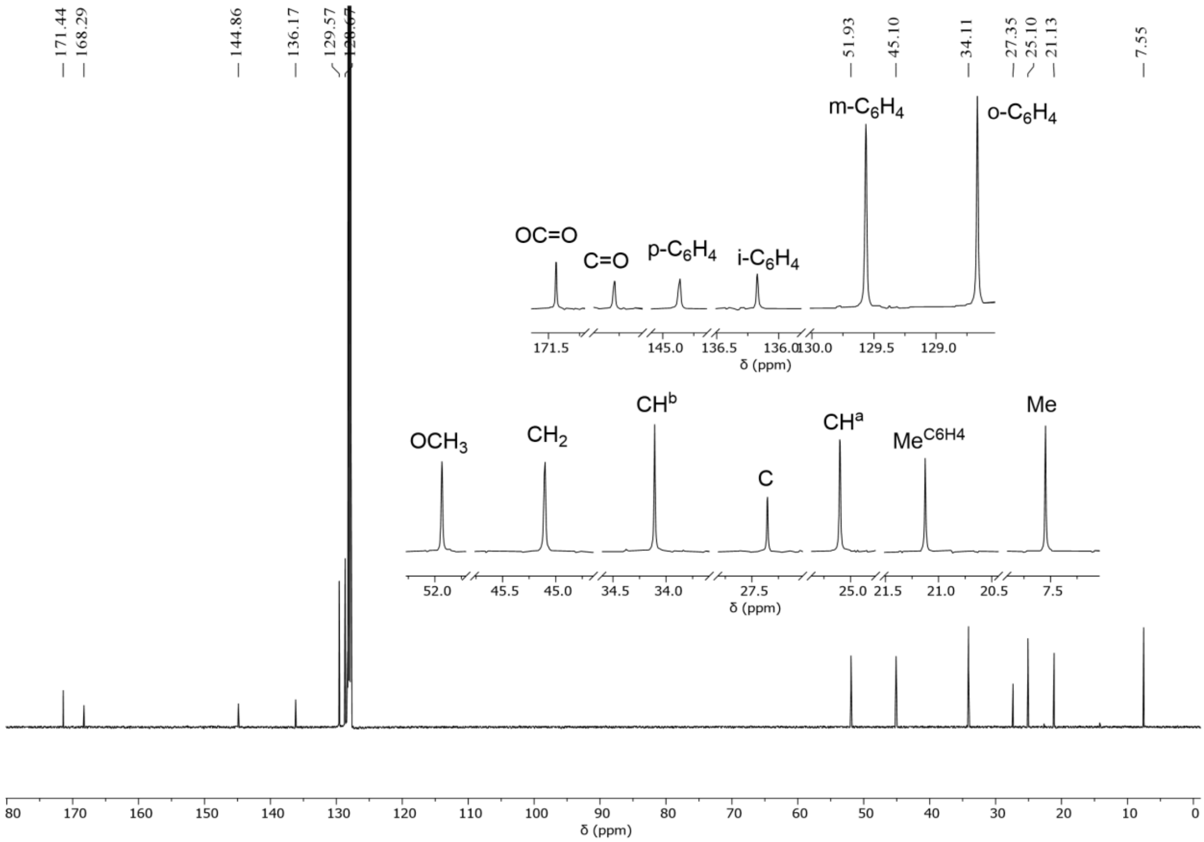

Figure S56. $^{13}C\{^1H\}$ NMR (125 MHz, 298 K, C_6D_6) spectrum of compound (±)-**10c**.

*Experiment 2: the reaction of compound **1** and Z-**8c***

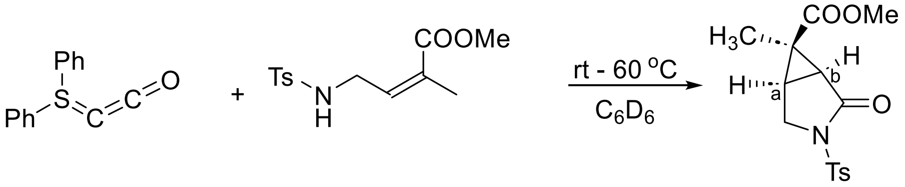

1 *Z*-**8c** (±)-**10c**

A solution of compound *Z*-**8c** (28.3 mg, 0.1 mmol) in C_6D_6 (0.5 mL) was added dropwise to a solution of compound **1** (22.6 mg, 0.1 mmol) in C_6D_6 (0.5 mL) and kept at room temperature for 12 hours, and then at 60 °C for another 12 hours. All the volatiles were removed under reduced pressure, the remaining residue was purified by silica gel column chromatography (eluent: *n*-pentane/EtOAc = 2:1) to give compound (±)-**10c** (27.8 mg, 0.086 mmol, 86%) as a colorless solid.

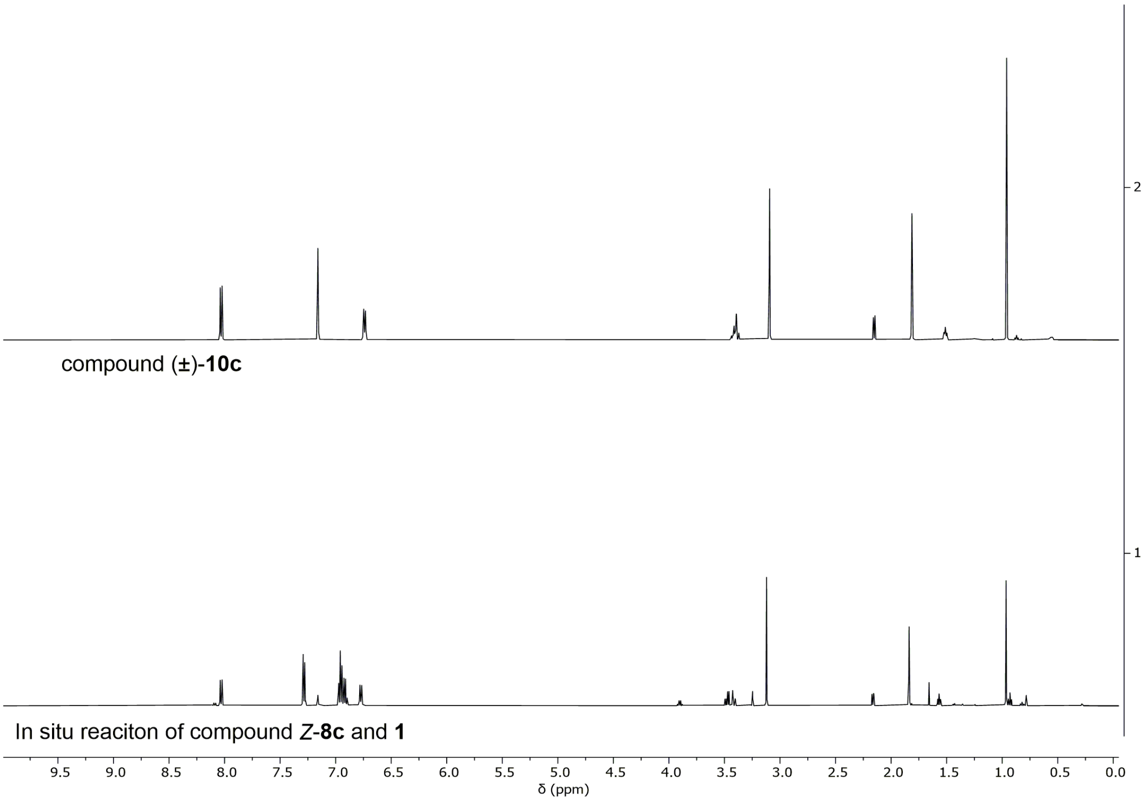

Figure S57. ^{1}H NMR (500 MHz, 298 K, C_6D_6) spectra of (1) in situ reaction of compound **1** and Z-**8c** and (2) compound (±)-**10c**.

Synthesis of compound (±)-10d

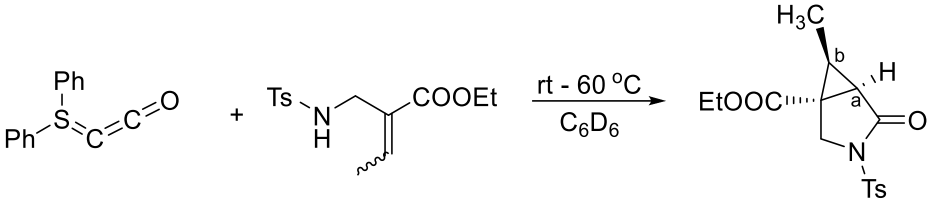

1 **8d** (±)-**10d**

A solution of compound **8d** (29.7 mg, 0.1 mmol, *Z*-**8d** : *E*-**8d** = 1.8:1) in C_6D_6 (0.5 mL) was added dropwise to a solution of reagent **1** (22.6 mg, 0.1 mmol) in C_6D_6 (0.5 mL) and kept at room temperature for 12 hours, and then at 60 °C for another 12 hours. All the volatiles were removed under reduced pressure, the remaining residue was purified by silica gel column chromatography (eluent: *n*-pentane/EtOAc = 2:1) to give compound (±)-**10d** (25.6 mg, 0.076 mmol, 76%) as a colorless solid.

Characterization data of compound (±)-**10d**:

m.p. 119 °C.
^{1}H NMR (500 MHz, 298 K, C_6D_6): δ = 8.04 (m, 2H, *o*-C_6H_4), 6.70 (m, 2H, *m*-C_6H_4), [4.42, 3.81](each m, each 1H, CH_2), 3.73 (CH_2^{Et}), 2.03 (m, 1H, CH^a), 1.78 (s, 3H, Me^{C6H4}), 1.62 (m, 1H, CH^b), 0.79 (t, $^3J_{HH}$ = 7.1 Hz, CH_3^{Et}), 0.57 (d, $^3J_{HH}$ = 6.6 Hz, 3H, Me).
^{13}C{^{1}H} NMR (125 MHz, 298 K, C_6D_6): δ = 169.2 (OC=O), 167.4 (C=O), 144.8 (*p*-C_6H_4), 136.2 (*i*-C_6H_4), 129.6 (*m*-C_6H_4), 128.7 (*o*-C_6H_4), 61.2 (OCH_2), 44.5 (CH_2), 34.9 (CH^a), 29.3 (C), 24.4 (CH^b), 21.1 (Me^{C6H4}), 13.8 (CH_3^{Et}), 6.4 (Me).
IR (ATR) [cm^{-1}]: ṽ = 2986, 1730, 1599, 1480, 1417, 1369, 1330, 1294, 1249, 1213, 1189, 1172, 1138, 1092, 1017, 985, 910, 817, 755, 724, 706, 673, 654, 606, 550.
HR-MS-ESI(+) calc. $C_{16}H_{19}NNaO_5S^+$ $[M+Na]^+$ 360.0876, found 360.0877.

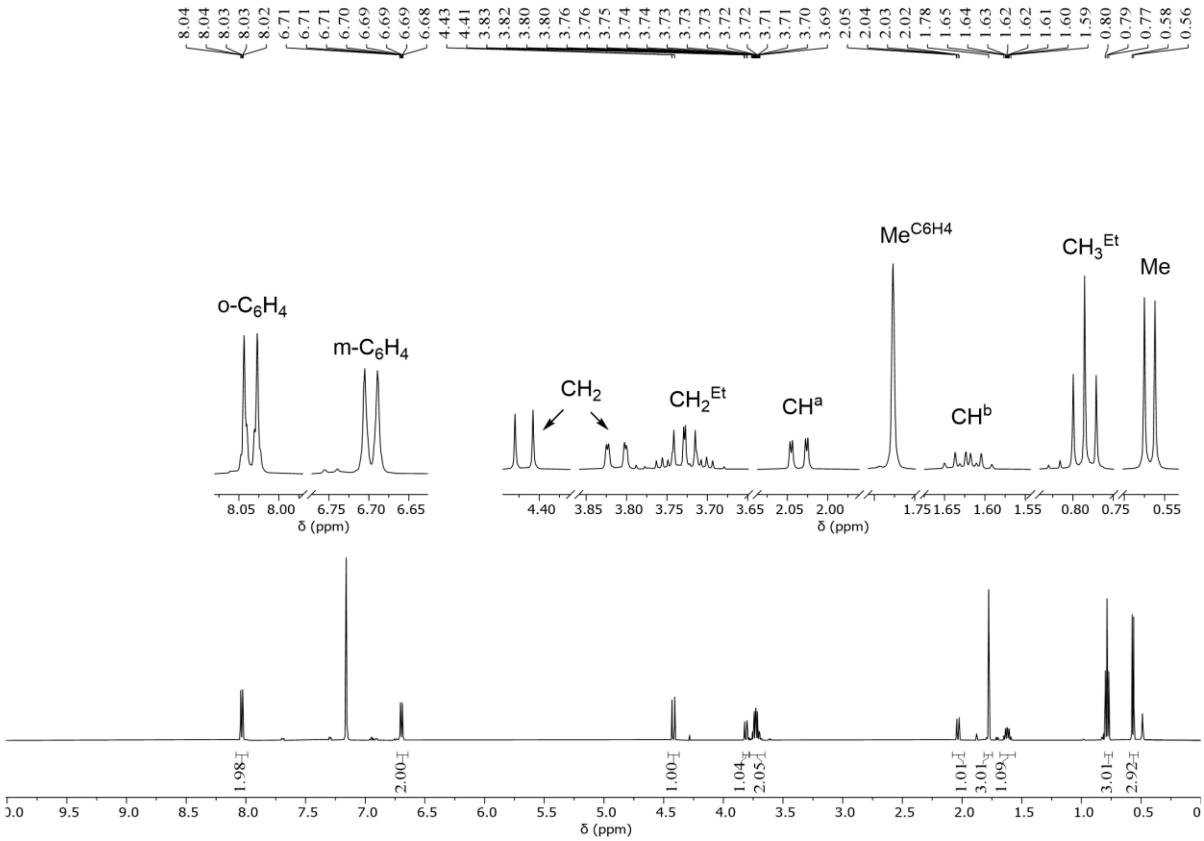

Figure S58. ^{1}H NMR (500 MHz, 298 K, C_6D_6) spectrum of compound (±)-**10d**.

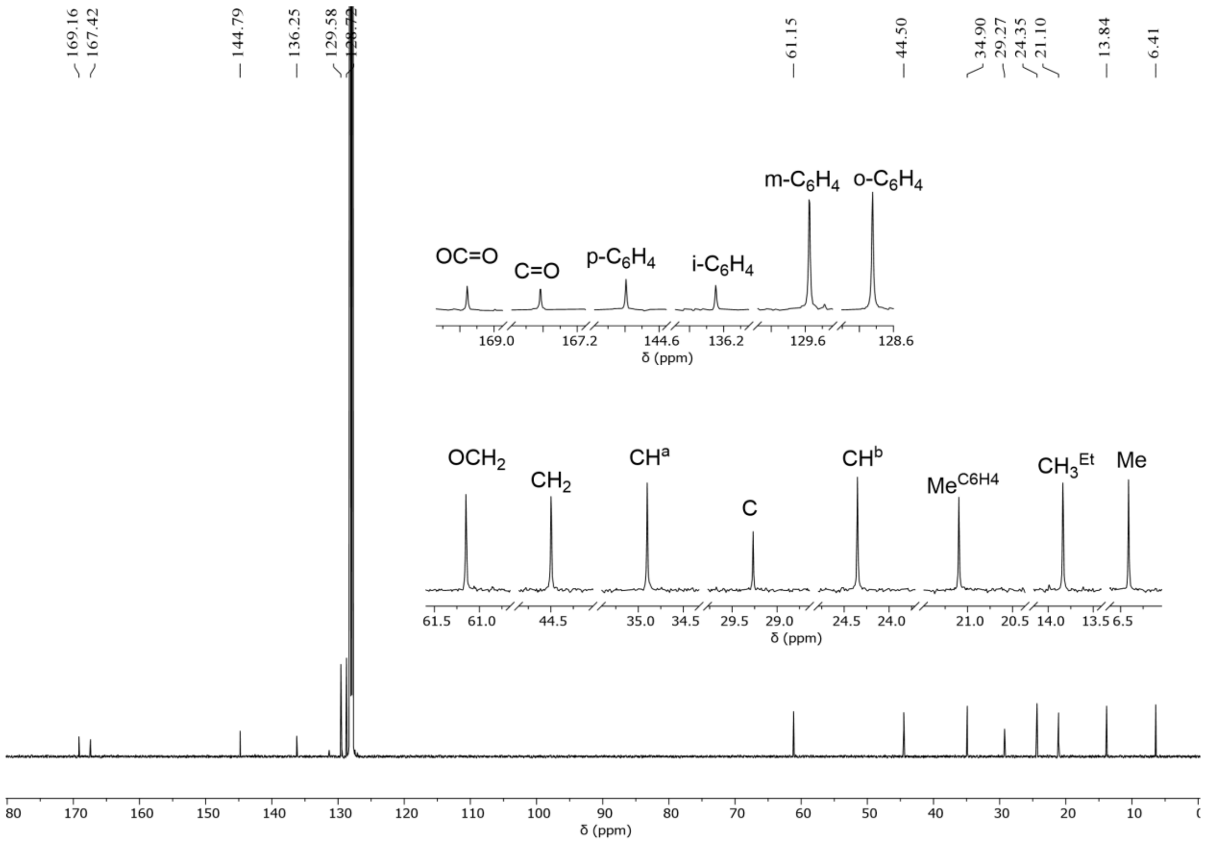

Figure S59. $^{13}C\{^{1}H\}$ NMR (125 MHz, 298 K, C_6D_6) spectrum of compound (±)-**10d**.

Synthesis of compound (±)-10e

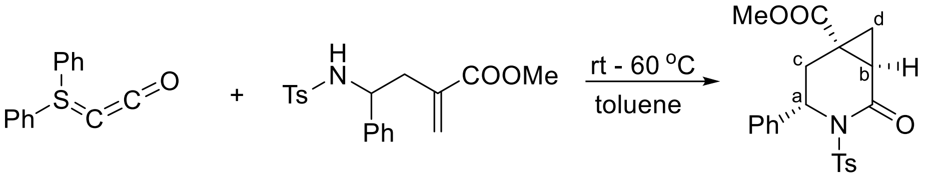

1 **8e** (±)-**10e**

A solution of compound **8e** (71.8 mg, 0.2 mmol) in toluene (1.0 mL) was added dropwise to a solution of reagent **1** (45.2 mg, 0.2 mmol) in toluene (0.5 mL) and kept at room temperature for 12 hours, and then at 60 °C for another 12 hours. All the volatiles were removed under reduced pressure, the remaining residue was purified by silica gel column chromatography (eluent: *n*-pentane/EtOAc = 1:1) to give compound (±)-**10e** (68.6 mg, 0.172 mmol, 86%, d.r. = 1.2:1) as a colorless solid. The *in situ* ^{1}H-NMR spectrum shows a d.r. of 6:5.

Characterization data of compound (±)-**10e**:

m.p. 130 °C.
^{1}H NMR (500 MHz, 298 K, C_6D_6) for major isomer: δ = 7.88 (m, 2H, *o*-C_6H_4), 6.94 (m, 5H, Ph), 6.63 (m, 2H, *m*-C_6H_4), 5.77 (m, 1H, CH^a), [3.01, 1.61](each m, each 1H, CH_2^c), 2.81 (s, 3H, OMe), 2.35 (m, 1H, CH^b), 1.79 (s, 3H, Me^{C6H4}), [1.30, 0.67](each m, each 1H, CH_2^d).
^{1}H NMR (500 MHz, 298 K, C_6D_6) for minor isomer: δ = 8.09 (m, 2H, *o*-C_6H_4), [7.15, 7.03, 6.95](each m, 5H, Ph), 6.76 (m, 2H, *m*-C_6H_4), 5.94 (m, 1H, CH^a), 3.05 (s, 3H, OMe), [2.64, 2.43](each m, each 1H, CH_2^c), 2.23 (m, 1H, CH^b), 1.83 (s, 3H, Me^{C6H4}), [1.78, 0.85](each m, each 1H, CH_2^d).
^{13}C{^{1}H} NMR (125 MHz, 298 K, C_6D_6) for major isomer [Phenyl group was not listed]: δ = 171.1 (OC=O), 168.1 (C=O), 144.3 (*p*-C_6H_4), 136.7 (*i*-C_6H_4), 130.3 (*o*-C_6H_4), 128.9 (*m*-C_6H_4), 60.8 (CH^a), 51.5 (OMe), 34.6 (CH_2^c), 27.61 (CH^b), 25.6 (CH_2^d), 24.5 (C), 21.10 (Me^{C6H4}).
^{13}C{^{1}H} NMR (125 MHz, 298 K, C_6D_6) for minor isomer [Phenyl group was not listed]: δ = 171.0 (OC=O), 168.5 (C=O), 144.5 (*p*-C_6H_4), 137.3 (*i*-C_6H_4), 130.3 (*o*-C_6H_4), 129.1 (*m*-C_6H_4), 57.0 (CH^a), 51.6 (OMe), 28.5 (CH_2^c), 27.62 (CH^b), 27.0 (C), 21.13 (Me^{C6H4}) 17.9 (CH_2^d).
IR (ATR) [cm^{-1}]: ṽ = 2957, 1731, 1695, 1599, 1498, 1450, 1394, 1354, 1309, 1273, 1233, 1189, 1170, 1150, 1121, 1089, 974, 813, 757, 724, 703, 678, 645, 604, 589, 543, 504.
HR-MS-ESI(+) calc. $C_{21}H_{21}NNaO_5S^+$ $[M+Na]^+$ 422.1033, found 422.1030.

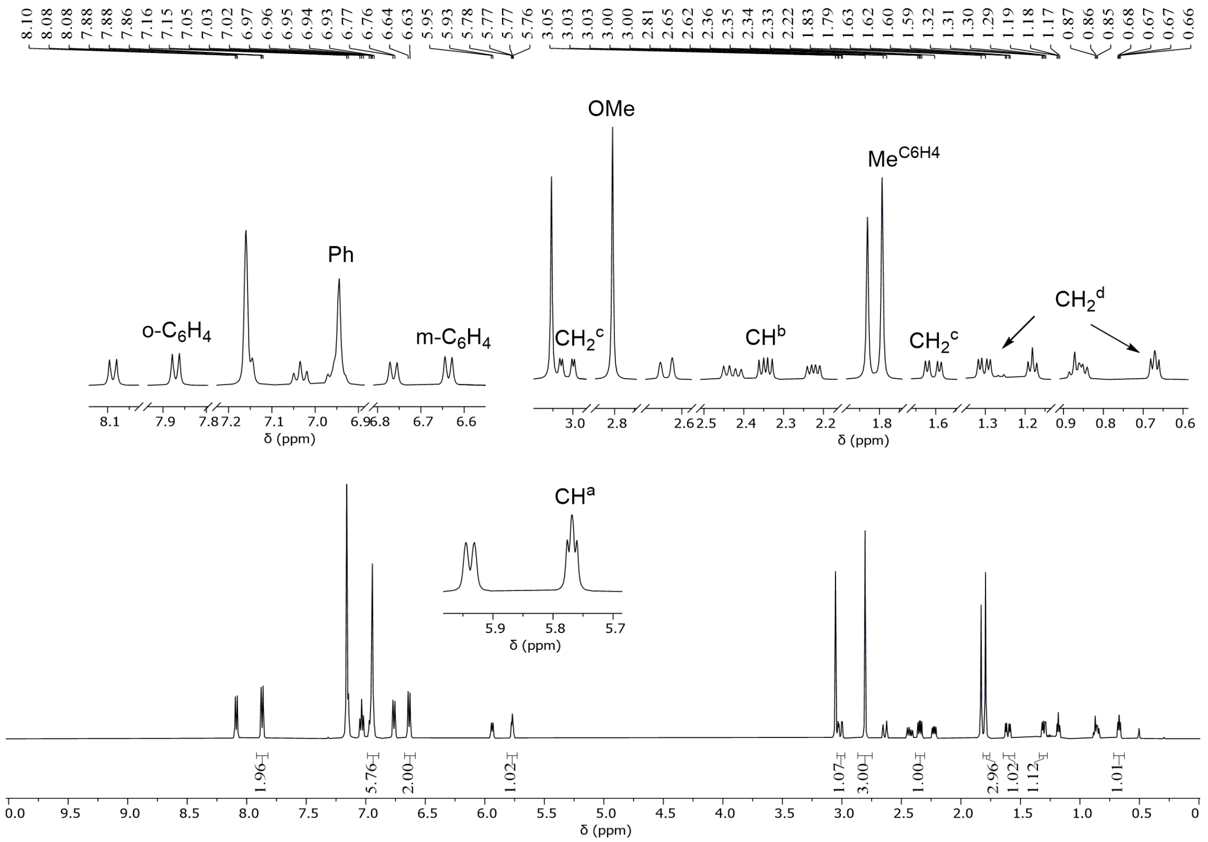

Figure S60. ^{1}H NMR (500 MHz, 298 K, C_6D_6) spectrum of compound (±)-**10e** (the marked peaks from the major isomer).

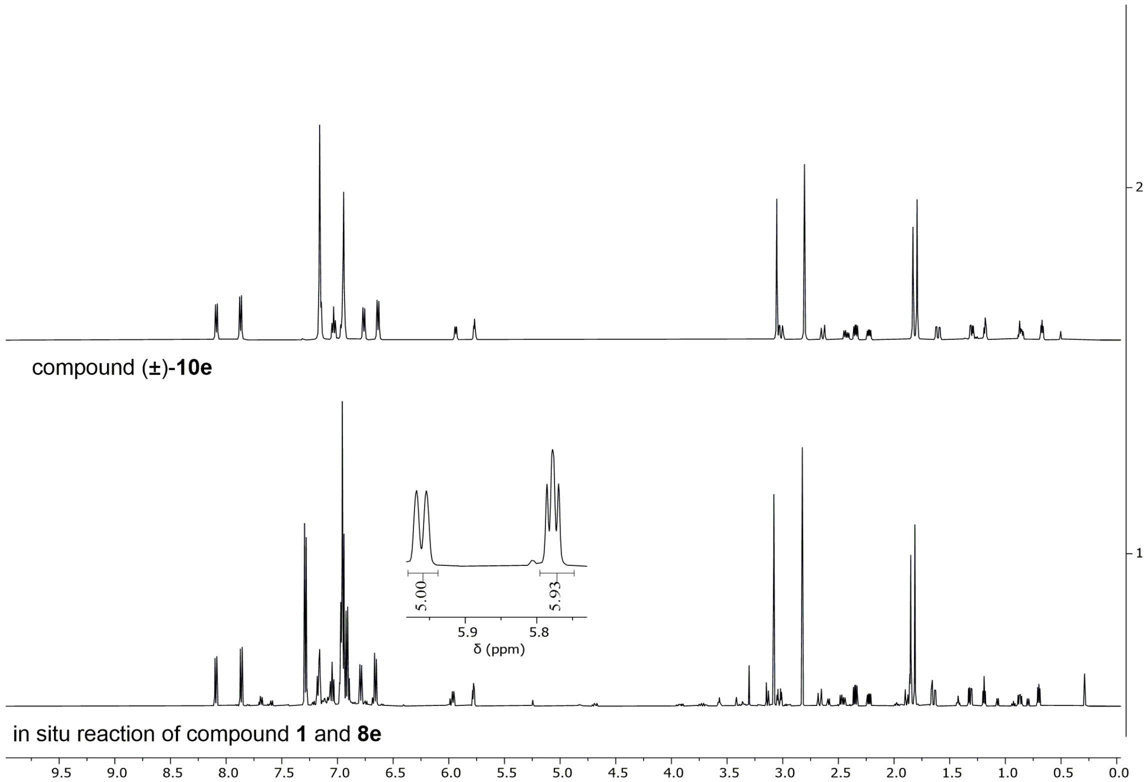

Figure S61. ^{1}H NMR (500 MHz, 298 K, C_6D_6) spectra of (1) *in-situ* reaction of compound **1** and **8e**, (2) compound (±)-**10e**.

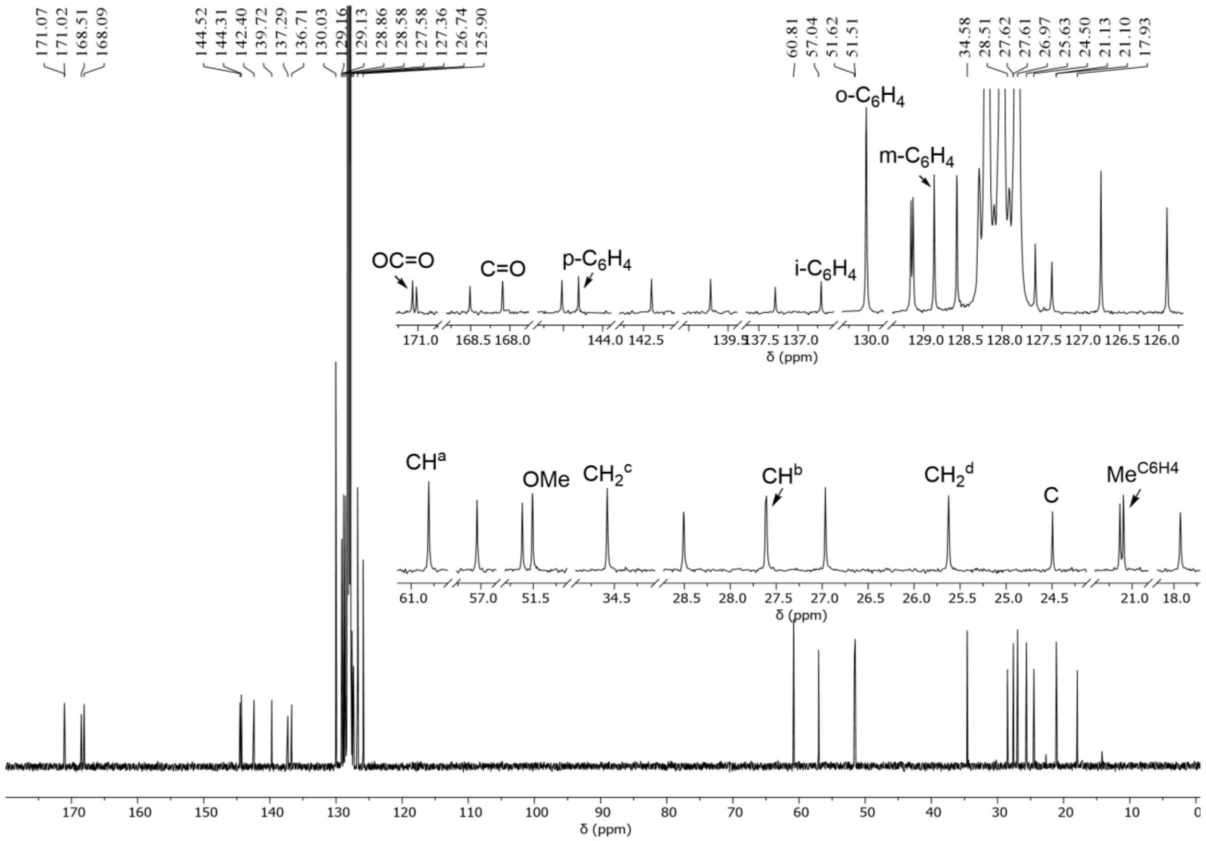

Figure S62. $^{13}C\{^{1}H\}$ NMR (125 MHz, 298 K, C_6D_6) spectrum of compound (±)-**10e**.

Synthesis of compound (±)-10f

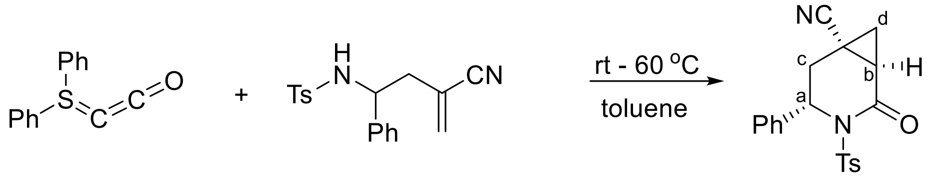

1 **8f** (±)-**10f**

A solution of compound **8f** (65.2 mg, 0.2 mmol) in toluene (1.0 mL) was added dropwise to a solution of reagent **1** (45.2 mg, 0.2 mmol) in toluene (0.5 mL) and kept at room temperature for 12 hours, and then at 60 °C for another 12 hours. All the volatiles were removed under reduced pressure, the remaining residue was purified by silica gel column chromatography (eluent: *n*-pentane/EtOAc = 1:1) to give compounds (±)-**10f** (58.6 mg, 0.160 mmol, 80%) as a colorless solid. Crystals of compound (±)-**10f** suitable for X-ray diffraction were obtained from a solution of the colorless solid in EtOAc and *n*-pentane (ratio: 1:1) at rt. The *in situ* ^{1}H-NMR spectrum shows an d.r. of 5.2:1.

Characterization data of compound (±)-**10f**:

^{1}H NMR (500 MHz, 298 K, C_6D_6) for major isomer: δ = 7.79 (m, 2H, *o*-C_6H_4), [7.03 (3H), 6.89 (2H)](each m, Ph), 6.62 (m, 2H, *m*-C_6H_4), 5.59 (m, each 1H, CH^a), [2.21, 1.32](each m, each 1H, CH_2^c), 1.77 (s, 3H, Me^{C6H4}), 1.65 (m, 1H, CH^b), [0.74, 0.33](each m, each 1H, CH_2^d).
^{13}C{^{1}H} NMR (125 MHz, 298 K, C_6D_6) for major isomer: δ = 166.1 (C=O), 144.8 (*p*-C_6H_4), [138.5, 129.0, 128.4, 126.5](Ph), 136.2 (*i*-C_6H_4), 130.0 (*o*-C_6H_4), 129.2 (*m*-C_6H_4), 119.8 (CN), 60.2 (CH^a), 37.0 (CH_2^c), 24.7 (CH^b), 24.1 (CH_2^d), 21.2 (Me^{C6H4}), 10.3 (C).
IR (ATR) [cm^{-1}]: $\tilde{v}$ = 2242, 1700, 1598, 1498, 1454, 1391, 1357, 1290, 1208, 1189, 1171, 1123, 1089, 1002, 975, 919, 804, 750, 724, 703, 687, 666, 613, 581, 552, 538, 504.
HR-MS-ESI(+) calc. $C_{20}H_{19}N_2NaO_3S^+$ $[M+Na]^+$ 389.0930, found 389.0933.

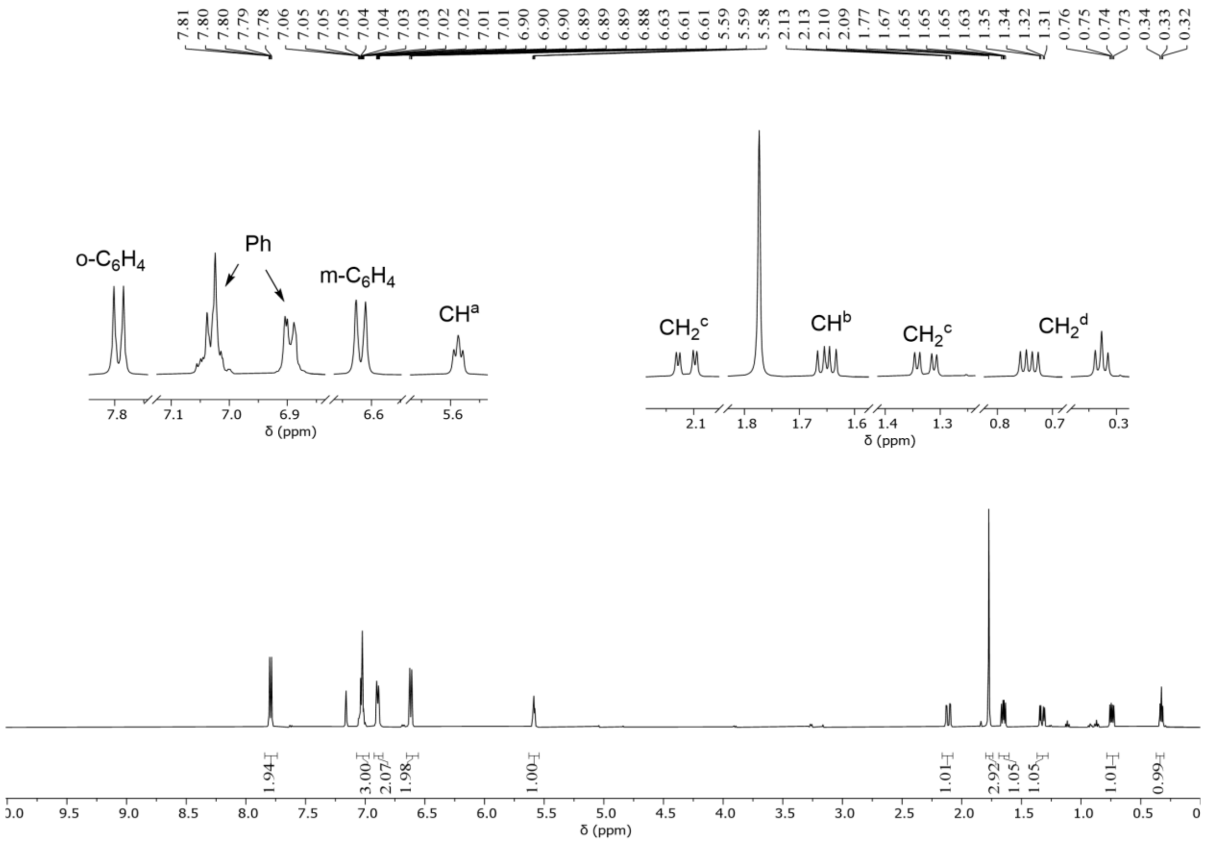

Figure S63. ^{1}H NMR (500 MHz, 298 K, C_6D_6) spectrum of the major isomer of compound (±)-**10f**.

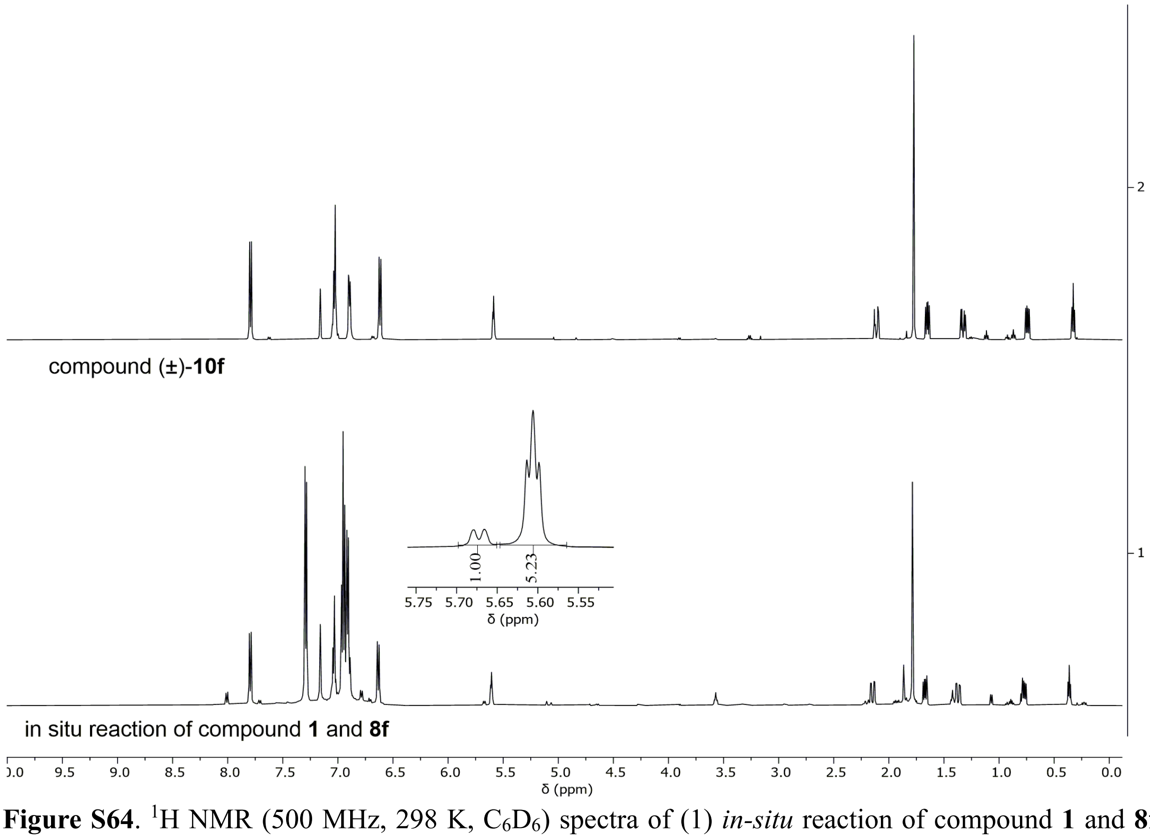

Figure S64. ^{1}H NMR (500 MHz, 298 K, C_6D_6) spectra of (1) *in-situ* reaction of compound **1** and **8f**, (2) compound (±)-**10f**.

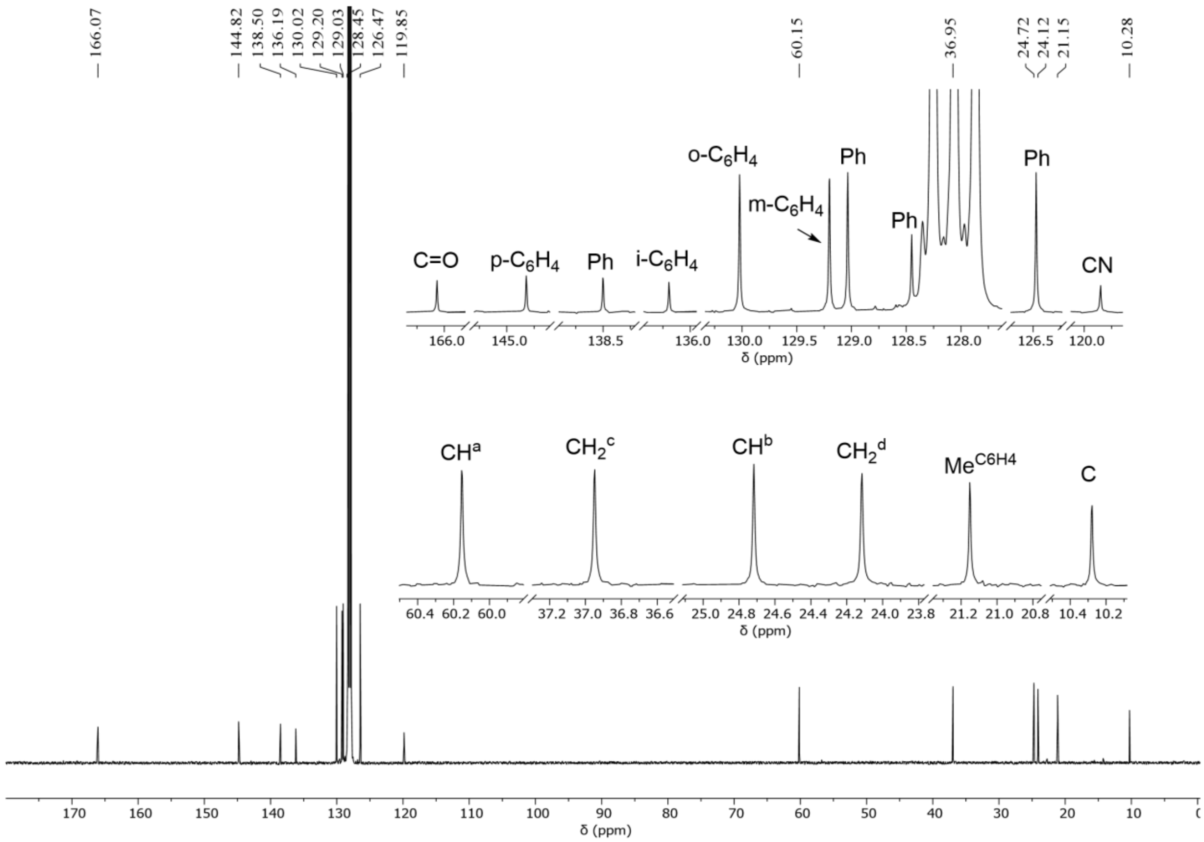

Figure S65. $^{13}C\{^{1}H\}$ NMR (125 MHz, 298 K, C_6D_6) spectrum of compound (±)-**10f**.

Synthesis of compound 10g

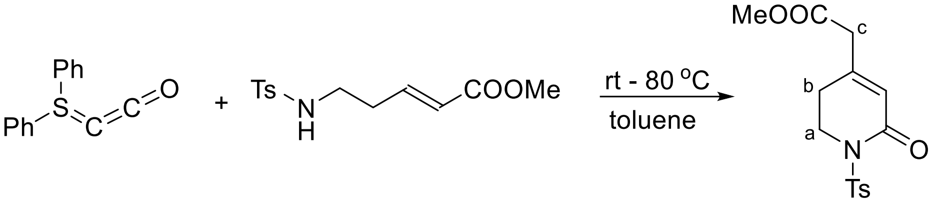

1 **8g** **10g**

A solution of compound **8g** (56.6 mg, 0.2 mmol) in toluene (1.0 mL) was added dropwise to a solution of compound **1** (45.2 mg, 0.2 mmol) in toluene (0.5 mL) and kept at room temperature for 12 hours, and then at 80 °C for another 12 hours. All the volatiles were removed under reduced pressure, the remaining residue was purified by silica gel column chromatography (eluent: *n*-pentane/EtOAc = 2:1) to give compound **10g** (44.6 mg, 0.138 mmol, 69%) as a colorless solid.

Characterization data of compound **10g**:

m.p. 86 °C.

^{1}H NMR (500 MHz, 298 K, C_6D_6): δ = 8.09 (m, 2H, *o*-C_6H_4), 6.76 (m, 2H, *m*-C_6H_4), 5.40 (m, 1H, =CH), 3.64 (t, $^3J_{HH}$ = 6.5 Hz, 2H, CH_2^a), 3.17 (s, 3H, OMe), 2.38 (s, 2H, CH_2^c), 1.84 (s, 3H, Me^{C6H4}), 1.73 (m, 2H, CH_2^b).

^{13}C{^{1}H} NMR (125 MHz, 298 K, C_6D_6): δ = 168.8 (OC=O), 162.4 (C=O), 151.2 (=C), 144.1 (*p*-C_6H_4), 137.2 (*i*-C_6H_4), 129.3 (*m*-C_6H_4), 129.1 (*o*-C_6H_4), 123.3 (=CH), 51.5 (OMe), 43.8 (CH_2^a), 40.7 (CH_2^c), 28.4 (CH_2^b), 21.1 (Me^{C6H4}).

IR (ATR) [cm^{-1}]: ṽ = 2959, 1733, 1682, 1646, 1597, 1495, 1462, 1436, 1391, 1342, 1288, 1214, 1188, 1164, 1125, 1088, 1036, 1020, 1004, 972, 945, 914, 874, 816, 736, 707, 683, 648, 587, 570, 535, 511, 478, 444.

HR-MS-ESI(+) calc. $C_{15}H_{17}NNaO_5S^+$ [M+Na]$^+$ 346.0720, found 346.0718.

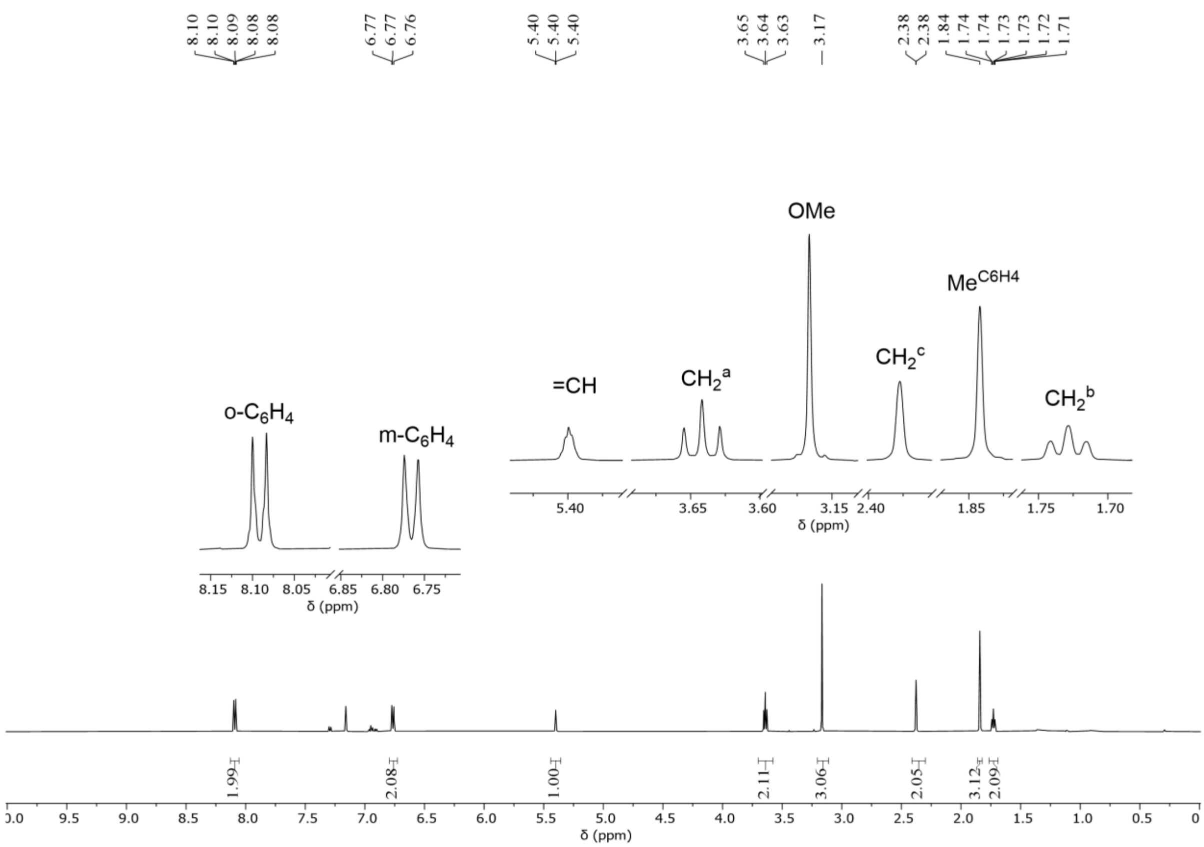

Figure S66. ^{1}H NMR (500 MHz, 298 K, C_6D_6) spectrum of compound **10g**.

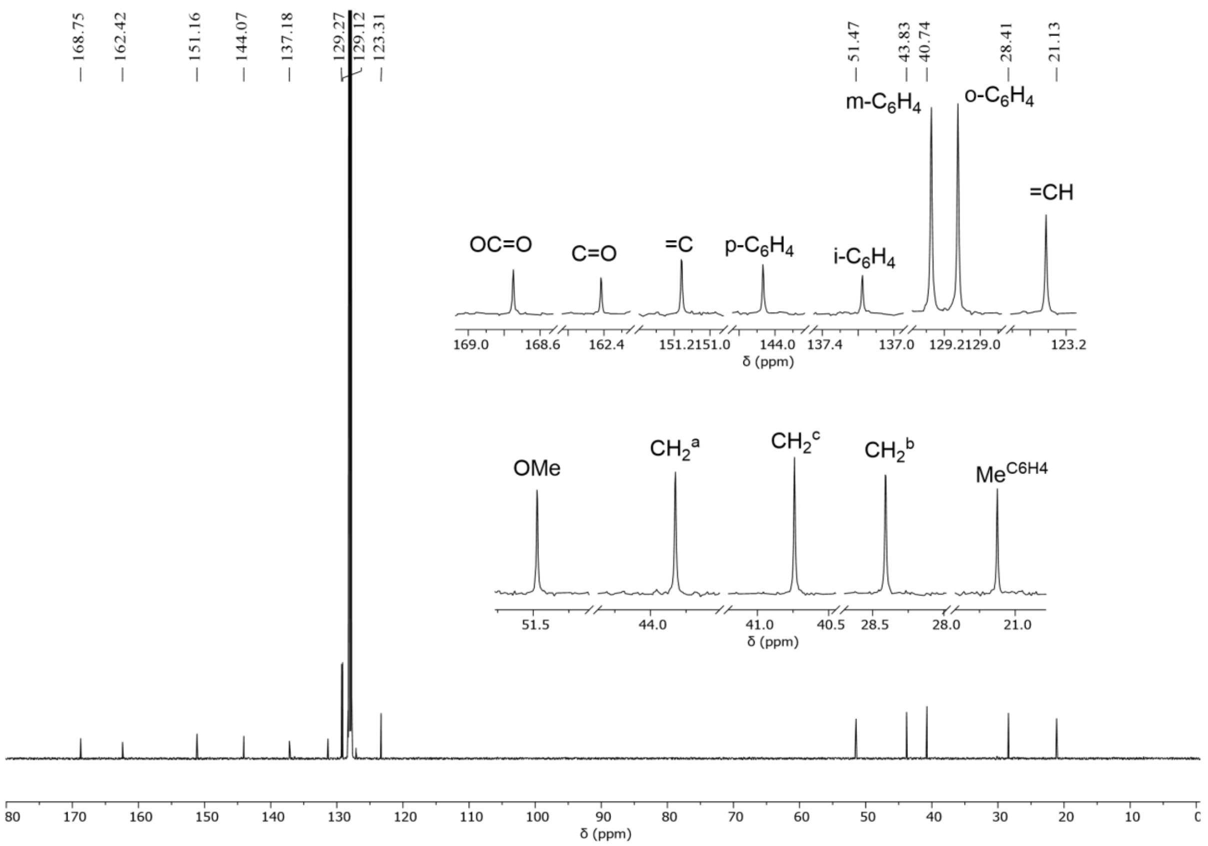

Figure S67. $^{13}C\{^1H\}$ NMR (125 MHz, 298 K, C_6D_6) spectrum of compound **10g**.

Synthesis of compound (±)-10h

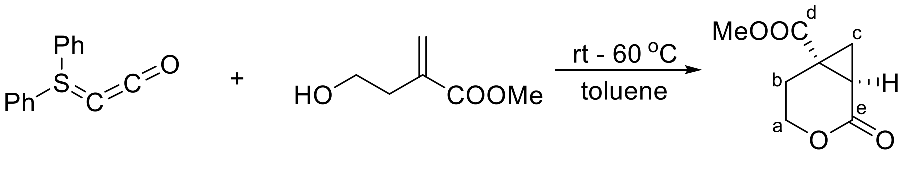

A solution of compound **8h** (26.0 mg, 0.2 mmol) in toluene (1.0 mL) was added dropwise to a solution of compound **1** (45.2 mg, 0.2 mmol) in toluene (0.5 mL) and kept at room temperature for 12 hours, and then at 60 °C for another 12 hours. All the volatiles were removed under reduced pressure, the remaining residue was purified by silica gel column chromatography (eluent: *n*-pentane/EtOAc = 4:1) to give compound (±)-**10h** (22.8 mg, 0.134 mmol, 67%) as a colorless oil.

Characterization data of compound (±)-**10h**:

^{1}H NMR (500 MHz, 298 K, C_6D_6): δ = 3.47 (m, 2H, CH_2^a), 3.25 (s, 3H, OMe), 3.28 (m, 1H, CH), [1.77, 1.62](each m, each 1H, CH_2^b), [1.26, 1.05](each m, each 1H, CH_2^c).
^{13}C{^{1}H} NMR (125 MHz, 298 K, C_6D_6): δ = 176.3 (C=O^e), 170.6 (C=O^d), 65.4 (CH_2^a), 51.4 (OMe), 28.0 (C), 26.0 (CH_2^b), 25.9 (CH), 19.8 (CH_2^c).
IR (ATR) [cm^{-1}]: ṽ = 2926, 2856, 1773, 1727, 1636, 1442, 1405, 1382, 1324, 1264, 1225, 1179, 1159, 1126, 1026, 990, 819, 749, 706, 418.
HR-MS-ESI(+) calc. $C_8H_{10}NaO_4^+$ [M+Na]$^+$ 193.0471, found 193.0471.

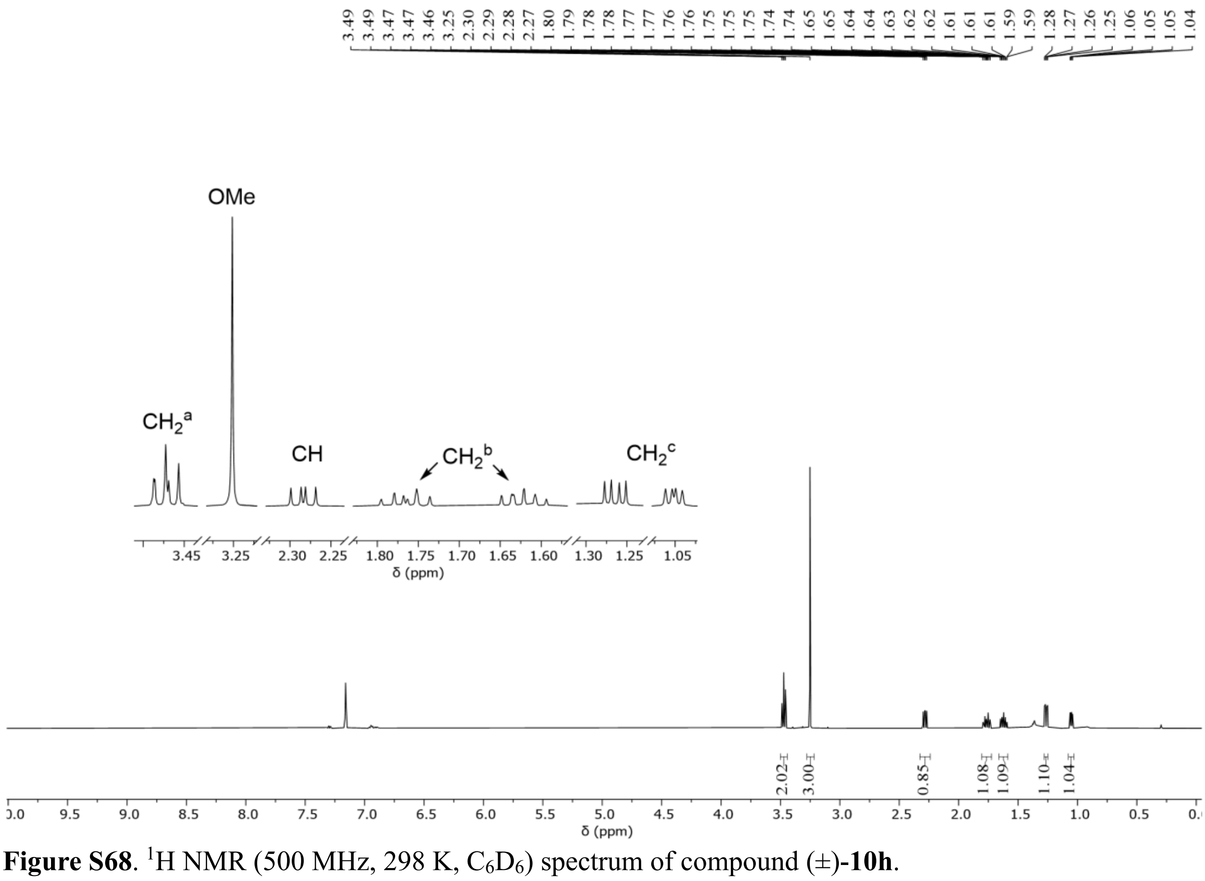

Figure S68. ^{1}H NMR (500 MHz, 298 K, C_6D_6) spectrum of compound (±)-**10h**.

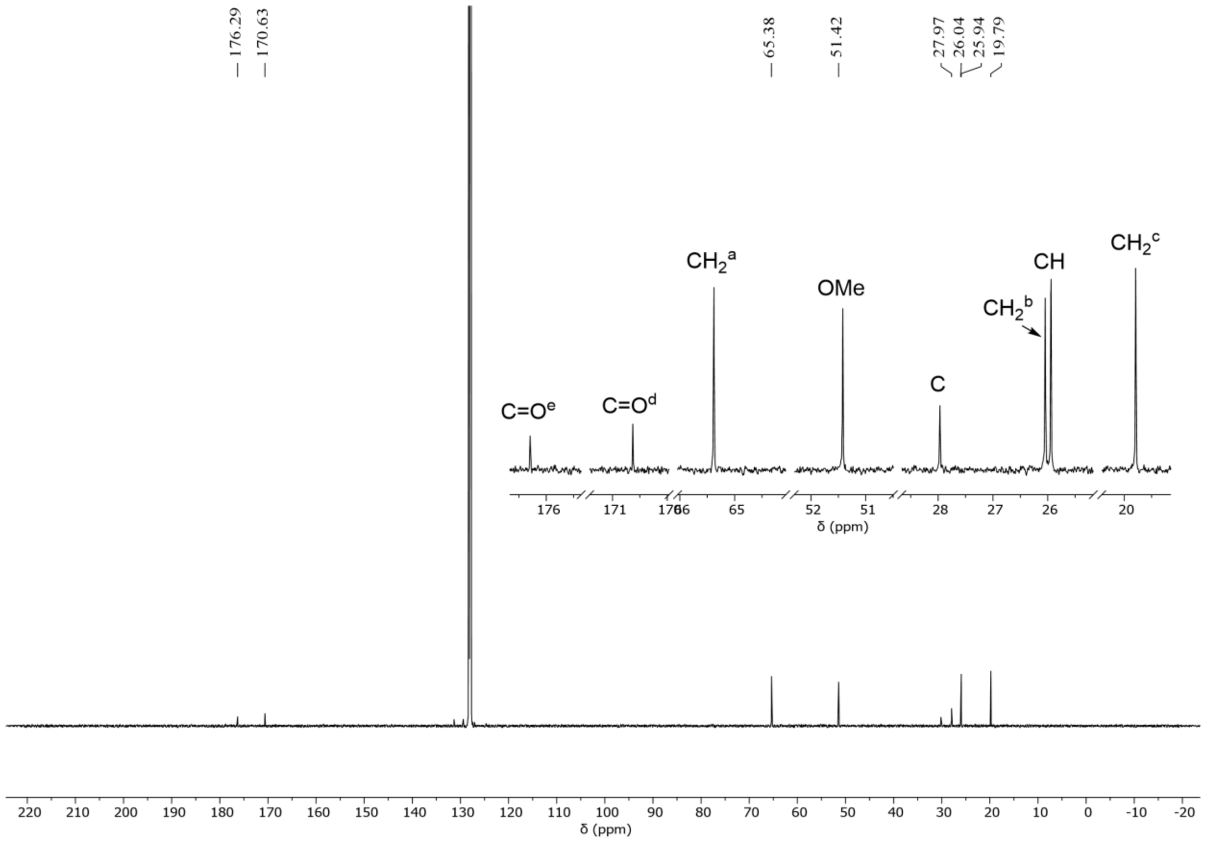

Figure S69. $^{13}C\{^1H\}$ NMR (125 MHz, 298 K, C_6D_6) spectrum of compound (±)-**10h**.

Synthesis of compound (±)-10i

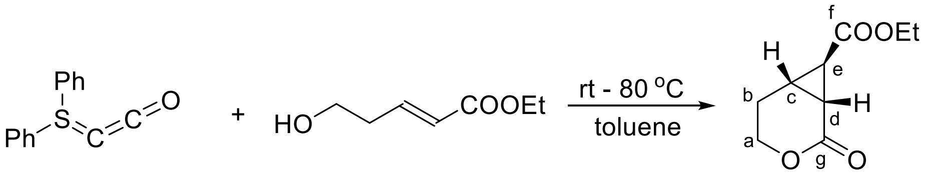

1 **8i** (±)-**10i**

A solution of compound **8i** (28.8 mg, 0.2 mmol) in toluene (1.0 mL) was added dropwise to a solution of compound **1** (45.2 mg, 0.2 mmol) in toluene (0.5 mL) and kept at room temperature for 12 hours, and then at 80 °C for another 12 hours. All the volatiles were removed under reduced pressure, the remaining residue was purified by silica gel column chromatography (eluent: *n*-pentane/EtOAc = 4:1) to give compound (±)-**10i** (13.6 mg, 0.074 mmol, 37%) as a colorless solid.

Characterization data of compound (±)-**10i**:

^{1}H NMR (500 MHz, 298 K, C_6D_6): δ = 3.83 (m, 2H, CH_2^{Et}), [3.27, 2.94](each m, each 1H, CH_2^a), 2.42 (m, 1H, CH^d), 2.00 (m, 1H, CH^e), 1.55 (CH^c), [1.08, 0.71](each , each 1H, CH_2^b), 0.88 (t, $^3J_{HH}$ = 7.1 Hz, 3H, CH_3^{Et}).
^{13}C{^{1}H} NMR (125 MHz, 298 K, C_6D_6): δ = 170.0 (C=O^f), 166.1 (C=O^g), 63.2 (CH_2^a), 61.0 (CH_2^{Et}), 24.7 (CH^d), 22.1 (CH^c), 21.2 (CH^e), 19.1 (CH_2^b), 14.0 (CH_3^{Et}).
IR (ATR) [cm^{-1}]: ṽ = 2989, 1439, 1402, 1341, 1301, 1268, 1219, 1190, 1111, 1072, 1044, 1006, 915, 862, 783, 722, 657.
HR-MS-ESI(+) calc. $C_9H_{12}NaO_4^+$ [M+Na]$^+$ 207.0628, found 207.0626.

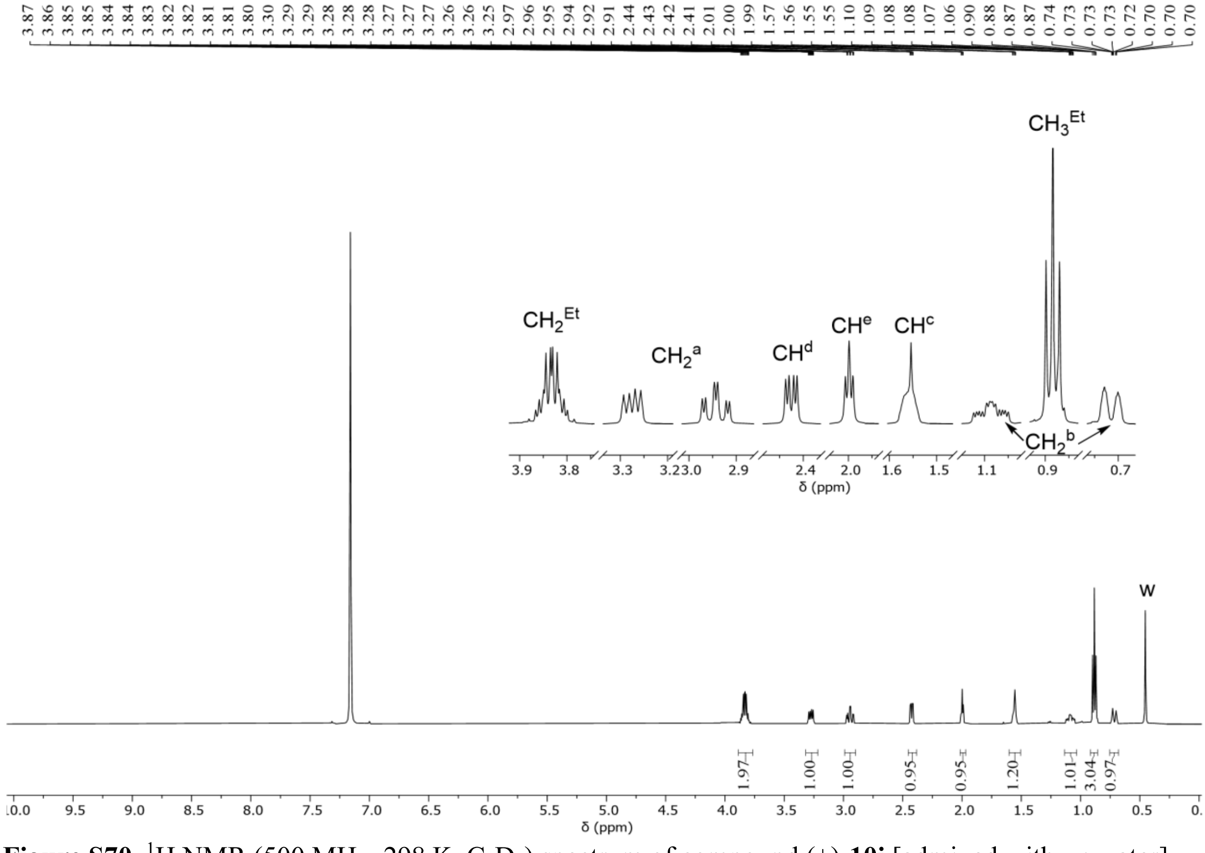

Figure S70. ^{1}H NMR (500 MHz, 298 K, C_6D_6) spectrum of compound (±)-**10i** [admixed with w: water].

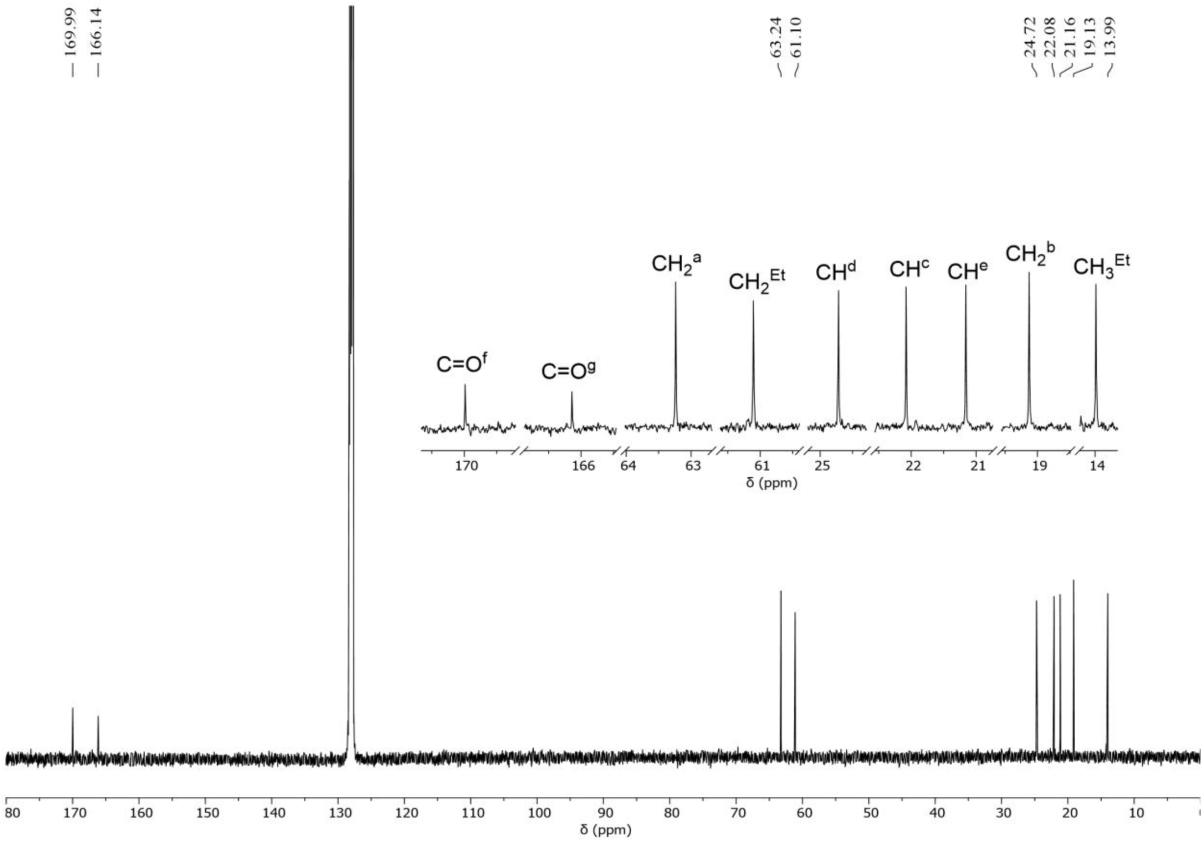

Figure S71. $^{13}C\{^{1}H\}$ NMR (125 MHz, 298 K, C_6D_6) spectrum of compound (±)-**10i**.

Synthesis of compound (±)-10j

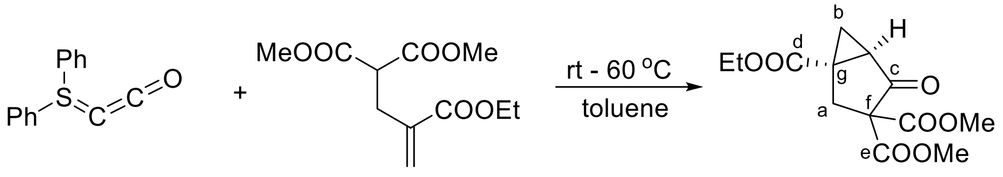

1 **8j** (±)-**10j**

A solution of compound **8j** (48.8 mg, 0.2 mmol) in toluene (1.0 mL) was added dropwise to a solution of reagent **1** (45.2 mg, 0.2 mmol) in toluene (0.5 mL) and kept at room temperature for 24 hours, and then at 60 °C for another 12 hours. All the volatiles were removed under reduced pressure, the remaining residue was purified by silica gel column chromatography (eluent: *n*-pentane/EtOAc = 4:1) to give compound (±)-**10j** (38.1 mg, 0.134 mmol, 67%) as a colorless solid. Crystals of compound (±)-**10j** suitable for X-ray diffraction were obtained from a solution of the colorless solid in EtOAc and *n*-pentane (ratio: 1:4) at rt.

Characterization data of compound (±)-**10j**:

m.p. 80 °C.
^{1}H NMR (500 MHz, 298 K, C_6D_6): δ = 3.77 (m, 2H, CH_2^{Et}), [3.63, 2.96](each m, each 1H, CH_2^{a}), [3.25, 3.23](each s, each 3H, OMe), 2.28 (m, 1H, CH), [1.55, 1.04](each m, each 1H, CH_2^{b}), 0.80 (t, $^3J_{HH}$ = 7.1 Hz, CH_3^{Et}).
^{13}C{^{1}H} NMR (125 MHz, 298 K, C_6D_6): δ = 197.9 (C=O^c), 170.0 (C=O^d), [167.9, 167.5](C=O^e), 66.4 (C^f), 61.2 (CH_2^{Et}), [53.1, 52.9](OMe), 36.2 (CH), 32.6 (CH_2^{a}), 31.5 (C^g), 19.8 (CH_2^{b}), 13.9 (CH_3^{Et}).
IR (ATR) [cm^{-1}]: ṽ = 2963, 1733, 1438, 1381, 1258, 1207, 1147, 1112, 1077, 1015, 947, 756.
HR-MS-ESI(+) calc. $C_{13}H_{16}NaO_7^+$ $[M+Na]^+$ 307.0788, found 307.0789.

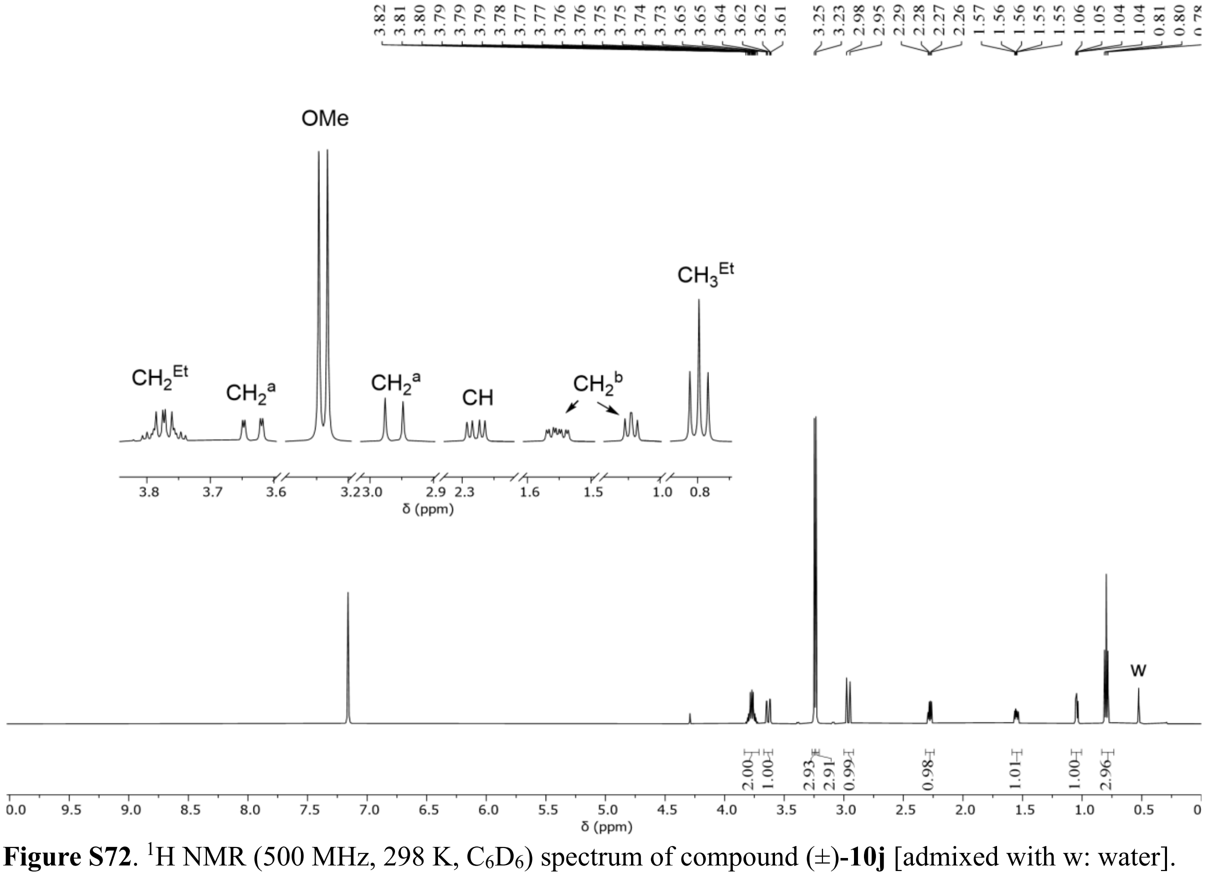

Figure S72. ^{1}H NMR (500 MHz, 298 K, C_6D_6) spectrum of compound (±)-**10j** [admixed with w: water].

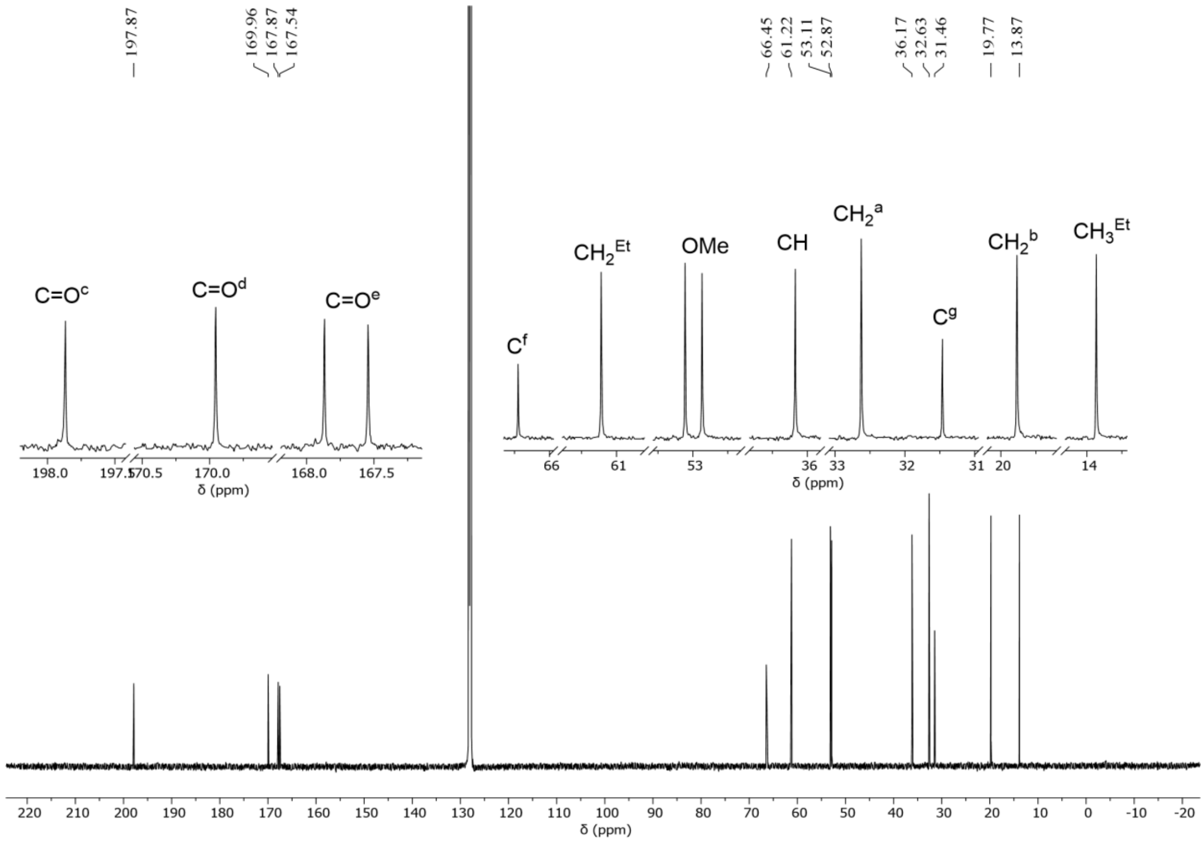

Figure S73. $^{13}C\{^{1}H\}$ NMR (125 MHz, 298 K, C_6D_6) spectrum of compound (±)-**10j**.

Synthesis of compound (±)-10k

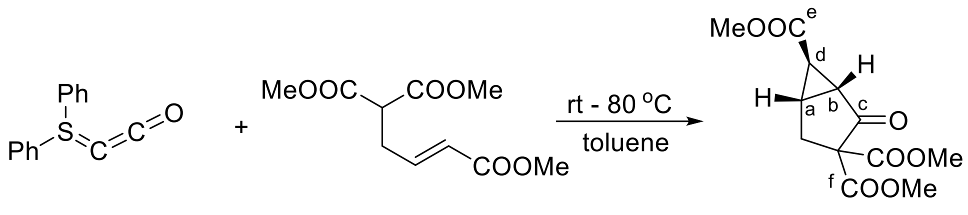

1 **8k** (±)-**10k**

A solution of compound **8k** (46.0 mg, 0.2 mmol) in toluene (1.0 mL) was added dropwise to a solution of reagent **1** (45.2 mg, 0.2 mmol) in toluene (0.5 mL) and kept at room temperature for 24 hours, and then at 80 °C for another 12 hours. All the volatiles were removed under reduced pressure, the remaining residue was purified by silica gel column chromatography (eluent: *n*-pentane/EtOAc = 3:1) to give compound (±)-**10k** (29.7 mg, 0.110 mmol, 55%) as a colorless oil.

Characterization data of compound (±)-**10k**:

^{1}H NMR (500 MHz, 298 K, C_6D_6): δ = [3.26, 3.20](each s, each 3H, OMe^f), 3.19 (s, 3H, OMe^e), [2.80, 2.52](each m, each 1H, CH_2), 2.32 (m, 1H, CH^b), 2.04 (m, 1H, CH^d), 1.99 (m, 1H, CH^a).
^{13}C{^{1}H} NMR (125 MHz, 298 K, C_6D_6): δ = 198.7 (C=O^c), 169.5 (C=O^e), [168.1, 167.5](C=O^f), 65.2 (C), [53.2, 53.0](OMe^f), 51.8 (OMe^e), 35.6 (CH^b), 32.4 (CH_2), 26.9 (CH^a), 26.6 (CH^d).
IR (ATR) [cm^{-1}]: ṽ = 2961, 1729, 1439, 1399, 1331, 1269, 1201, 1180, 1156, 1088, 1052, 990, 952, 907, 849, 801, 541.
HR-MS-ESI(+) calc. $C_{12}H_{14}NaO_7^+$ [M+Na]$^+$ 293.0632, found 293.0634.

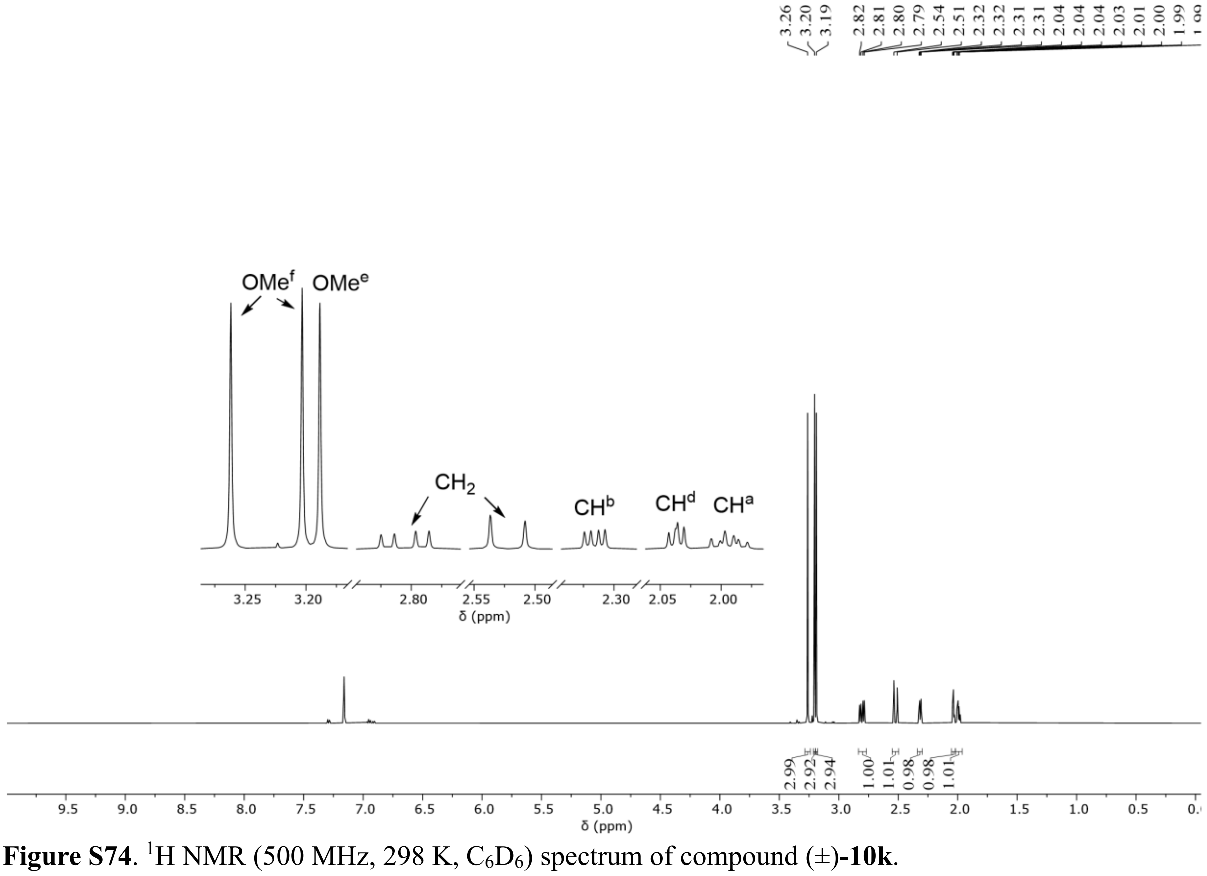

Figure S74. ^{1}H NMR (500 MHz, 298 K, C_6D_6) spectrum of compound (±)-**10k**.

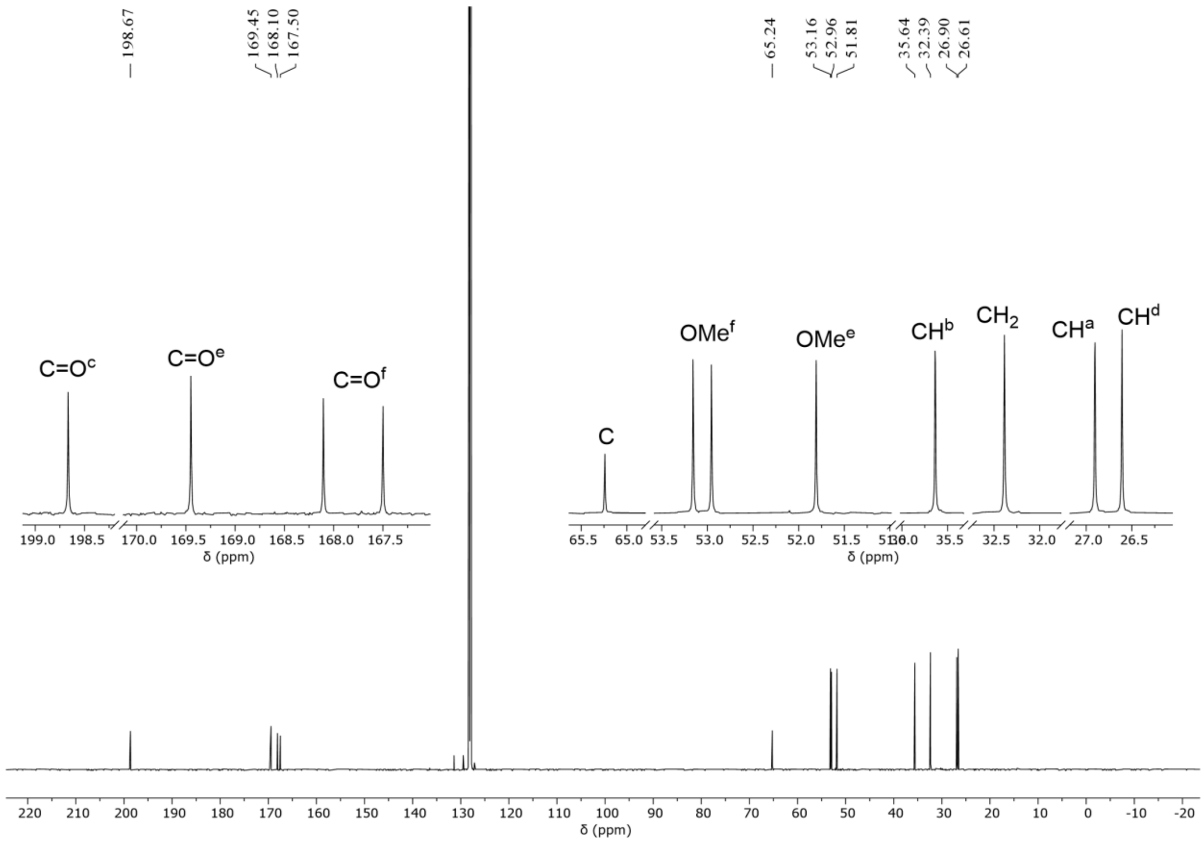

Figure S75. $^{13}C\{^{1}H\}$ NMR (125 MHz, 298 K, C_6D_6) spectrum of compound (±)-**10k**.

X-ray crystallography

Diffraction data were collected on a Bruker D8 Venture four-circle-diffractometer from Bruker AXS equipped with Photon II, Photon III or Bruker PHOTON III 14 detectors. The X-ray sources were a microfocus IμS Cu/Mo from Incoatec equipped with HELIOS mirror optics and single-hole collimator from Bruker AXS. The software used was the APEX5 (2023.9-2)[13] suite and therein integrated programs SAINT (integration)[14] und SADABS (absorption correction) from Bruker AXS. Structure solution was accomplished with SHELXT[15]. Unless otherwise noted SHELXL (2019/3) was used for refinement against F^2 until convergence using the full-matrix least-squares method[15]. All non-hydrogen atoms were refined with anisotropic atomic displacement parameters. The hydrogen atoms were refined isotropically on calculated positions using a riding model with $U_H = 1.5U_C$ for methyl groups, otherwise $U_H = 1.2U_C$. Disordered structure refinement was applied to treat disordered solvent molecules[16]. Further details on the individual data sets are tabulated in the following structure tables and the special refinement details sections. ShelXle3[17] and OLEX2[18] were used as the graphical user interface and FinalCIF (https://www.xs3.uni-freiburg.de/research/finalcif) was used for data finalization. Special Utilities included a SMZ1270 stereomicroscope from Nikon Metrology and a Leica M205M Stereomicroscope which were used for sample preparation. Crystals were mounted on MicroMounts or MicroLoops from MiTeGen using NVH (noise, vibration and harshness) immersion oil. Crystals were cooled to the reported temperature with Cryostream 800 from Oxford Cryosystems.

The crystal structure of **1** was initially refined by independent atom model (IAM) refinement using SHELXL. The final structure refinement was performed with aspherical atomic form factors using NoSpherA2[19,20] in OLEX2.[21] Hirshfeld-partitioned electron density was calculated with ORCA (Version 5.0)[22] using the B3LYP hybrid functional[23] and the def2-TZVPP basis set.[24] Positions and isotropic atomic displacement parameters were refined freely for all H atoms. Figures were drawn with Mercury.[25]

(±)-**6ca** crystallizes in the chiral (Sohnke) space group $P2_12_12_1$ [No. 19]. The Flack parameter refines to 0.28(3) determined using 1725 quotients [(I+)-(I-)]/[(I+)+(I-)][26]. The large value of the Flack parameter together with its small standard uncertainty indicates that the crystal studied is a partial inversion twin rather than a conglomerate.

There are two independent molecules in the structure of **10f** which are predominately conformers about the S-N bond. In molecule 1 the tosyl substituent is positioned on the same side of the pyrrolidin-2-one ring as the cyclopropane ring. In molecule 2 tolyl group of the tosylate substituent and the cyclopropane ring reside on opposite sides of the pyrrolidin-2-one ring.

For **10f** and **10g**, the diffraction data were scaled and multi-scan absorption corrected using TWINABS-2012/1.[27] The reflections of both components were used for subsequent refinement as a two-component twin. The ratio of the major to minor component refined to 0.9267(7):0.0733(7) for **10f** and 0.6683(13):0.3317(13) for **10g**. The crystal structure of **10f** contains solute benzene crystallized about a centre of symmetry, so the constitution of the crystal is $C_{20}H_{18}N_2O_3S\cdot0.5(C_6H_6)$.

Structure Tables

Structure	**1**	**6ca**	**10b**
CCDC number	2480574	2480575	2480576
Empirical formula	$C_{14}H_{10}OS$	$C_{20}H_{26}O_6$	$C_{14}H_{15}NO_5S$
Formula weight	226.28	362.41	309.33
Temperature [K]	100.00	100.00	100.00
Crystal system	monoclinic	Orthorhombic	monoclinic
Space group (number)	$P2_1/n$ (14)	$P2_12_12_1$ (19)	$P2_1/c$ (14)
a [Å]	6.3568(13)	5.9083(2)	16.4652(2)
b [Å]	7.6612(18)	9.6478(4)	14.0253(2)
c [Å]	22.863(7)	34.4972(13)	12.3666(2)
α [°]	90	90	90
β [°]	93.844(7)	90	90.2870(10)
γ [°]	90	90	90
Volume [Å^3]	1110.9(5)	1966.41(13)	2855.78(7)
Z	4	4	8
ρ_{calc} [gcm^{-3}]	1.353	1.224	1.439
μ [mm^{-1}]	0.263	0.740	2.222
F(000)	472	776	1296
Crystal size [mm^3]	0.15×0.15×0.05	0.2×0.1×0.01	0.1×0.1×0.01
Crystal colour	colourless	colourless	colourless
Crystal shape	plate	block	plate
Radiation	MoK_α (λ=0.71073 Å)	CuK_α (λ=1.54178 Å)	CuK_α (λ=1.54178 Å)
2Θ range [°]	6.41 to 59.14 (0.72 Å)	5.12 to 158.81 (0.78 Å)	5.37 to 144.85 (0.81 Å)
Index ranges	-8 ≤ h ≤ 8<br>-10 ≤ k ≤ 10<br>-31 ≤ l ≤ 31	-7 ≤ h ≤ 7<br>-12 ≤ k ≤ 12<br>-43 ≤ l ≤ 43	-20 ≤ h ≤ 20<br>-15 ≤ k ≤ 17<br>-13 ≤ l ≤ 15
Reflections collected	29683	62249	28689
Independent reflections	3109<br>R_{int} = 0.0567<br>R_{sigma} = 0.0300	4253<br>R_{int} = 0.0347<br>R_{sigma} = 0.0116	5628<br>R_{int} = 0.0657<br>R_{sigma} = 0.0428
Completeness	99.9 %	100.0 %	99.9 %
Data / Restraints / Parameters	3109/0/145	4253/0/241	5628/0/383
Goodness-of-fit on F^2	1.095	1.066	1.015
Final R indexes [I≥2σ(I)]	R_1 = 0.0426<br>wR_2 = 0.0898	R_1 = 0.0262<br>wR_2 = 0.0665	R_1 = 0.0376<br>wR_2 = 0.0819
Final R indexes [all data]	R_1 = 0.0520<br>wR_2 = 0.0936	R_1 = 0.0266<br>wR_2 = 0.0670	R_1 = 0.0572<br>wR_2 = 0.0913
Largest peak/hole [eÅ^{-3}]	0.37/-0.33	0.23/-0.21	0.40/-0.46
Flack X parameter		0.28(3)	
Extinction coefficient			

Structure	**10f**	**10g**	**10j**
CCDC number	2480577	2480578	2480579
Empirical formula	$C_{23}H_{21}N_2O_3S$	$C_{15}H_{17}NO_5S$	$C_{13}H_{16}O_7$
Formula weight	405.48	323.35	284.26
Temperature [K]	100.00	100.00	100.00
Crystal system	monoclinic	monoclinic	triclinic
Space group (number)	$P2_1/c$ (14)	$P2_1/c$ (14)	$P\overline{1}$ (2)
a [Å]	22.8102(13)	17.5012(8)	8.5028(4)
b [Å]	10.2014(6)	7.9740(4)	9.4500(5)
c [Å]	8.5945(5)	11.3374(5)	10.1479(5)
α [°]	90	90	116.108(2)
β [°]	93.805(2)	107.2790(10)	107.248(2)
γ [°]	90	90	97.631(2)
Volume [Å^3]	1995.5(2)	1510.78(12)	665.51(6)
Z	4	4	2
ρ_{calc} [gcm^{-3}]	1.350	1.422	1.419
μ [mm^{-1}]	1.666	2.124	0.994
F(000)	852	680	300
Crystal size [mm^3]	0.2×0.2×0.02	0.075×0.05×0.01	0.1×0.1×0.05
Crystal colour	colourless	colourless	colourless
Crystal shape	plate	plate	plate
Radiation	CuK_α (λ=1.54178 Å)	CuK_α (λ=1.54178 Å)	CuK_α (λ=1.54178 Å)
2Θ range [°]	7.77 to 140.46 (0.82 Å)	5.29 to 136.68 (0.83 Å)	10.59 to 140.13 (0.82 Å)
Index ranges	-27 ≤ h ≤ 27<br>0 ≤ k ≤ 12<br>0 ≤ l ≤ 10	0 ≤ h ≤ 21<br>-9 ≤ k ≤ 0<br>-13 ≤ l ≤ 13	-10 ≤ h ≤ 10<br>-11 ≤ k ≤ 11<br>-12 ≤ l ≤ 12
Reflections collected	8217	5554	29153
Independent reflections	8217<br>R_{int} = 0.0602<br>R_{sigma} = 0.0273	5554<br>R_{int} = 0.0608<br>R_{sigma} = 0.0354	2541<br>R_{int} = 0.0350<br>R_{sigma} = 0.0141
Completeness	99.4 %	99.5 %	99.9 %
Data / Restraints / Parameters	8217/0/264	5554/0/202	2541/0/184
Goodness-of-fit on F^2	1.086	1.099	1.123
Final R indexes [I≥2σ(I)]	R_1 = 0.0516<br>wR_2 = 0.1304	R_1 = 0.0432<br>wR_2 = 0.1166	R_1 = 0.0452<br>wR_2 = 0.1272
Final R indexes [all data]	R_1 = 0.0615<br>wR_2 = 0.1478	R_1 = 0.0477<br>wR_2 = 0.1215	R_1 = 0.0464<br>wR_2 = 0.1282
Largest peak/hole [eÅ^{-3}]	0.53/-0.90	0.28/-0.41	0.51/-0.21
Flack X parameter			
Extinction coefficient			

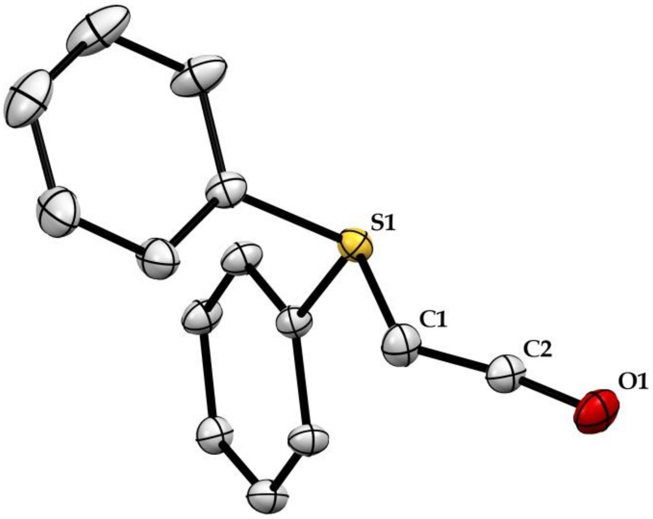

Figure S76. X-ray solid-state structure of compound **1**. Atomic displacement ellipsoids are shown at the 50% probability level. Hydrogen atoms omitted for clarity. Selected bond parameters in [Å] and [°]: S1-C1 1.6717(13), C1-C2 1.2767(16), C2-O1 1.1846(14), S1-C1-C2 125.95(10), C1-C2-O1 170.61(13).

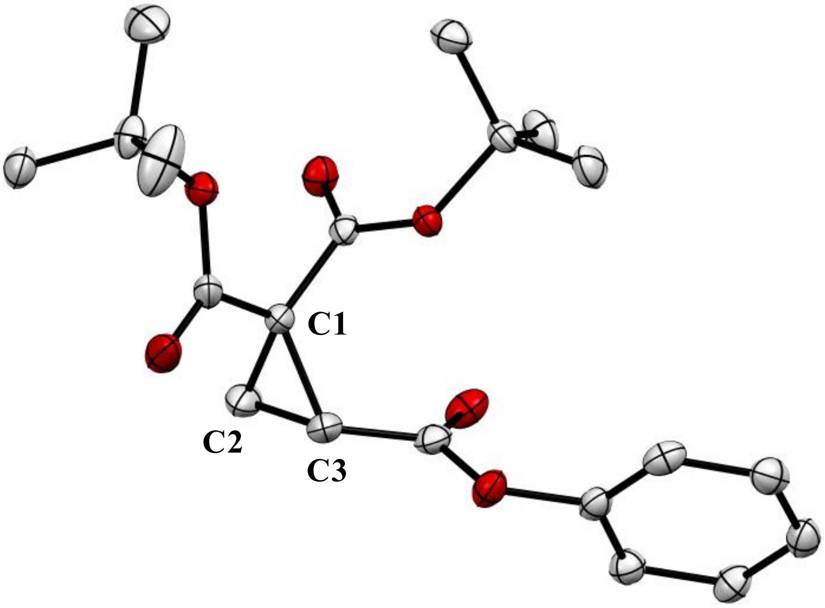

Figure S77. X-ray solid-state structure of compound (±)-**6ca**. Atomic displacement ellipsoids are shown at the 50% probability level. Hydrogen atoms omitted for clarity. Selected bond parameters in [Å] and [°]: C1-C2 1.507(2), C2-C3 1.499(2), C1-C3 1.5295(17), C1-C2-C3 61.17(9), C2-C3-C1 59.65(9), C3-C1-C2 59.18(9).

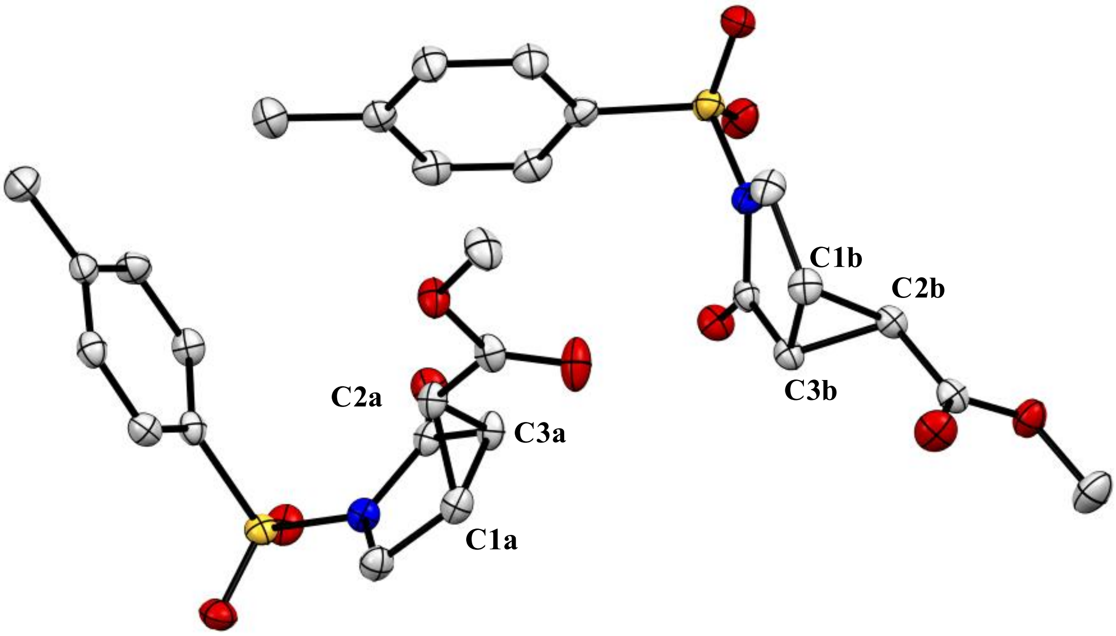

Figure S78. X-ray solid-state structure of compound (±)-**10b**. Atomic displacement ellipsoids are shown at the 50% probability level. Hydrogen atoms omitted for clarity. Selected bond parameters in [Å] and [°]: C1a-C2a 1.507(3), C2a-C3a 1.527(3), C1a-C3a 1.492(3), C1a-C2a-C3a 58.90(13), C2a-C3a-C1a 59.90(13), C3a-C1a-C2a 61.20(13), C1b-C2b 1.491(3), C2b-C3b 1.534(3), C1b-C3b 1.505(3), C1b-C2b-C3b 59.64(13), C2b-C3b-C1b 58.75(13), C3b-C1b-C2b 61.61(13).

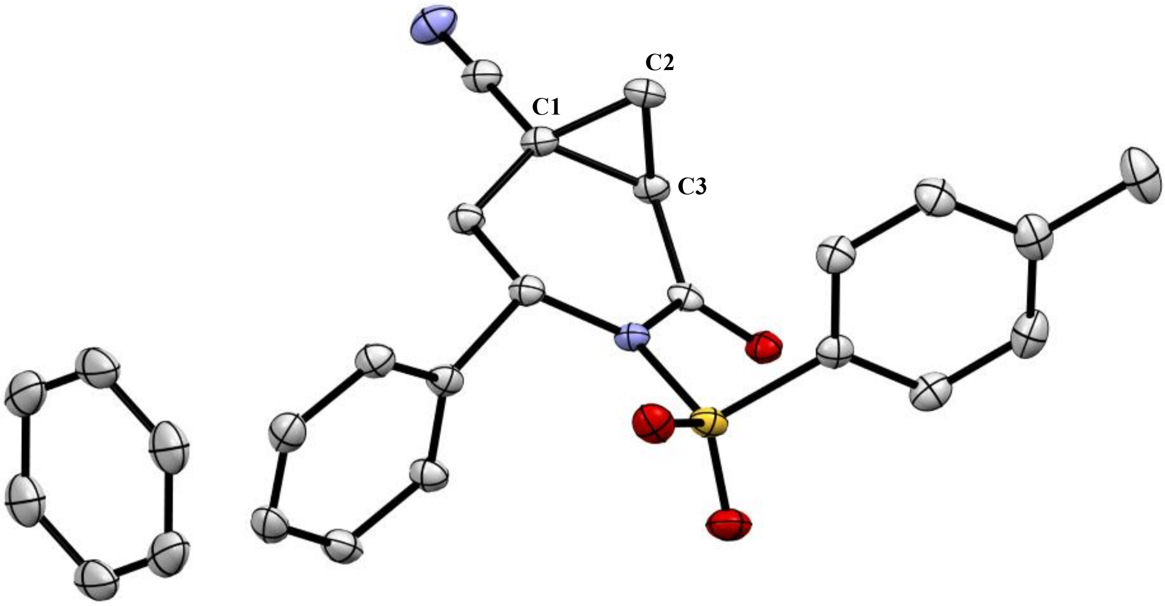

Figure S79. X-ray solid-state structure of compound (±)-**10f**. Atomic displacement ellipsoids are shown at the 50% probability level. Hydrogen atoms omitted for clarity. Selected bond parameters in [Å] and [°]: C1-C2 1.506(4), C2-C3 1.522(4), C1-C3 1.520(4), C1-C2-C3 60.25(17), C2-C3-C1 59.35(18), C3-C1-C2 60.40(18).

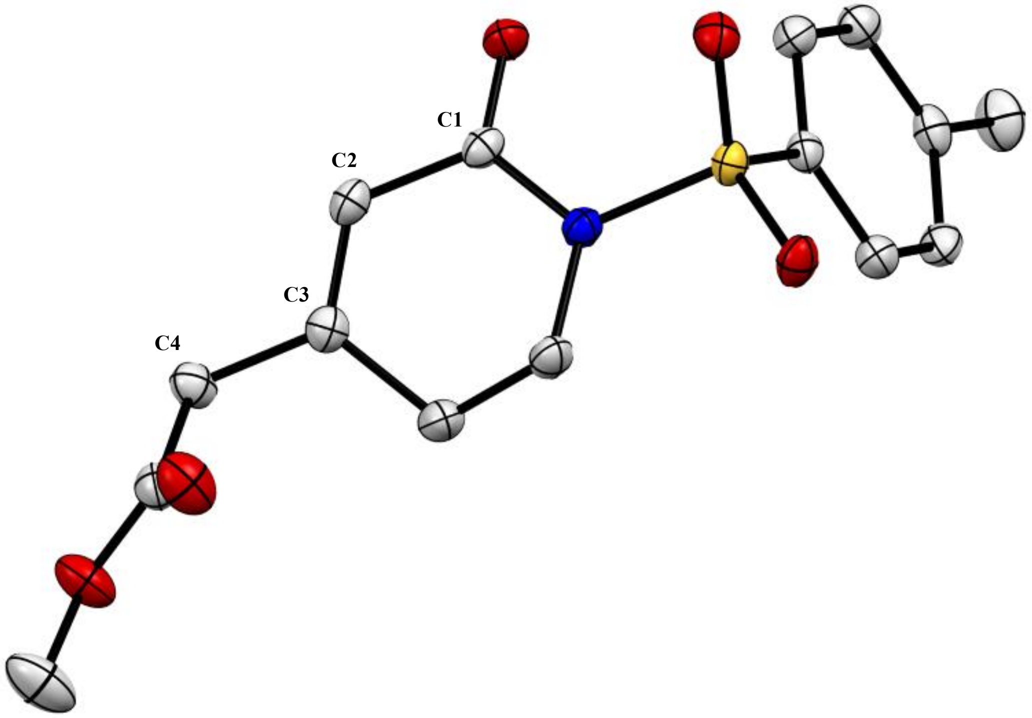

Figure S80. X-ray solid-state structure of compound **10g**. Atomic displacement ellipsoids are shown at the 50% probability level. Hydrogen atoms omitted for clarity. Selected bond parameters in [Å] and [°]: C1-C2 1.472(3), C2-C3 1.327(3), C3-C4 1.502(3), C1-C2-C3 122.7(2), C2-C3-C4 122.0(2).

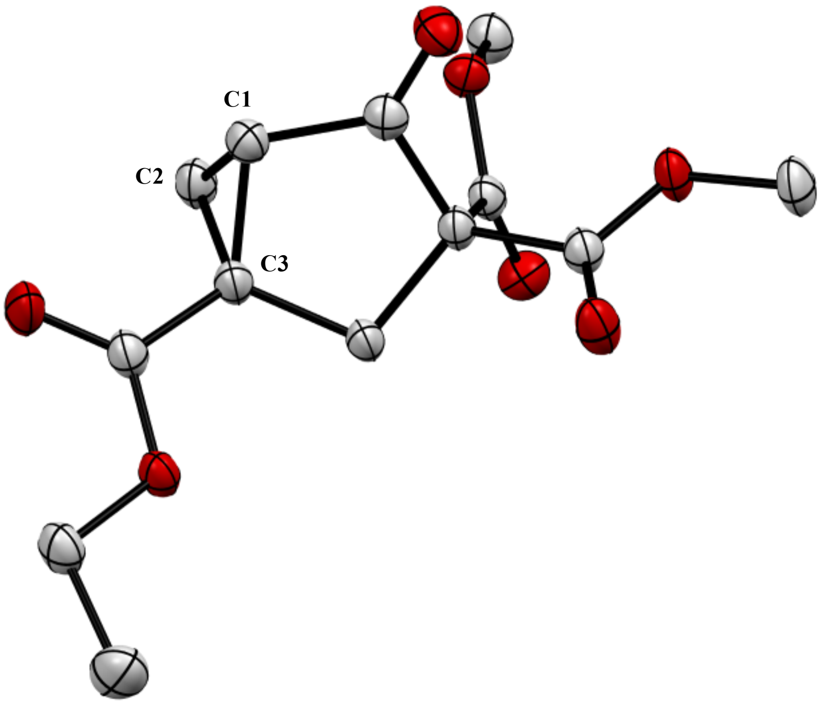

Figure S81. X-ray solid-state structure of compound (±)-**10j**. Atomic displacement ellipsoids are shown at the 50% probability level. Hydrogen atoms omitted for clarity. Selected bond parameters in [Å] and [°]: C1-C2 1.512(3), C2-C3 1.492(2), C1-C3 1.535(2), C2-C1-C3 58.62(11), C1-C2-C3 61.48(12), C1-C3-C2 59.90(12).

Computational details

All density functional theory (DFT) calculations were performed using the Gaussian 16 program package.[28] (Gaussian 16 Rev. C.01) employing the hybrid functional PBE0 with the def2-TZVP[29] basis set, defined by Weigend and Ahlrichs. In order to take dispersion effects into account, Grimme's D3 dispersion correction along with Becke-Johnson damping was used.[30,31] Solvents effects were considered implicitly with the SMD solvation model for THF. Gibbs free reaction energies and enthalpies were calculated for standard conditions ($p = 1$ atm, $T = 298$ K). Geometries of stationary points were fully optimized without any symmetry constraints. To test for minima or saddle points, harmonic vibrational frequency calculations were performed. The absence of imaginary frequencies (NIMAG = 0) confirmed a minimum, while a transition state was confirmed by an imaginary frequency (NIMAG = 1). To check whether a transition state connects related minima, the intrinsic reaction coordinate (IRC) was calculated. XYZ-data were generated employing GaussView[32]. For the visualization of computed structures and frontier molecular orbitals (isovalue = 0.6) IboView[33] was used. Natural Population Analyses (NPA) charge calculation and Wiberg Bond Index (WBI) calculation were conducted using NBO7.[34]

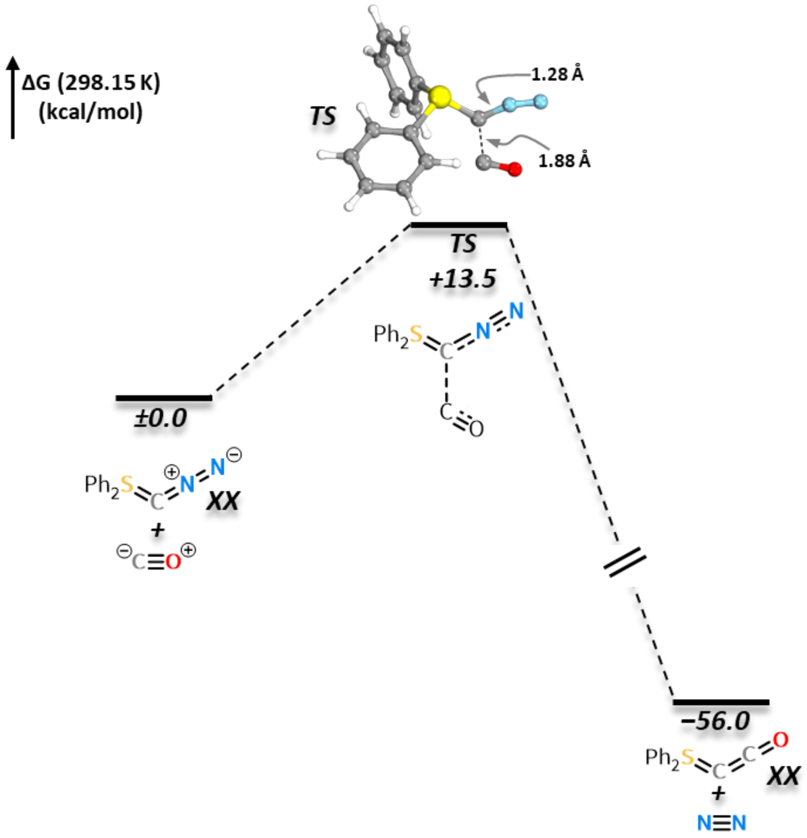

Figure S82. Computed energy profile at the PBE0-D3(BJ)/def2-TZVP/SMD(THF) level of theory for the N_2/CO exchange of diazo compound **A**.

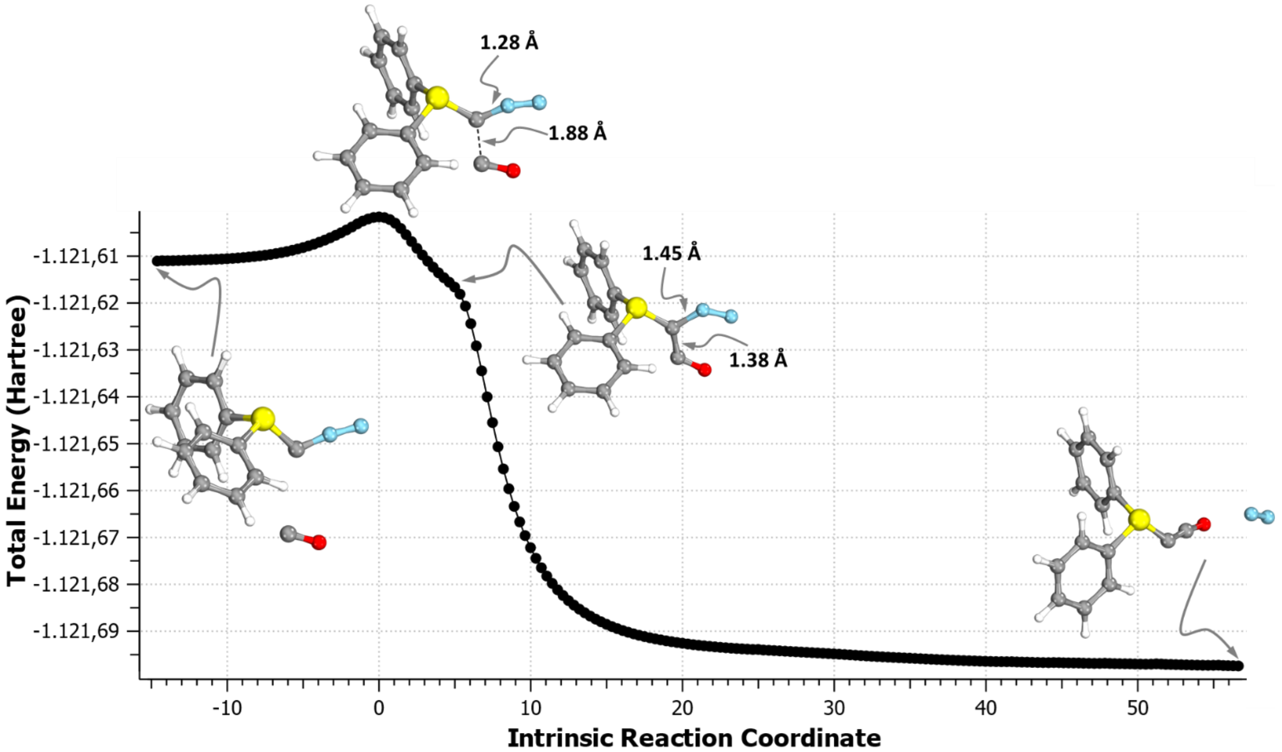

Figure S83. IRC calculation at the PBE0-D3(BJ)/def2-TZVP/SMD(THF) level of theory for the N_2/CO exchange of diazo compound **A**.

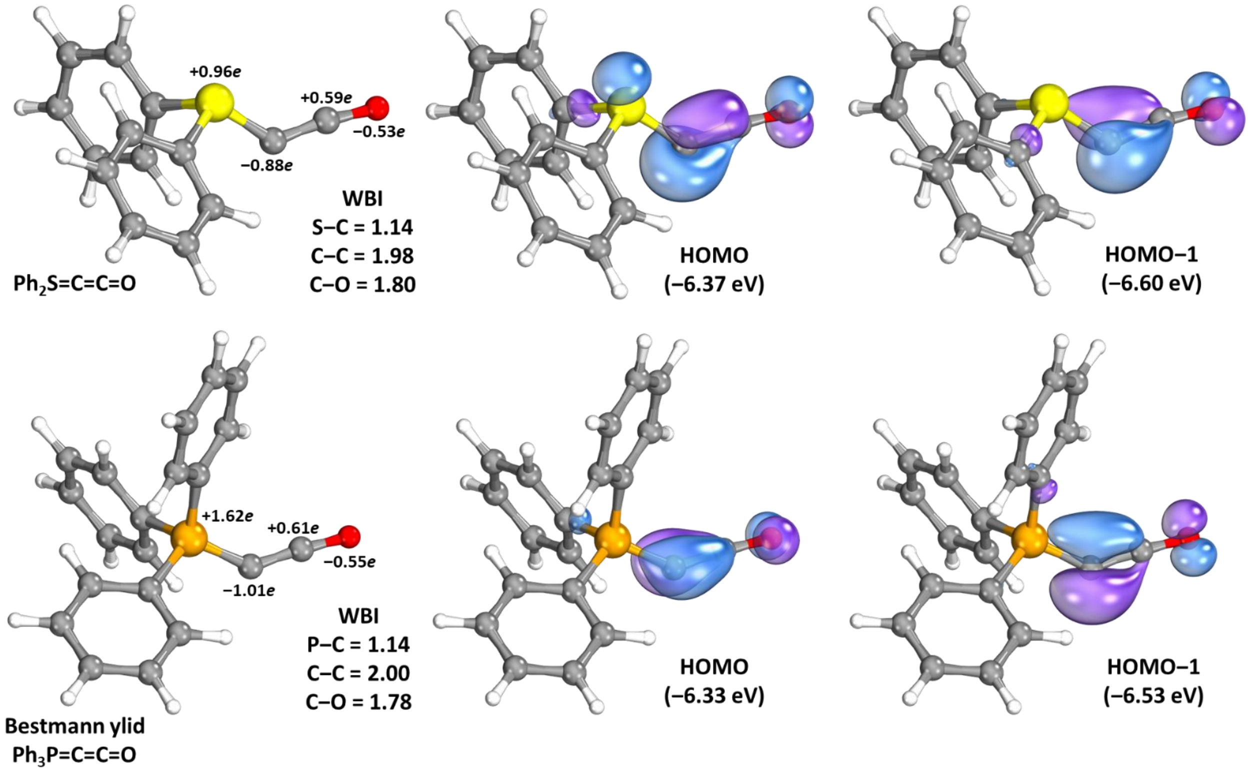

Figure S84. Comparison of the electronic structure (NPA charges, WBIs, and Kohn-Sham frontier orbitals (isovalue = 0.6)) of Ph_2SCCO and Bestmann's ylide Ph_3PCCO.

Diazo sulfur ylide **A** # opt freq PBE0-D3(BJ)/def2-TZVP SMD(THF)

C	-0.07351700	2.11610900	0.64266800
S	0.01177900	0.93022600	-0.57596100
C	1.35450600	-0.15250900	-0.13052800
C	2.36131100	-0.33392300	-1.06487900
C	1.39963000	-0.75372500	1.12040900
C	3.44102400	-1.14462500	-0.73888000
H	2.30454400	0.14938000	-2.03363000
C	2.48057800	-1.55846300	1.43277500
H	0.60227500	-0.59014300	1.83620300
C	3.49857400	-1.75425800	0.50453100
H	4.23527200	-1.29716600	-1.46028900
H	2.53120400	-2.03592300	2.40449700
H	4.34222000	-2.38651300	0.75700300
C	-1.37691000	-0.15182900	-0.26687800
C	-2.42344800	0.28446500	0.52349800
C	-1.40269200	-1.38973700	-0.89632500
C	-3.52063800	-0.54846300	0.70132200
H	-2.36471800	1.25780500	0.99777100
C	-2.50261300	-2.20976800	-0.71098000
H	-0.57278500	-1.71551500	-1.51360500
C	-3.56074500	-1.79086300	0.08761400
H	-4.34419800	-0.22076300	1.32530900
H	-2.53307400	-3.18045200	-1.19218900
H	-4.41894400	-2.43770800	0.22947400
N	0.20275200	4.40049900	-0.08190900
N	0.08001800	3.29934800	0.17315900

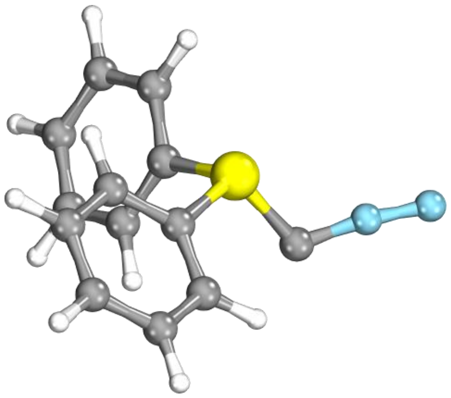

Figure S85. Computed structure of **A**.

Item	Value	Threshold	Converged?
Maximum Force	0.000009	0.000450	YES
RMS Force	0.000001	0.000300	YES
Maximum Displacement	0.000207	0.001800	YES
RMS Displacement	0.000051	0.001200	YES

Predicted change in Energy=-9.315441D-10
Optimization completed.
-- Stationary point found.

SCF Done: E(RPBE1PBE) = -1008.37944466 A.U. after 1 cycles

- Thermochemistry -

Zero-point correction= 0.196913 (Hartree/Particle)
Thermal correction to Energy= 0.210607
Thermal correction to Enthalpy= 0.211551
Thermal correction to Gibbs Free Energy= 0.153725
Sum of electronic and zero-point Energies= -1008.182532
Sum of electronic and thermal Energies= -1008.168838
Sum of electronic and thermal Enthalpies= -1008.167894
Sum of electronic and thermal Free Energies= -1008.225720

	E (Thermal)	CV	S
	KCal/Mol	Cal/Mol-Kelvin	Cal/Mol-Kelvin
Total	132.158	51.924	121.705

C01 [X(C13H10N2S1)]\NImag=0\\0.04372678,0.04072613,0.62267551,-0.02400

CO

opt freq PBE0-D3(BJ)/def2-TZVP SMD(THF)

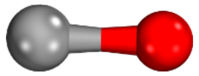

Figure S86. Computed structure of CO.

C	0.00000000	0.00000000	-0.64178800
O	0.00000000	0.00000000	0.48134100

Item	Value	Threshold	Converged?
Maximum Force	0.000030	0.000450	YES
RMS Force	0.000030	0.000300	YES
Maximum Displacement	0.000011	0.001800	YES
RMS Displacement	0.000016	0.001200	YES

Predicted change in Energy=-3.531558D-10
Optimization completed.
-- Stationary point found.

SCF Done: E(RPBE1PBE) = -113.226963732 A.U. after 1 cycles

- Thermochemistry -

Zero-point correction=	0.005099 (Hartree/Particle)
Thermal correction to Energy=	0.007460
Thermal correction to Enthalpy=	0.008404
Thermal correction to Gibbs Free Energy=	-0.014014
Sum of electronic and zero-point Energies=	-113.221865
Sum of electronic and thermal Energies=	-113.219504
Sum of electronic and thermal Enthalpies=	-113.218560
Sum of electronic and thermal Free Energies=	-113.240978

	E (Thermal)	CV	S
	KCal/Mol	Cal/Mol-Kelvin	Cal/Mol-Kelvin
Total	4.681	4.973	47.184

(C1O1)]|NImag=0||0.11273825,-0.36024489,1.15057727,-0.06420030,0.20505

TS

opt freq PBE0-D3(BJ)/def2-TZVP SMD(THF)

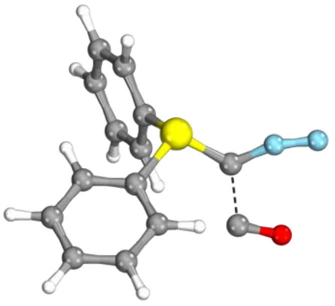

Figure S87. Computed structure of **TS**.

C	-0.58771800	1.74657000	-0.23834800
S	-0.03508100	0.36158300	-1.01616200
C	1.53029500	-0.08601300	-0.30020400
C	2.53788400	-0.45303700	-1.17855000
C	1.72705800	-0.04570600	1.07412200
C	3.77928200	-0.80042800	-0.66245200
H	2.35856800	-0.46761200	-2.24766200
C	2.97049100	-0.39166900	1.57233600
H	0.92151800	0.25786900	1.73386600
C	3.99204200	-0.77014600	0.70692600
H	4.57845300	-1.08993500	-1.33452700
H	3.14518200	-0.36528000	2.64158600

H	4.96305900	-1.03866400	1.10711000
C	-1.10297600	-0.94171700	-0.40990900
C	-2.43256200	-0.64198100	-0.16316300
C	-0.61448700	-2.23558900	-0.30625500
C	-3.28859700	-1.66479000	0.21531400
H	-2.78776500	0.37807100	-0.24769100
C	-1.48368200	-3.24735100	0.07163900
H	0.42736300	-2.45705700	-0.50353000
C	-2.81688200	-2.96439500	0.33363300
H	-4.32788500	-1.43918800	0.42380100
H	-1.11135300	-4.26081100	0.16551000
H	-3.48964500	-3.75936900	0.63384000
N	-0.96451200	3.93529700	-1.09930300
N	-0.72924100	2.84090400	-0.89121700
C	-1.05491300	1.94362300	1.56877900
O	-1.39891700	3.04237600	1.76459100

Item	Value	Threshold	Converged?
Maximum Force	0.000004	0.000450	YES
RMS Force	0.000001	0.000300	YES
Maximum Displacement	0.002675	0.001800	NO
RMS Displacement	0.000430	0.001200	YES

Predicted change in Energy=-1.576687D-09
Optimization completed on the basis of negligible forces.
-- Stationary point found.

SCF Done: E(RPBE1PBE) = -1121.60164452 A.U. after 1 cycles

- Thermochemistry -

Zero-point correction= 0.203447 (Hartree/Particle)
Thermal correction to Energy= 0.219495
Thermal correction to Enthalpy= 0.220439
Thermal correction to Gibbs Free Energy= 0.156400
Sum of electronic and zero-point Energies= -1121.398197
Sum of electronic and thermal Energies= -1121.382149
Sum of electronic and thermal Enthalpies= -1121.381205
Sum of electronic and thermal Free Energies= -1121.445245

	E (Thermal)	CV	S
	KCal/Mol	Cal/Mol-Kelvin	Cal/Mol-Kelvin
Total	137.735	58.899	134.783

5534,-3.0432185,0.5035961,-3.7490401|PG=C01 [X(C14H10N2O1S1)]|NImag=1|

Ph_2SCCO (1) # opt freq PBE0-D3(BJ)/def2-TZVP SMD(THF)

C	-0.06208500	2.15900600	0.29903600
S	0.01018700	0.87047700	-0.73823300
C	1.34968700	-0.17331800	-0.17605100
C	2.12763400	-0.78850900	-1.14262500
C	1.58893400	-0.34710300	1.17869700
C	3.17019100	-1.61270200	-0.73793100
H	1.92865400	-0.62449400	-2.19596600
C	2.63108600	-1.16955700	1.56891700
H	0.97269000	0.16386300	1.91005400
C	3.41861200	-1.80265000	0.61243100
H	3.79055900	-2.09859600	-1.48203500
H	2.83228900	-1.31776400	2.62369400
H	4.23527500	-2.44296300	0.92592300
C	-1.36903800	-0.18384500	-0.28889600
C	-2.27997400	0.23391500	0.66195200
C	-1.51050000	-1.39240700	-0.95715000
C	-3.35565100	-0.59024000	0.96589900
H	-2.13763000	1.19107700	1.15233400
C	-2.58656900	-2.20579400	-0.64424300
H	-0.78933300	-1.70108100	-1.70605000
C	-3.50796100	-1.80614600	0.31697600
H	-4.07539600	-0.27612800	1.71309300
H	-2.70781400	-3.15401100	-1.15509200
H	-4.34989900	-2.44568800	0.55624700
O	0.23052100	4.56006500	-0.07541800
C	0.10120700	3.39562100	0.02180000

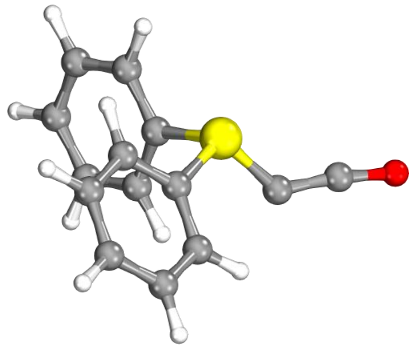

Figure S88. Computed structure of **1**.

Item	Value	Threshold	Converged?
Maximum Force	0.000005	0.000450	YES
RMS Force	0.000001	0.000300	YES
Maximum Displacement	0.001585	0.001800	YES
RMS Displacement	0.000467	0.001200	YES

Predicted change in Energy=-2.083973D-09
Optimization completed.
-- Stationary point found.

SCF Done: E(RPBE1PBE) = -1012.25459045 A.U. after 1 cycles

- Thermochemistry -

Zero-point correction= 0.197051 (Hartree/Particle)
Thermal correction to Energy= 0.210868
Thermal correction to Enthalpy= 0.211812
Thermal correction to Gibbs Free Energy= 0.152741
Sum of electronic and zero-point Energies= -1012.057540
Sum of electronic and thermal Energies= -1012.043722
Sum of electronic and thermal Enthalpies= -1012.042778
Sum of electronic and thermal Free Energies= -1012.101849

	E (Thermal)	CV	S
	KCal/Mol	Cal/Mol-Kelvin	Cal/Mol-Kelvin
Total	132.322	51.924	124.326

C14H10O1S1)]|NImag=0||0.05299093,0.05744075,0.89566251,-0.01609520,0.1

N_2

opt freq PBE0-D3(BJ)/def2-TZVP SMD(THF)

N	0.00000000	0.00000000	0.54449500
N	0.00000000	0.00000000	-0.54449500

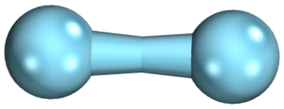

Figure S89. Computed structure of $\mathbf{N_2}$.

Item	Value	Threshold	Converged?
Maximum Force	0.000000	0.000450	YES
RMS Force	0.000000	0.000300	YES
Maximum Displacement	0.000000	0.001800	YES
RMS Displacement	0.000000	0.001200	YES

Predicted change in Energy=-1.326169D-16

Optimization completed.

-- Stationary point found.

SCF Done: E(RPBE1PBE) = -109.441264819 A.U. after 1 cycles

- Thermochemistry -

Zero-point correction=	0.005668 (Hartree/Particle)
Thermal correction to Energy=	0.008028
Thermal correction to Enthalpy=	0.008972
Thermal correction to Gibbs Free Energy=	-0.012754
Sum of electronic and zero-point Energies=	-109.435597
Sum of electronic and thermal Energies=	-109.433237
Sum of electronic and thermal Enthalpies=	-109.432292
Sum of electronic and thermal Free Energies=	-109.454018

	E (Thermal)	CV	S
	KCal/Mol	Cal/Mol-Kelvin	Cal/Mol-Kelvin
Total	5.038	4.970	45.726

C*(N1.N1)]|NImag=0||1.63996368,0.,-0.00014980,0.,0.,-0.00014980,-1.639

References

(1) Q. Sun, J.-N. Belting, J. Hauda, D. Tymann, P. W. Antoni, R. Goddard, M. M. Hansmann, *Science* **2025**, *387*, 885–892.
(2) K. G. Hugentobler, F.s Rebolledo, *Org. Biomol. Chem.* **2014**, *12*, 615–623.
(3) H. C. Wendlandt, J. A. Utley, B. J. Lee, T. D. Ho, K. L. Hull, *Org. Lett*. **2025**, *27*, 241–245.
(4) S. Mangelinckx, A. Žukauskaitė, V. Buinauskaitė, A. Šačkus, N. D. Kimpe, *Tetrahedron Lett*. **2008**, *49*, 6896–6900.
(5) F. Serpier, B. Flamme, J. Brayer, B. Folléas, and S. Darses, *Org. Lett.* **2015**, *17,* 1720–1723.
(6) L. Lai, W. Cai, Y. Huang, *Chem. Sci.,* **2024**, *15,* 11515–11520.
(7) T. J. Martin, V. G. Vakhshori, Y. S. Tran, O. Kwon, *Org. Lett.,* **2011**, *13,* 2586–2589.
(8) B. Jiang, F. Meng, Q. Liang, Y. Xu, T. Loh, *Org. Lett.* **2017**, *19,* 914−917.
(9) J. Gong, Q. Wang, J. Zhu, *J. Am. Chem.* **2023**, *145*, 15735−15741.
(10) Y. Chen, B. Shuai, C. Ma, X. Zhang, P. Fang, T. Mei, *Org. Lett.* **2017**, *19*, 2969–2972.
(11) A. Padwa, S. S. Murphree, Z. Ni, S. H. Watterson, *J. Org. Chem.* **1996**, *61*, 3829−3838.
(12) S. Ma, B. Xu, B. Ni, *J. Org. Chem.* **2000**, *65*, 8532−8543.
(13) Bruker APEX5. Bruker AXS Inc., Madison, Wisconsin, USA (2023).
(14) Bruker SAINT. Bruker AXS Inc., Madison, Wisconsin, USA (2012).
(15) G. M. Sheldrick, *Acta Cryst.* **2015**, *A71*, 3−8.
(16) (a) D. Kratzert, J. J. Holstein, I. Krossing, *J. Appl. Cryst*. **2015**, *48*, 933−938. (b) D. Kratzert, I. Krossing, *J. Appl. Cryst*. **2018**, *51*, 928−934.
(17) C. B. Hübschle, G. M. Sheldrick, B. Dittrich, *J. Appl. Cryst*. **2011**, *44*, 1281−1284.
(18) O.V. Dolomanov, L. J. Bourhis, R. J. Gildea, J. A. K. Howard, H. Puschmann, *J. Appl. Cryst*. **2009**, *42*, 339−341.
(19) F. Kleemiss, O. V. Dolomanov, M. Bodensteiner, N. Peyerimhoff, M. Midgley, L. J. Bourhis, A. Genoni, L. A. Malaspina, D. Jayatilaka, J. L. Spencer, F. White, B. Grundkoetter-Stock, S. Steinhauer, D. Lentz, H. Puschmann, S. Grabowsky, *Chem. Sci*. **2021**, *12*, 1675−1692.
(20) L. Midgley, L. J. Bourhis, O. V. Dolomanov, S. Grabowsky, F. Kleemiss, H. Puschmann, N. Peyerimhoff, *Acta Cryst*. **2021**, *A77*, 519−533.
(21) L. J. Bourhis, O. V. Dolomanov, R. J. Gildea, J. A. K. Howard, H. Puschmann, *Acta Cryst*. **2015**, *A71*, 59−75.
(22) F. Neese, F. Wennmohs, U. Becker, C. Riplinger, *J. Chem*. *Phys*. **2020**, *152*, 224108.
(23) (a) A. D. Becke, *J. Chem. Phys*. **1993**, *98*, 5648−5652; (b) C. Lee, W. Yang, R. G. Parr, *Phys. Rev*. **1988**, *B37*, 785−789.
(24) F. Weigend, R. Ahlrichs, *Phys. Chem. Chem. Phys*. **2005**, *7*, 3297−3305.
(25) C. F. Macrae, P. R. Edgington, P. McCabe, E. Pidcock, G. P. Shields, R. Taylor, M. Towler, J. van de Streek, *J. Appl. Cryst.* **2006**, *39*, 453−457.
(26) S., Parsons H. Flack, T. Wagner, *Acta Cryst*. **2013**, *B69*, 249−259.
(27) Bruker. TWINABS. Bruker AXS Inc., Madison, Wisconsin, USA (2012).
(28) Gaussian 16, Revision C.01, M. J. Frisch, G. W. Trucks, H. B. Schlegel, G. E. Scuseria, M. A. Robb, J. R. Cheeseman, G. Scalmani, V. Barone, G. A. Petersson, H. Nakatsuji, X. Li, M. Caricato, A. V. Marenich, J. Bloino, B. G. Janesko, R. Gomperts, B. Mennucci, H. P. Hratchian, J. V. Ortiz, A. F. Izmaylov, J. L. Sonnenberg, D. Williams-Young, F. Ding, F. Lipparini, F. Egidi, J. Goings, B. Peng, A. Petrone, T. Henderson, D. Ranasinghe, V. G. Zakrzewski, J. Gao, N. Rega, G. Zheng, W. Liang, M. Hada, M. Ehara, K. Toyota, R. Fukuda, J. Hasegawa, M. Ishida, T. Nakajima, Y. Honda, O. Kitao, H. Nakai, T. Vreven, K. Throssell, J. A. Montgomery, Jr., J. E. Peralta, F. Ogliaro, M. J. Bearpark, J. J. Heyd, E. N. Brothers, K. N. Kudin, V. N. Staroverov, T. A. Keith, R. Kobayashi, J. Normand, K. Raghavachari, A. P. Rendell, J. C. Burant, S. S. Iyengar, J. Tomasi, M. Cossi, J. M. Millam, M. Klene, C. Adamo, R. Cammi, J. W. Ochterski, R. L. Martin, K. Morokuma, O. Farkas, J. B. Foresman, and D. J. Fox, Gaussian, Inc., Wallingford CT, (2016).
(29) F. Weigend, R. Ahlrichs, *Phys. Chem. Chem. Phys.* **2005**, *7*, 3297–3305.
(30) a) L. Goerigk, S. Grimme, *J. Chem. Theory Comput.* **2011**, *7*, 291−309; b) S. Grimme, S. Ehrlich, L. Goerigk, *J. Comput. Chem.* **2011**, *32*, 1456−1465.
(31) E. R. Johnson, A. D. Becke, *J. Chem. Phys.* **2005**, *123*, 024101; b) E. R. Johnson, A. D. Becke, *J. Chem. Phys.* **2006**, *124*, 174104.
(32) GaussView, Version 6.1, Roy Dennington, Todd A. Keith, and John M. Millam, Semichem Inc., Shawnee Mission, KS, (2016).
(33) G. Knizia, IboView; see http://www.iboview.org.
(34) NBO 7.0. E. D. Glendening, J. K. Badenhoop, A. E. Reed, J. E. Carpenter, J. A. Bohmann, C. M. Morales, P. Karafiloglou, C. R. Landis, F. Weinhold Theoretical Chemistry Institute, University of Wisconsin, Madison, WI (2018).
